# Cooling-induced expansions of Afromontane forests in the Horn of Africa since the Last Glacial Maximum

**DOI:** 10.1038/s41598-023-37135-8

**Published:** 2023-06-26

**Authors:** Manuel Casas-Gallego, Karen Hahn, Katharina Neumann, Sebsebe Demissew, Marco Schmidt, Stéphanie C. Bodin, Angela A. Bruch

**Affiliations:** 1 Institute of Ecology, Diversity and Evolution, Goethe University Frankfurt, Frankfurt am Main, Germany; 2 Department of Geodynamics, Stratigraphy and Paleontology, Complutense University of Madrid, Madrid, Spain; 3 Department of Paleoanthropology, Senckenberg Research Institute, Frankfurt am Main, Germany; 4 National Herbarium of Ethiopia, Addis Ababa University, Addis Ababa, Ethiopia; 5 Palmengarten der Stadt Frankfurt am Main, Frankfurt am Main, Germany; 6 Research Centre “The role of culture in early expansions of humans” of the Heidelberg Academy of Sciences and Humanities, Senckenberg Research Institute, Frankfurt am Main, Germany

**Keywords:** Ecology, Environmental sciences

## Abstract

Understanding the changing plant ecosystems that existed in East Africa over the past millennia is crucial for identifying links between habitats and past human adaptation and dispersal across the region. In the Horn of Africa, this task is hampered by the scarcity of fossil botanical data. Here we present modelled past vegetation distributions in Ethiopia from the Last Glacial Maximum (LGM) to present at high spatial and temporal resolution. The simulations show that, contrary to long-standing hypotheses, the area covered by Afromontane forests during the Late Glacial was significantly larger than at present. The combined effect of low temperatures and the relative rainfall contribution sourced from the Congo Basin and Indian Ocean, emerges as the mechanism that controlled the migration of Afromontane forests to lower elevations. This process may have enabled the development of continuous forest corridors connecting populations that are currently isolated in mountainous areas over the African continent. Starting with the Holocene, the expansion of forests began to reverse. This decline intensified over the second half of the Holocene leading to a retreat of the forests to higher elevations where they are restricted today. The simulations are consistent with proxy data derived from regional pollen records and provide a key environmental and conceptual framework for human environmental adaptation research.

## Introduction

The Horn of Africa is a major biodiversity hotspot^1,2^, mainly as a result of considerable altitudinal contrasts which lead to extreme variability of temperature and rainfall and ultimately to the occurrence of diverse ecological conditions. Ethiopian vegetation is particularly diverse, comprising numerous endemic species and harboring the largest area of Afromontane forests in Africa^3,4^. These forests, together with more open savanna-type ecosystems, made up the mosaic of habitats that provided the essential resources for the subsistence of early modern humans from 200 thousand years before present (thereafter ka) onwards^5–9^. Later human populations have steadily interacted with, and benefited from, these ecosystems until today^10^. Thus, the understanding of past distributions of these habitats is of utmost importance for a range of research fields from paleoanthropology and archeology–in order to make hypotheses on plant resources availability for past human populations and their migrations into new landscapes–to botany, biogeography and conservation ecology.

**Figure 1:**
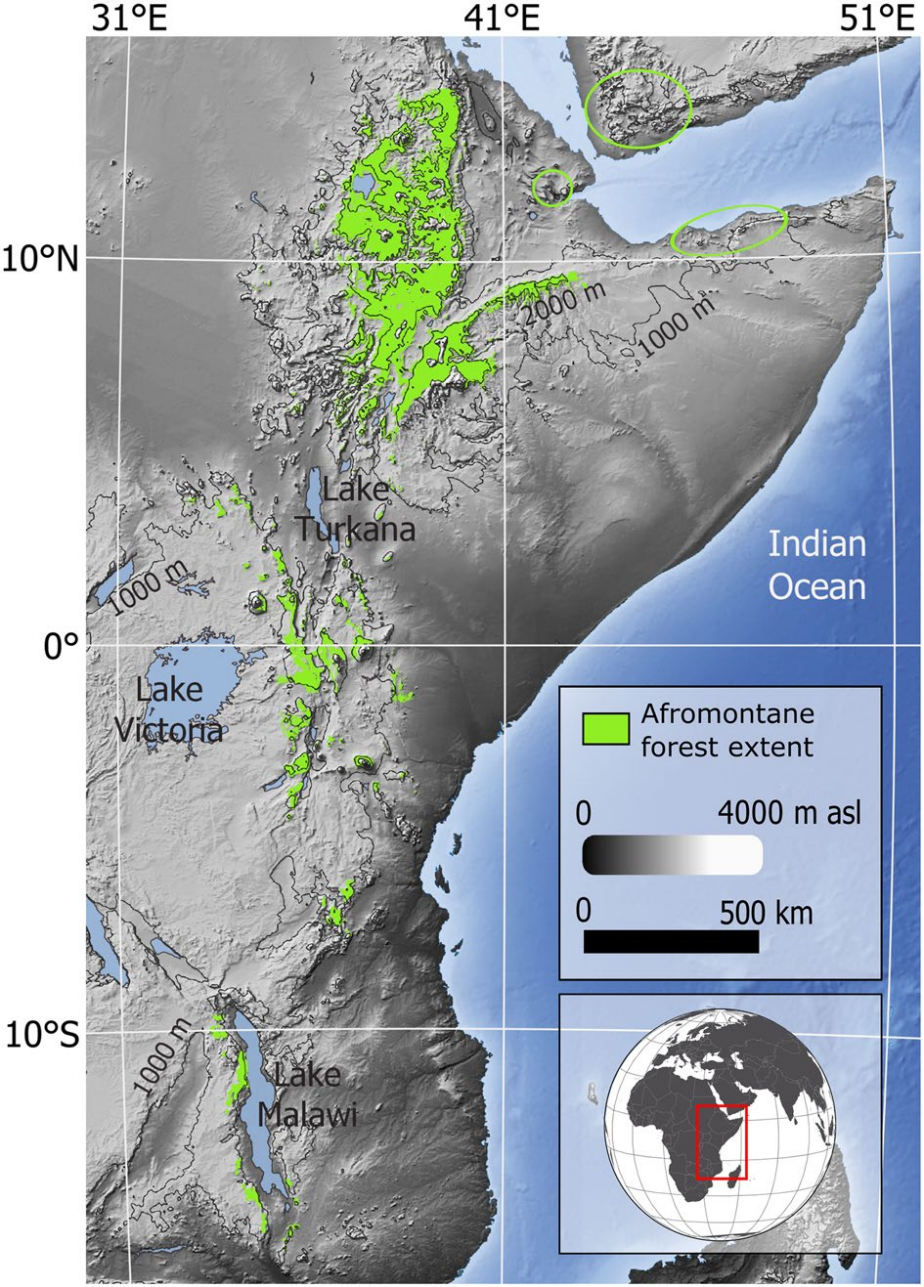
Current distribution of Afromontane forests in Eastern Africa characterized by intermountain isolation (see VECEA project). The green circles include patches of Afromontane habitats in Djibouti (Day forest), northern Somalia and south-western Yemen. Map created by the authors using QGIS v3.16.16 (URL: http://qgis.org).

Ethiopian plant ecosystems are among the most endangered in Africa due to habitat fragmentation and loss caused by deforestation and overexploitation^3,11^. Specifically, Afromontane forests have experienced a tremendous anthropogenic pressure as the area where they naturally occur is inhabited by the majority of the country’s population^12,13^. This type of forest also occurs with comparable floristic composition in other mountainous areas across East Africa forming an archipelago of vegetation^14^ (Fig. 1). This disjunct distribution pattern suggests an earlier connection of the currently isolated populations but the underlying conditions that may have enabled such a connection are not known so far.

Despite the importance of these ecosystems, little is known about their past spatial dynamics and their response to changing climatic conditions^15^. A number of pollen records furnish valuable information on the vegetation history of specific locations and time periods^16–21^ but do not allow for a larger scale reconstruction of vegetation and environments through time. Here we model past vegetation distributions in Ethiopia since the LGM (22 ka) using Ecological Niche Modelling (ENM). We use these simulations in combination with currently available fossil evidence from pollen archives to examine how these unique ecosystems were affected by past climate and atmospheric CO_2_ changes. Our approach has been made possible by the recent availability of modern detailed descriptions of potential vegetation in the region^22,23^, as well as by the advent of improved high-resolution paleoclimatic datasets^24^.

## Ethiopian climate and vegetation

Rainfall patterns in Ethiopia are complex and not yet fully understood^25^, although an overall gradient of increasing mean annual precipitation from northeast (200 mm) to southwest (> 2000 mm) is known to occur^26^. Rainfall is chiefly controlled by the seasonal north–south shift of the Intertropical Convergence Zone (ITCZ), and the country’s geographic proximity to the western Indian Ocean. In winter (December to February), the ITCZ is positioned south of Ethiopia, near the equator, resulting in low-moisture winds directed from the north-east and the occurrence of a dry season. In spring and summer, the ITCZ shifts northwards resulting in a wind direction reversal. As a consequence, prevailing south-westerly humid winds (West African Summer Monsoon) sourced from the Congo Basin bring rain across the territory, notably in the highlands. With some exceptions in the south-western highlands which receive rain throughout the year, the overall rainfall pattern is bimodal. Thus, a second lesser-magnitude rainy season occurs in autumn as a result of the passage of the ITCZ as it returns to its southern position. A second dry period of varying duration depending on the region usually occurs in late summer between the two rainy periods^22,27^.

The intricate topography of the Horn of Africa, with altitudes ranging from 125 m bsl at the Danakil depression up to 4533 m asl at the Semien Mountains, leads to extreme variability of temperatures. Mean annual temperatures are maximal at the lowlands, exceeding 30 °C in the Danakil Desert in the northern part of the country, as well as in the southern section of the Omo River, close to the area where it empties into Lake Turkana. The regional lapse rate is estimated to be between 0.5 and 0.7 °C per 100 m in altitude^28,29^.

Friis et al.^22^ defined and mapped out the main potential vegetation units (VUs) of Ethiopia, supported by extensive field observations. These VUs represent ecologically and climatically meaningful units of analysis (Supplementary Fig. S1), and can in turn be integrated into a broader phytogeographic framework of East Africa (see the VECEA project: https://vegetationmap4africa.org)^23^. Overall, highland vegetation is characterized by a series of altitudinal belts. Above 3200 m, an Afroalpine belt (AA) characterized by shrubby and herbaceous plants makes up the dominant vegetation. AA is home of a high number of endemic species, a significant proportion of which are threatened with extinction^11,22^. *Erica* shrub-dominated vegetation (EB) typically develops in a narrow altitudinal belt from 3000 to 3200 m. Between 1800 and 3000 m, climatic conditions enable the subsistence of Afromontane forests. *Juniperus*, *Podocarpus* and *Olea* are the most abundant trees in the dry variant of the Afromontane forest (DAF), which transitions into a denser and taller forest variant (moist Afromontane forest; MAF) in wetter settings, notably in south-western Ethiopia. The vegetation becomes more open as altitude decreases, comprising diverse savanna-type woodlands and bushlands adapted to dry conditions between 400 and 1800 m. Thus, in the Rift Valley and in areas to the east of it, various species of *Acacia* and *Commiphora* are dominant (ACB). At the same altitudinal range but to the west of the Rift Valley, deciduous woodlands characterized by *Combretum* and *Terminalia* (CTW) predominate with an abundance of grasses that are prone to fire^22^. Desert/semi-desert vegetation (DSS) occurs at altitudes below 400 m, where plant cover is sparse and consists of highly drought tolerant species, mainly small trees, shrubs and succulent and annual herbs^3,30^.

## Evolution of the Ethiopian vegetation units over the last 22,000 years

**Figure 2:**
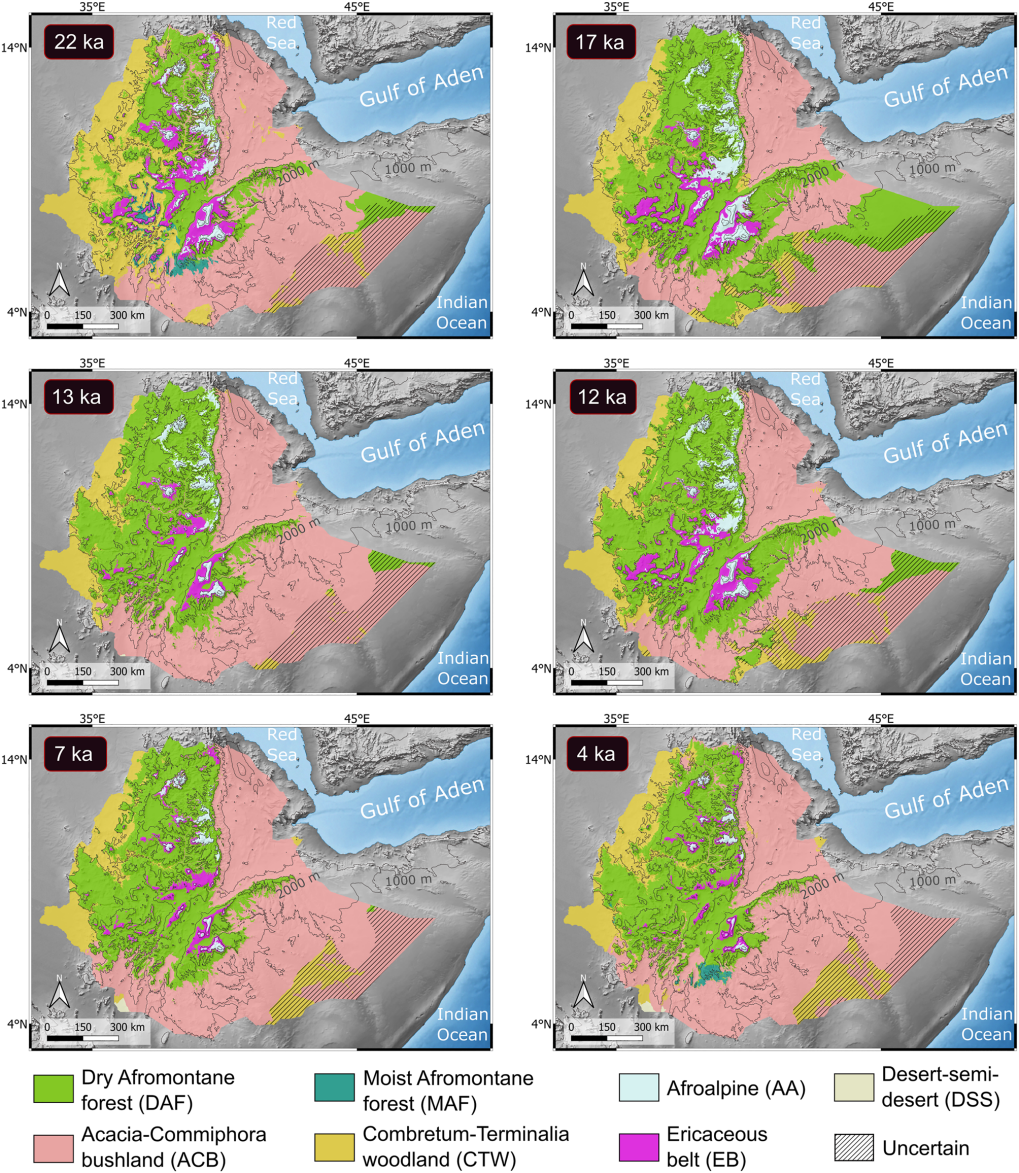
Modelled distributions of Ethiopian vegetation units for six key time slices: peak of the Last Glacial Maximum (22 ka), Late Glacial (17 ka), Late Glacial interstadial (13 ka), Younger Dryas (12 ka), African Humid Period (7 ka), and late Holocene aridification (4 ka). The extent of Afromontane forests is estimated to have attained a maximum during Late Glacial. The maps were produced using projections of the vegetation unit’s models into past climatic time slices (see Methods). Additional maps of distributions for time periods every thousand years are provided in the Supporting Information. Maps created by the authors using QGIS v3.16.16 (URL: http://qgis.org).

We have produced maps of potential vegetation distribution for 22 past time slices, one every thousand years since the LGM, at 1 km spatial resolution (Supplementary Figs. S2.1–S.2.22). The area under the receiver operating characteristic curve (AUC) values range between 0.71 and 0.96 for all the models, indicating a good to excellent model performance^31,32^. AUC values are negatively correlated with the size of the area of the VU modelled. Thus, VUs with relatively smaller areas like AA, EB and MAF display the highest AUC values (> 0.9; Supplementary Table S1). The maps time series shows significant changes in the distribution of the major VUs of Ethiopia through time. Six key time slices are selected for Fig. 2.

According to our simulations, during the LGM (22–20 ka), those VUs adapted to cold conditions (AA and EB) covered an area considerably larger than at present (ca. 100,000 km^2^ vs. ca. 18,000 km^2^ today), which extended into lower elevations and to the mountains located to the north and west of their current area (Fig. 2). The estimates derived from the models suggest that the lower limits of AA and EB laid at altitudes slightly below 2600 and 2300 m, respectively, in the Arussi and Bale mountains, which means 600–700 m below their current limits. Since the LGM, EB is envisaged to have reduced its area gradually up until the relatively warmer Late Glacial interstadial (13 ka; Supplementary Figs. S2.1–S2.10). However, the large space occupied by AA in the highlands remained more stable during the same period, showing higher resilience.

The predicted large areas with suitable conditions for the development of DAF indicate a significant extent of Afromontane forests during the LGM (Fig. 2). Unlike AA and EB, the large area covered by DAF is somewhat unexpected, as it has often been assumed that cold and dry conditions, which are characteristic of the LGM, led to the spread of open savannas, grasslands and semi-desert landscapes in the Horn of Africa, including the Ethiopian highlands^33–36^. The simulated extent of DAF attained its maximum at 17–16 ka boosted by low temperatures, and slightly higher precipitations and CO_2_ concentrations than during the peak of the LGM^24,37^. DAF extent remained substantial for most of the remaining Late Glacial, only retreating slightly during the interstadial warming (Bølling-Allerød period), notably at 13 ka.

For MAF, the estimates indicate that the areas with highest climatic suitability during the LGM were in the mountains of the south-western part of the country where high rainfall occurred. The main contributor to the distribution of MAF is mean annual precipitation (Supplementary Table S2). However, given that the temporal variations of this variable in the highlands remained mostly within a range that is suitable for MAF, its predicted distribution is mainly dependent on variations of the precipitation of the driest month. After the peak of the LGM (22–20 ka), the precipitation of the driest month dropped below the critical threshold (ca. 10 mm) necessary to sustain MAF.

Because the ecotone between the forested VUs and those dominated by savannas (ACB and CTW) lies at the lower limit of the forests, past expansions of DAF were associated with the retreat of ACB and CTW. Overall, both VUs maintained areas comparable to present over the LGM and smaller than at present during the subsequent Late Glacial millennia.

The onset of the Younger Dryas (YD), a climatic cooling event represented by the 12-ka simulation (Fig. 2), induced a new expansion of AA and EB within the highlands and a downhill shift of DAF at mid altitudes. After the YD, the regional effect of global warming that characterized the beginning of the Holocene (from ca. 11 ka onwards) led to a gradual decrease of those VUs adapted to colder climate and an uphill shift of the forest-savanna ecotone. The effect of the African Humid Period (AHP), which had its peak roughly between 11 and 7 ka^38^, did not change this overall pattern significantly. It seems that higher precipitation in the study area during the AHP only slowed down the steady DAF decline, a process that intensified after the termination of the AHP at 5 ka throughout the late Holocene aridification. The attenuated effect of the AHP in East Africa as compared to the northern part of the continent has been pointed out in several studies^39–42^. Interestingly, MAF started recovering at ca. 5 ka, coinciding with the end of the AHP. MAF recovery is estimated to have originated from refugia located at mid-altitudes (1900–2400 m) in the southern sector of the Bale Mountains, to the east of Chamo and Abaya lakes (Supplementary Fig. S2.18). Despite an overall regionally drier climate and lower mean annual precipitation after the AHP, the simulations reflect that rainfall was more evenly distributed throughout the year and current drought-free conditions developed in the south-western highlands.

The predicted distribution of desert and semidesert landscapes is negligible for most of the time span analyzed. However, it is possible that the areas located in the lowlands of the south-easternmost part of Ethiopia, featured with a dashed pattern in the maps, comprised sparse desert vegetation or were devoid of vegetation. The models consistently return low probability values (< 0.1) of occurrence for all VUs in these areas, suggesting that this region underwent climatic conditions that were not conducive for the development of any of the VUs analyzed.

## Drivers of Afromontane forest expansion and regression

One of the most remarkable findings of the simulated vegetation distributions is the observed phases of expansion and regression of Afromontane forests. These are in contrast to the long-standing hypothesis that savanna and grasslands became more predominant in the Horn of Africa during the LGM^33–36^. Our reconstructions show that the interplay between the West Atlantic and Indian Ocean monsoon activity–which controlled precipitation–and the regional effect of global climate on temperature exerted a decisive control on the habitat configuration. Furthermore, atmospheric CO_2_ concentrations must have also played a role in the distribution of habitats. The effect of CO_2_ on vegetation must have been significant during the LGM (22–18 ka), when CO_2_ levels were below 200 ppm^37^, and gradually became subordinate as CO_2_ concentrations approached pre-industrial levels. During the Holocene, CO_2_ impact on vegetation distribution is estimated to have been neglectable.

While most fossil and model evidence show that overall precipitation across East Africa was lower than at present during the LGM, the intensity of this aridity is still a matter for debate and spatial variability certainly occurred^43,44^. Paleoclimate studies have provided compelling evidence that the LGM saw stronger variability in Indian Ocean Dipole (IOD) events, resulting in a predominance of the positive IOD state^45^. Positive IOD events are linked with enhanced atmospheric convection and rainfall over East Africa, and particularly Ethiopia^46^. Additionally, it has been argued that areas of equatorial Africa close to the Indian Ocean coast were even wetter relative to present due to the combined effect of low sea level and ice–albedo feedback^47^. Northern Hemisphere cooling by ice–albedo feedback during the LGM drove a weakening of the Indian monsoon and southward movement of the ITCZ, facilitating the penetration of Indian Ocean humid air masses into East Africa^47^. The paleoclimate data used in this study are consistent with the above climate framework. The modelled climatic niches indicate that precipitation, although generally lower than at present, remained within a range suitable for the subsistence of DAF across large areas of Ethiopia (Fig. 2). The enhanced contribution to rainfall by easterly winds from the Indian Ocean during the LGM and the subsequent Late Glacial millennia is reflected in the particularly extensive downward expansion of DAF on the slopes of the eastern mountain ranges (Fig. 2, Supplementary Figs. S2.1–2.10). Within the above-described context of rainfall pattern, temperature played a key role in the observed altitudinal shifts of vegetation. Cooling at the peak of the LGM (ca. 22 ka) induced a lowering of the forest-savanna ecotone down to elevations between 1400 and 1600 m in the main Ethiopian mountain ranges. The modelled data produced with and without consideration of the CO_2_ effect on vegetation, indicate that the forest downward expansion was restrained during the LGM (22–18 ka) due to very low CO_2_ concentrations. These favored open habitats (CTW and ACB) at the expense of forests, operating as a driving factor opposed to low temperatures and, therefore, containing the forest trend to migrate toward lower elevations (Fig. 2, Supplementary Figs. S2.1–2.5). From 17 ka, CO_2_ concentrations remained above 200 ppm, allowing for a more significant lowering of the forest-savanna ecotone associated to low temperatures during the last millennia of the Late Glacial. These ecotonal regions have long been regarded as representing ideal habitats for early humans, due to their rich resource availability^48–50^. As a consequence of the ecotone lowering, the Rift Valley, which acts nowadays as a major biogeographical barrier^51,52^, was partly covered with DAF connecting large Afromontane forest areas on both sides of the valley. Further supporting evidence that cooler conditions favored the downslope migration of the Ethiopian forests derives from global biome models which, although with coarse spatial resolution, estimate a significant presence of ‘temperate broad-leaved evergreen forest’ in the eastern African mountainous areas^53^. The latter biome is interpreted to be equivalent to Afromontane forests in this region. Considering that species respond individualistically to climate^54,55^, it is most likely that only a set of species out of the diverse array that comprises DAF migrated downwards. These species would integrate with elements from more open ecosystems forming singular, mixed vegetation types. Like in the Ethiopian highlands, a downslope shift of the tree line during the LGM has been inferred for tropical mountains of Burundi^41,43^ and Malawi^56–58^. Our results show how the combined effects of temperature, precipitation and CO_2_ concentrations may have enabled the development of fairly continuous Afromontane habitat corridors connecting populations that are currently isolated over East Africa following a disjunct distribution pattern. After the Late Glacial, during the AHP (11–5 ka), the expansion of DAF gradually reversed in the Ethiopian highlands due to increasing temperatures and changes in the contribution of precipitation sourced from the Congo Basin and Indian Ocean. Increased temperature at the beginning of the Holocene led to a tree line rise, notably to the east of the Rift valley (Fig. 2). However, in the western mountain ranges, our simulations indicate that DAF regression was largely limited (Fig. 2), probably due to the buffer effect of increased rainfall driven by strengthened westerly winds sourced from the Congo Basin during the AHP. Such a higher contribution to rainfall in Ethiopia by westerly winds may have been facilitated by increased northern hemisphere insolation during the boreal summer, which was at a maximum at 11 ka. This process resulted in the strengthening of the Indian monsoon and therefore the movement of air masses toward India and away from East Africa^59–62^. Afterwards, during the second half of the Holocene (5 ka to present), further increasing temperatures and decreasing precipitation triggered a widespread intensification of DAF decline and its migration to higher elevations where it is restricted today.

## Models validation with pollen data

**Figure 3:**
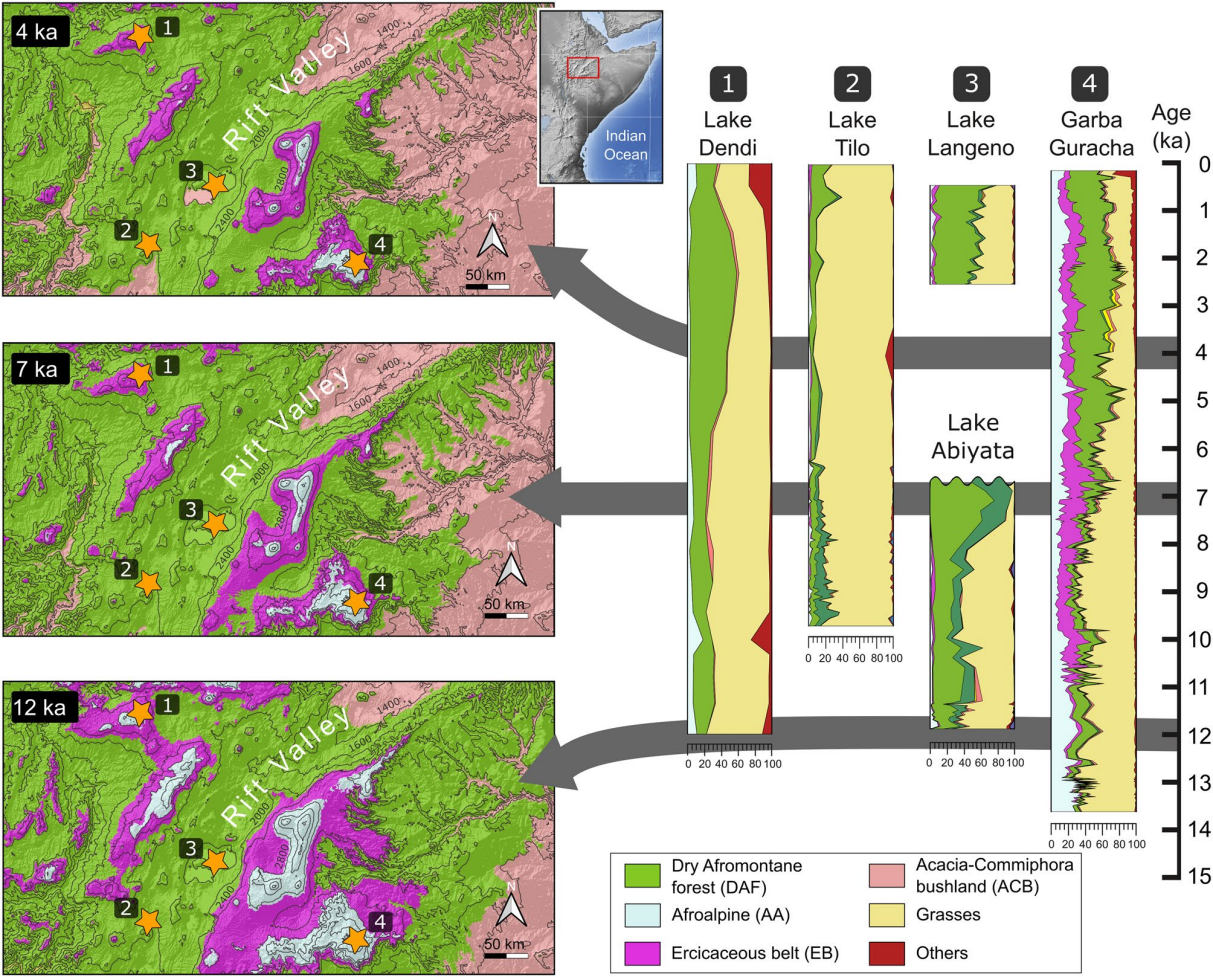
Modelled distributions of vegetation units for three time periods: Younger Dryas (12 ka), African Humid Period (7 ka) and late Holocene aridification (4 ka). Maps focus on the central Rift Valley area where the main fossil pollen records are concentrated (*Left*). Pollen data support the modelled habitat shifts. The temporal variations in the abundance of taxa follow patterns that are consistent with the distance from the depositional sites to the simulated area covered by the vegetation units (*Right*). References for pollen records: Lake Dendi^19^; Lake Tilo^17^; Lake Langeno^63^; Lake Abiyata^64^; Garba Guracha^18^. Maps created by the authors using QGIS v3.16.16 (URL: http://qgis.org).

Direct comparison of the simulated vegetation distributions with proxy data derived from regional pollen records is used here to validate the reliability of the models. The pollen sequences from Garba Guracha and the Dendi, Tilo, Langeno and Abiyata lakes are the most complete so far and reflect significant vegetation changes^17–19,63–65^ (Fig. 3). The comparisons focus on three key time slices [YD (12 ka), AHP (7 ka) and late Holocene aridification (4 ka)] because these underwent remarkable and regionally recognizable changes in vegetation that are well documented in the pollen records. The scanty fossil data that document the LGM in the highlands indicate a very high abundance of AA taxa^20,66,67^, which is in line with our simulations for high elevation areas at that time. The YD cooling period is particularly well documented in the Garba Guracha record (3950 m asl) which also contains high abundances of taxa adapted to cold conditions^18^. By that time, DAF taxa percentages increase at sites on lower elevations in the Rift Valley, being maximal at Lake Abiyata (1578 m asl). This is consistent with the predictions of high probability of suitable conditions for DAF in the Rift Valley (Fig. 3, 12 ka). During the AHP, the major vegetation changes reflected in the pollen records involve considerable increases in EB taxa in Garba Guracha, and DAF taxa in Lake Abiyata. At first glance, the increasing abundances of EB taxa seem to contradict the models for the AHP which, in contrast, estimate a reduction in the area covered by EB compared to the YD (Fig. 3, 7 ka). However, a significant upslope shift of EB from 2700 m to up to 3500 m is simultaneously modelled in the vicinity of Garba Guracha. Therefore, it is interpreted that this vertical shift accounts for the increase in EB observed on the pollen record. Thus, our simulations provide a new perspective to interpret fossil pollen data, indicating that proximity to the depositional site was the main reason why higher EB taxa abundances are recorded, rather than an expansion of EB in the highlands. Meanwhile, the highest abundances of DAF taxa documented in the Rift Valley (Lake Abiyata) confirm the predicted persistence of DAF at lower elevations. The aridification since 5 ka is characterized by increases in the percentages of DAF taxa in the pollen records from high altitudes (Fig. 3, 4 ka). Here, model-proxy agreement is also strong, with simulations showing an uphill migration of the tree line resulting in a higher potential for DAF pollen to be transported into Lake Dendi and Garba Guracha. At the same time slice (4 ka), the model still indicates high probability of occurrence of DAF in the Rift Valley, although patches of open vegetation (ACB) begin to progress, marking a change in the vegetation pattern toward the expansion of grasslands that dominate the valley today. This suggests that the areas modelled as DAF in the Rift Valley during this period were probably close to the forest-savanna ecotone. Overall, the above observations provide a good level of confidence in the simulated distributions of Ethiopian VUs. These results can be used to make inferences about human adaptations to past environmental changes in regions of Ethiopia where no other proxy data is available, and can in turn be further tested for consistency with the acquisition of new proxy data from specific locations.

## Methods

### Selection of vegetation units and climatic data

Seven vegetation units (VUs) described for Ethiopia^22^ were selected for analysis and the reconstruction of their past distribution: Afroalpine belt (AA), Ericaceous belt (EB), Dry evergreen Afromontane forest and grassland complex (DAF), Moist evergreen Afromontane forest (MAF), Acacia-Commiphora woodland and bushland (ACB), Combretum-Terminalia woodland and wooded grassland (CTW), Desert and semi-desert scrubland (DSS). These units of potential vegetation cover 97.8% of the country’s total area. The current geographic distribution of the VUs was provided by the VECEA project team as GIS vector files^23^.

Present-day (1979–2013) climate datasets for the study area were taken from the CHELSA (climatologies at high resolution for the earth’s land surface areas) database^68^. This product provides open access, high-resolution (30 arc seconds) and bias-corrected climatic data that have been calculated incorporating orographic predictors including wind fields and valley exposition, which makes of it one of the most solid options currently available for conducting ecological niche modelling^68^, notably in mountainous areas like Ethiopia. Paleoclimate data were taken from the CHELSA TraCE21k database^24^, for consistency with present-day data and because it provides a high spatial (30 arc seconds) and temporal (centennial time slices) resolution. CHELSA TraCE21k uses a similar algorithm to CHELSA to approximate the effects of orography on precipitation as well as information on LGM ice extent validated by proxy data from Greenland ice cores^24^. Time slices were selected every 1 ka since the LGM.

To characterize the VUs climatically, we extracted the value of every pixel (grid cell) from the present-day bioclimatic grid files within the geographic bounds of each VU. The frequency of each cell value was then plotted and used as an indication of the prevalent climatic conditions in the area covered by each VU (Supplementary Fig. S1). This confirms the climatic meaningfulness of the VUs as units of analysis.

### Past vegetation distribution modelling

The areas of maximum climatic suitability for each VU were estimated through ENM using MaxEnt 3.4.4^69,70^. To train the models, we used present-day climate data and large datasets of between 1000 and 10,000 presence locations with geographic coordinates randomly selected within the bounds of each VU as sample inputs. No absence data were included in the modelling. For VUs that are adjacent to the Ethiopian borders (ACB, CTW and DSS), we took the locations from a wider area across Eastern Africa, including Kenya, Tanzania, Uganda, Malawi and Rwanda, where vegetation types equivalent to the VUs defined in Ethiopia are known to occur^23^. In doing so, we captured the maximum potential climatic ranges for the VUs and minimize the potential portion of niches that are currently unavailable. Twenty-five percent of the occurrences were used as test points for model evaluation. The results were mapped in QGIS 3.16.16^71^.

To avoid introducing redundant data into the models, we limited the number of highly correlated variables used as predictors in ENM^72^. To analyze the relationship between the 19 CHELSA bioclimatic variables, we calculated pairwise Spearman's correlation coefficients for all combinations of two raster layers in Ethiopia using the raster package in R. This led us to discard some variables to reduce collinearity, such as temperature annual range; isothermality; and mean diurnal temperature range. However, some variables with high correlation (e.g. mean annual temperature and minimum temperature of the coldest month) were kept in the modelling, since they provided complementary information that is useful to introduce into the analysis. Moreover, some variables have differing relevance as causal factors in determining vegetation distribution depending on the VU modelled. Ultimately, nine variables were used: mean annual temperature; maximum temperature of warmest month; minimum temperature of coldest month; mean temperature of wettest quarter; mean temperature of driest quarter; mean annual precipitation; precipitation of wettest month; precipitation of driest month; and precipitation of warmest quarter.

The models trained for each VU were then projected into 22 different past climatic time slices (one for every thousand years since the LGM) to simulate the consequences of changing climatic conditions on vegetation distribution in Ethiopia. As a control test to ensure the validity of the projections, we conducted a projection of the models into a present-day climatic scenario. This test returned a reasonably accurate reflection of the potential natural vegetation in Ethiopia as described by Friis et al.^22^ (Supplementary Fig. S3). In total, we performed 154 projections (7 models into each of the 22 time slices).

In a further step, for each time slice a cell-by-cell analysis of the resulting suitability maps for each VU was conducted using GIS software. The cells of the grid maps with highest climatic suitability values were selected to represent the VU from which they were derived.

Besides climate, which is known to be the main factor controlling current potential vegetation distribution in Ethiopia^3,22^, atmospheric CO_2_ concentration is believed to have had a significant effect on vegetation cover during the LGM, notably in the tropics^73–76^. Low CO_2_ levels during the LGM (ca. 180 ppm) must have had an adverse physiological impact on forested habitats and a positive effect on C4 plant-dominated biomes like savannas. Various studies have shown that forest cover during the LGM is overestimated by models that only consider climate^73–75,77^. In order to assess the impact of CO_2_ on the Ethiopian VUs, correction factors were incorporated into the models. These were calculated based on the results shown in Woillez et al.^77^, who ran dynamical global vegetation models for the LGM using LGM CO_2_ levels and compared them with models produced using current CO_2_ levels. The differences in area covered by the biomes in both kind of models analyzed by Woillez et al.^77^ were translated here into correction factors by dividing the fraction cover using LGM CO_2_ levels by the fraction cover under present-day levels. These factors were applied to the probability of occurrence of each VU (Supplementary Table S3). We assume a linear relation between CO_2_ concentration and intensity of the physiological effect on vegetation growth based on Izumi and Lezine^76^. This allows us to calculate the correction factor for each time slice with a given past CO_2_ concentration. Past CO_2_ concentrations have been taken from the estimates in Yu et al.^37^ (Supplementary Table S3).

### Compilation of pollen records

We carried out a comprehensive compilation of fossil pollen records from Ethiopia to use them as validation tools for the models (Supplementary Fig. S4, Table S4). Fourteen palynological datasets were obtained from the African Pollen Database (https://africanpollendatabase.ipsl.fr), Pangaea (https://www.pangaea.de/) and Neotoma (https://www.neotomadb.org/) databases, all of them available in the public domain. These sources also provided chronologies of the sequences including recently updated age-depth models. For visualization purposes and to facilitate comparison and correlation of vegetation patterns, pollen sequences were plotted against age.

The taxa identified in fossil pollen studies were grouped according to their ecological preference (Supplementary Table S5). Out of the 361 taxa identified in Ethiopia, 240 with clear ecological significance were assigned to one of the VUs analyzed. Taxa characteristic of more than one VU or with unclear ecological significance were excluded from the assignment. Likewise, for a regional scale interpretation of the data, taxa sourced from local wetlands were not considered in the ecological grouping.

## Supplementary Information

Supplementary Information.

## Data Availability

All generated maps of vegetation distribution and detailed information on the proxy data interpreted in this study are included in the Supplementary Information of this paper and are digitally stored in the ROCEEH Metadata Catalogue (ROCEEH, 2022) with open access. The model outputs and maps have also been deposited at Zenodo repository as raster and shape files and can be downloaded at https://zenodo.org/record/7182712#.ZBeNeITMI2w.
