# Publisher Correction: Cooling-induced expansions of Afromontane forests in the Horn of Africa since the Last Glacial Maximum

**DOI:** 10.1038/s41598-023-37919-y

**Published:** 2023-07-01

**Authors:** Manuel Casas-Gallego, Karen Hahn, Katharina Neumann, Sebsebe Demissew, Marco Schmidt, Stéphanie C. Bodin, Angela A. Bruch

**Affiliations:** 1 Institute of Ecology, Diversity and Evolution, Goethe University Frankfurt, Frankfurt am Main, Germany; 2 Department of Geodynamics, Stratigraphy and Paleontology, Complutense University of Madrid, Madrid, Spain; 3 Department of Paleoanthropology, Senckenberg Research Institute, Frankfurt am Main, Germany; 4 National Herbarium of Ethiopia, Addis Ababa University, Addis Ababa, Ethiopia; 5 Palmengarten der Stadt Frankfurt am Main, Frankfurt am Main, Germany; 6 Research Centre “The role of culture in early expansions of humans” of the Heidelberg Academy of Sciences and Humanities, Senckenberg Research Institute, Frankfurt am Main, Germany

Correction to: *Scientific Reports* https://doi.org/10.1038/s41598-023-37135-8, published online 26 June 2023

In the original version of this Article, Stéphanie C. Bodin was incorrectly affiliated with ‘Department of Geodynamics, Stratigraphy and Paleontology, Complutense University of Madrid, Madrid, Spain’. The correct affiliation is listed below.

Department of Paleoanthropology, Senckenberg Research Institute, Frankfurt am Main, Germany

The original Article has been corrected.

