# Supplementary material for: Cooling-induced expansions of Afromontane forests in the Horn of Africa since the Last Glacial Maximum
Source: Sci Rep. 2023 Jun 26;13:10323. doi: 10.1038/s41598-023-37135-8 (PMC10293186; doi:10.1038/s41598-023-37135-8)
Supplement: Supplementary file 1 — Supplementary Information. [file 41598_2023_37135_MOESM1_ESM.pdf]

Supplementary Information for

Cooling-induced expansions of Afromontane forests in the Horn of Africa since the Last Glacial Maximum

Manuel Casas-Gallego, Karen Hahn, Katharina Neumann, Sebsebe Demissew, Marco Schmidt, Stéphanie C. Bodin, Angela A. Bruch

Corresponding author: Manuel Casas-Gallego
Email: manuel.casas-gallego@senckenberg.de

Supplementary Information

Figures

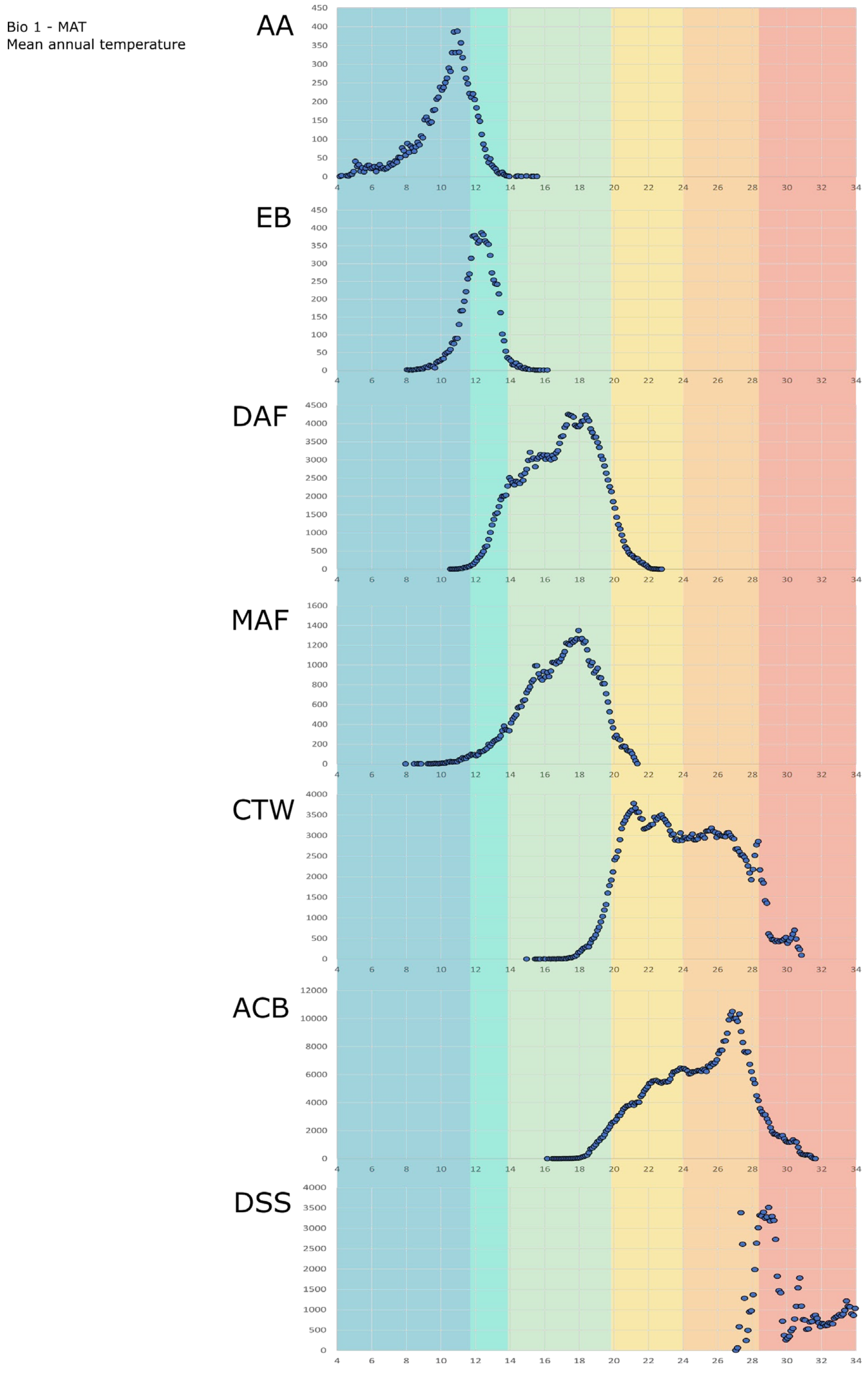

Figure S1. Frequency of mean annual temperature values of grid cells within the area of each vegetation unit. The y-axis indicates number of grid cells. The figure illustrates how the vegetation unit's climatic limits are quantified and analyzed using mean annual temperature as an example.

Figures S2.1 – S2.22. Vegetation simulations since the Last Glacial Maximum. Vegetation units are color-coded as in figures 2 and 3 of the main text. Shape files of the maps are accessible at the Zenodo repository and can be downloaded at htpp://doi.org/10.5281/zenodo.7182712. All maps were created by the authors using QGIS v3.16.16 (URL: http://qgis.org).

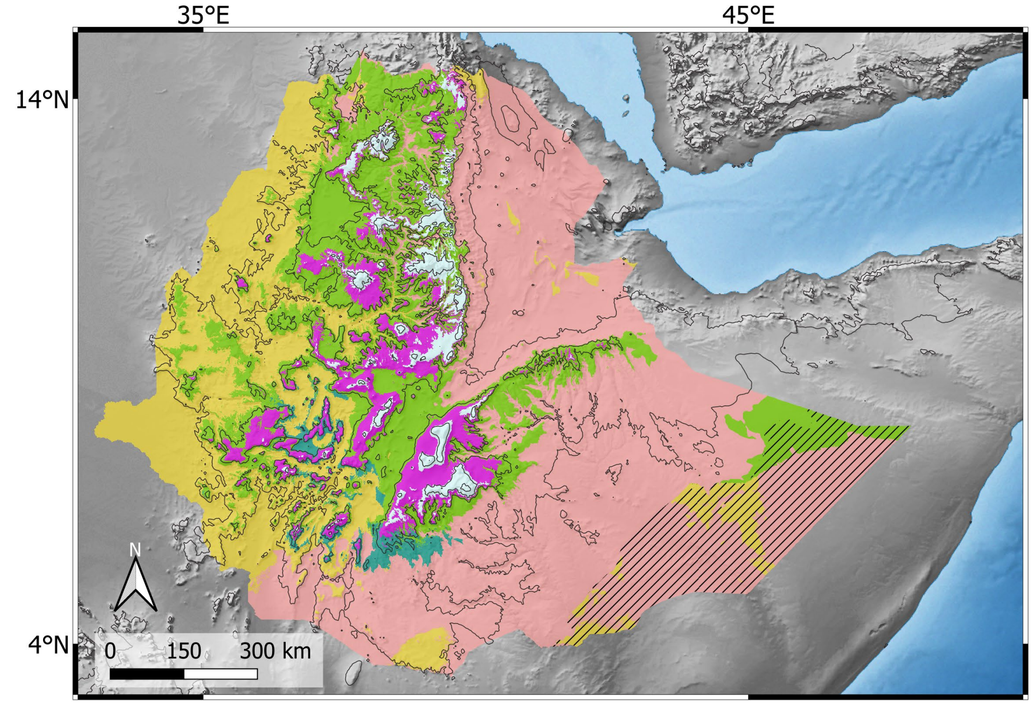

Figure S2.1a. Distribution of vegetation units in Ethiopia at 22 ka with CO_2 correction.

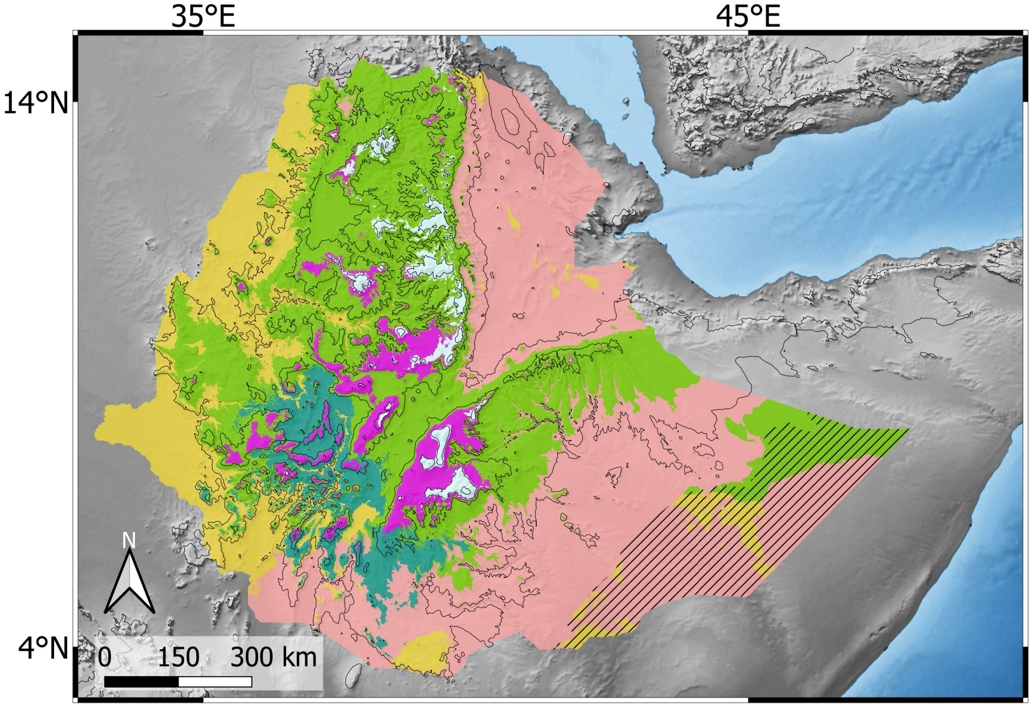

Figure S2.1b. Distribution of vegetation units in Ethiopia at 22 ka with no CO_2 correction.

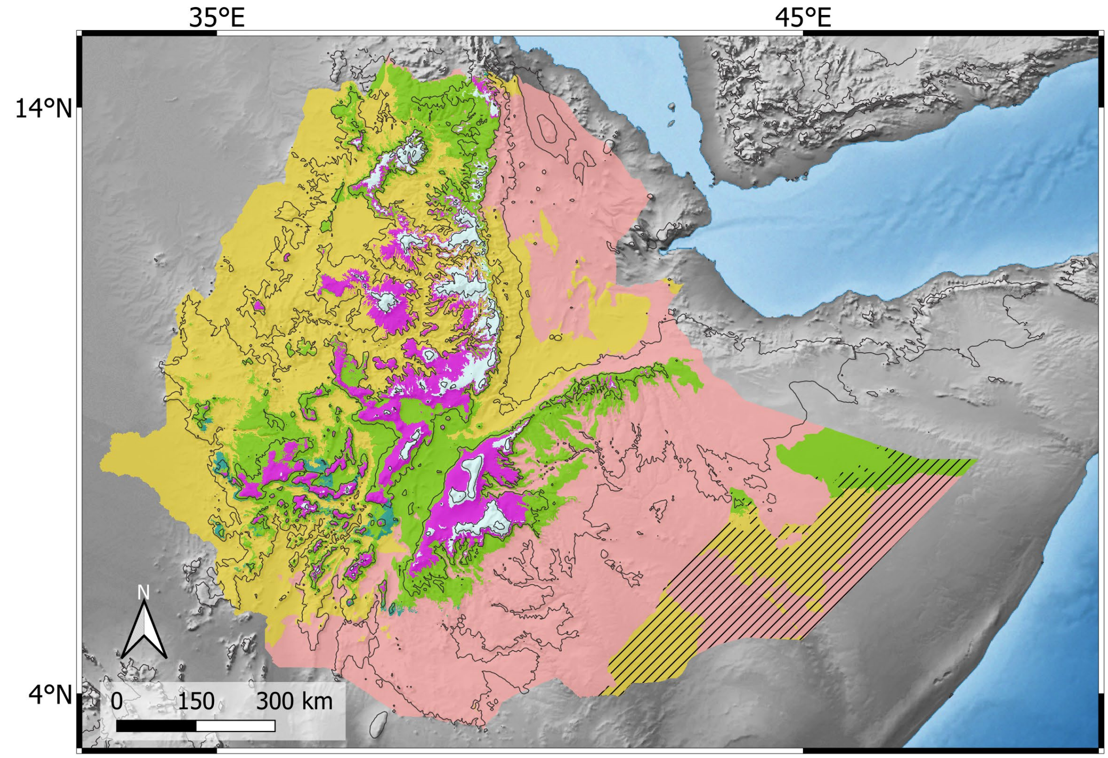

Figure S2.2a. Distribution of vegetation units in Ethiopia at 21 ka with CO_2 correction.

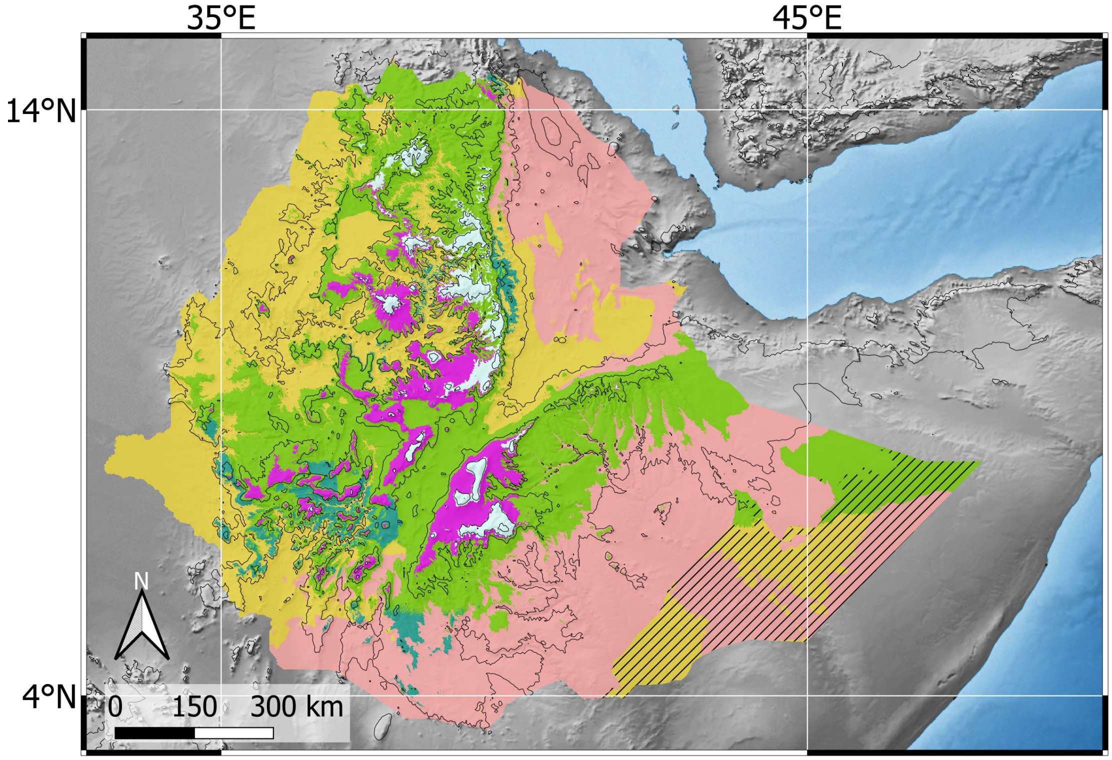

Figure S2.2b. Distribution of vegetation units in Ethiopia at 21 ka with no CO_2 correction.

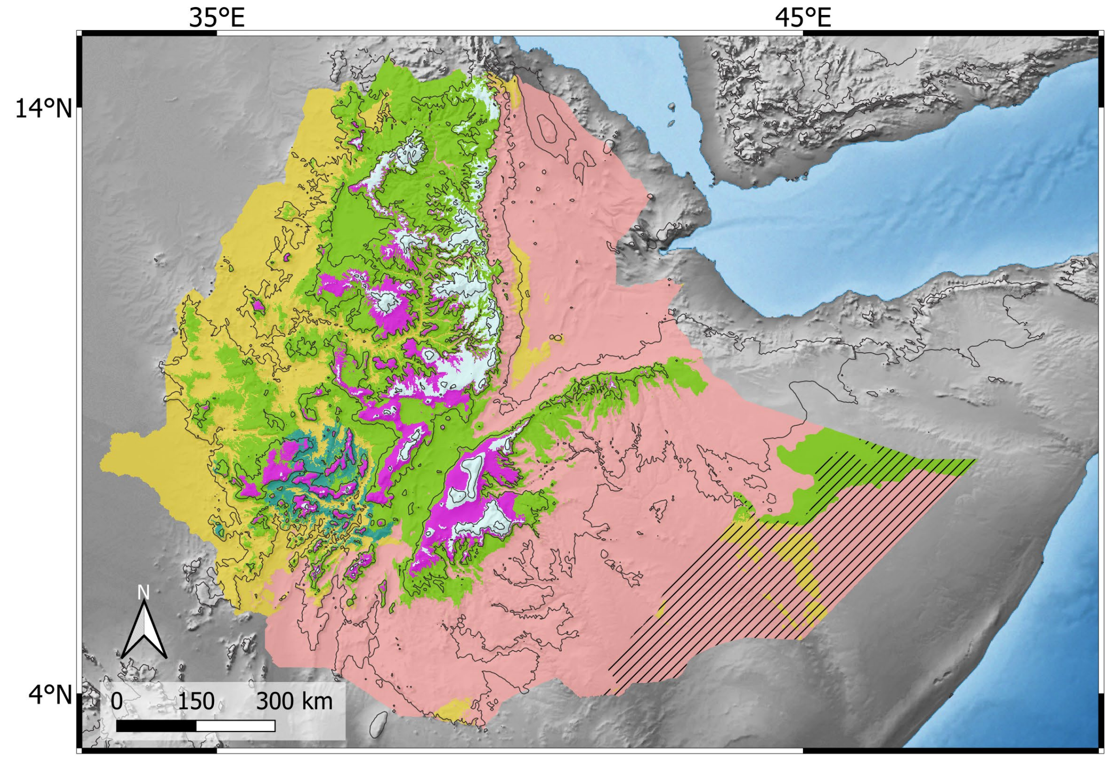

Figure S2.3a. Distribution of vegetation units in Ethiopia at 20 ka with CO_2 correction.

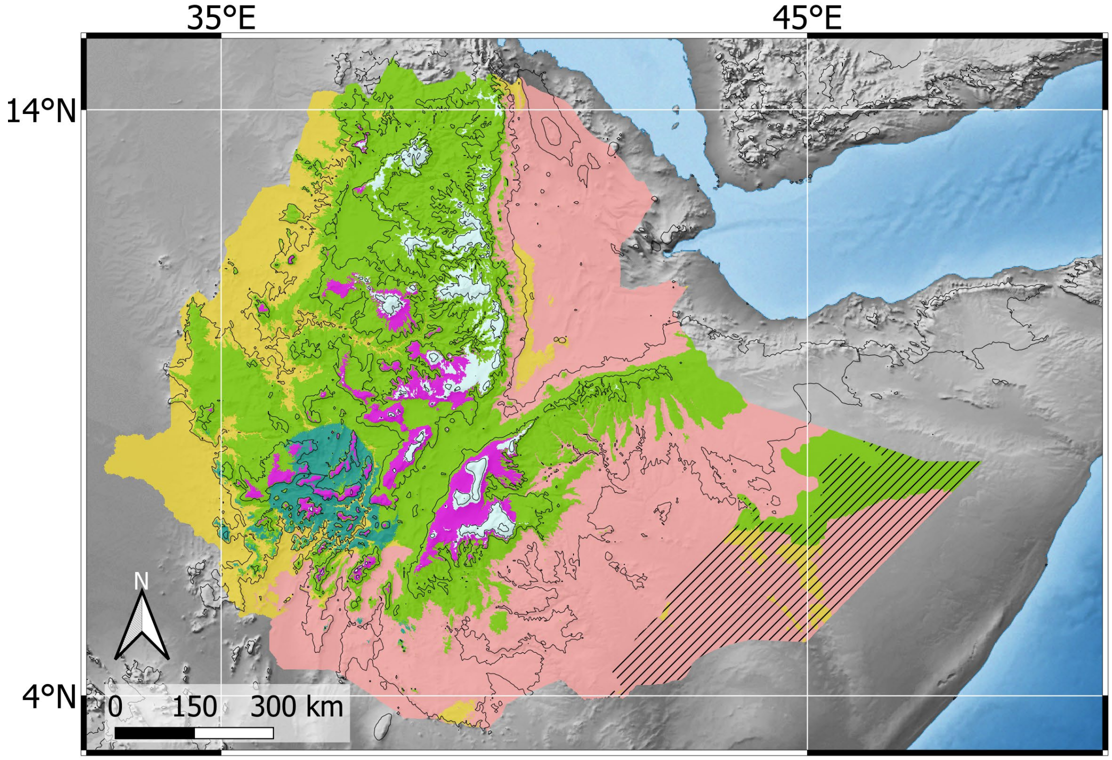

Figure S2.3b. Distribution of vegetation units in Ethiopia at 20 ka with no CO_2 correction.

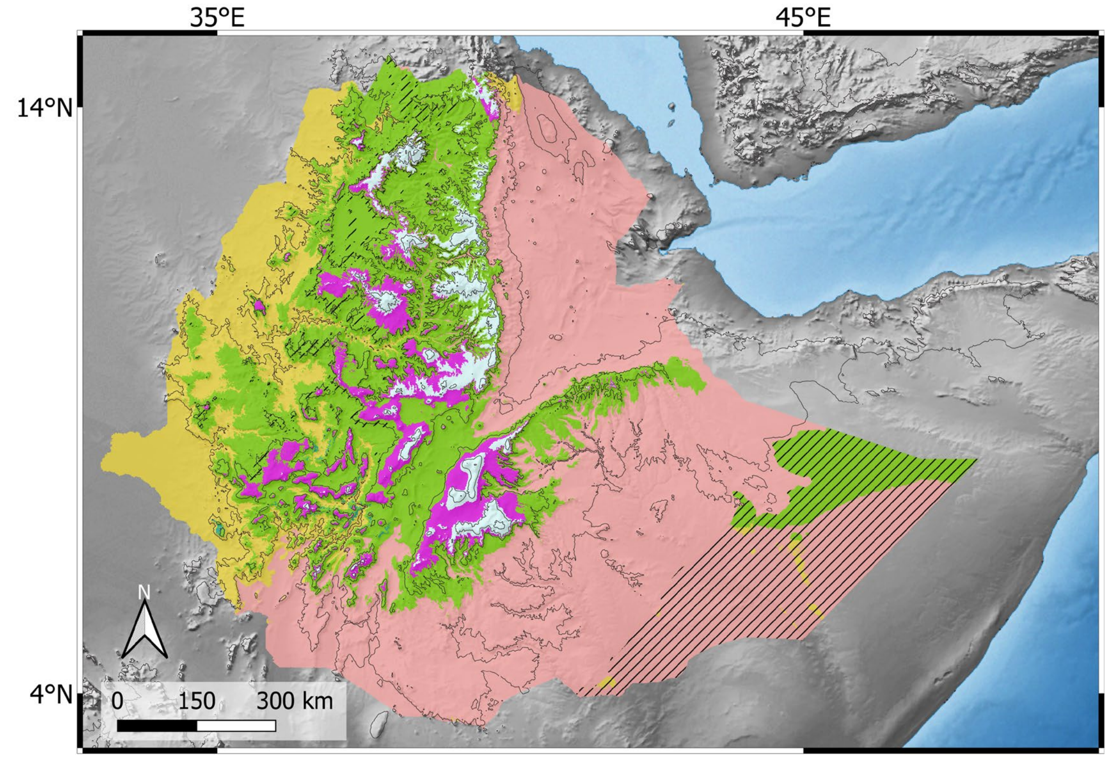

Figure S2.4a. Distribution of vegetation units in Ethiopia at 19 ka with CO_2 correction.

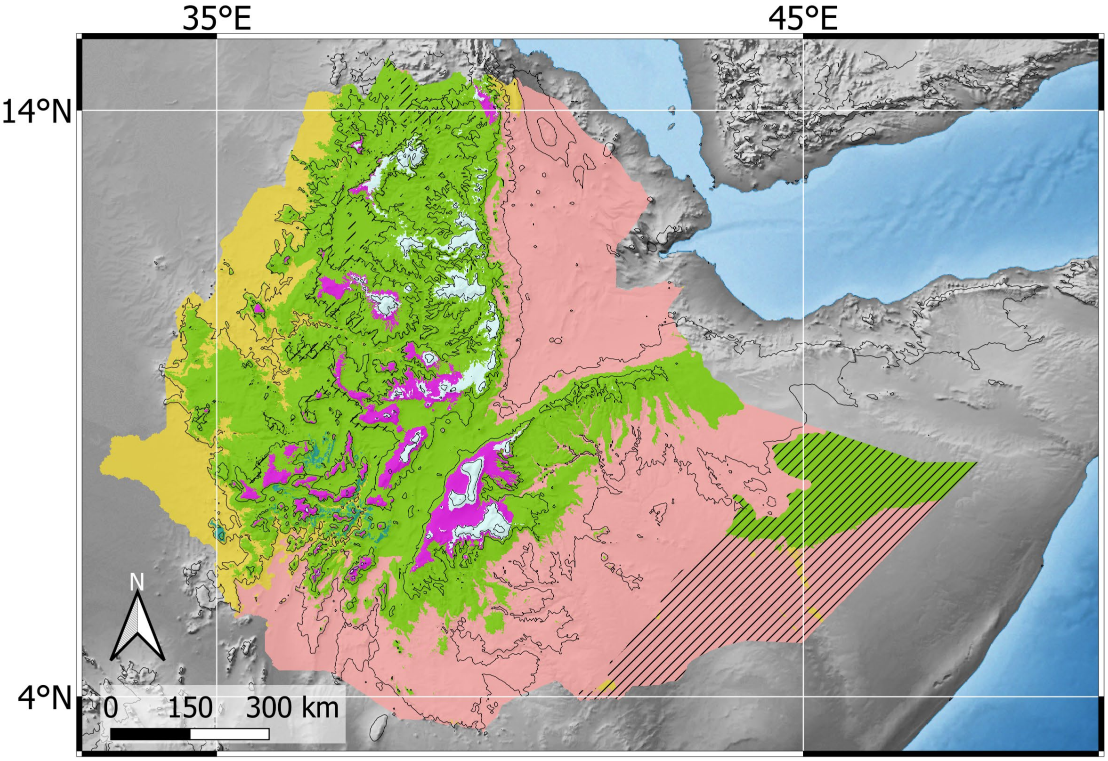

Figure S2.4b. Distribution of vegetation units in Ethiopia at 19 ka with no CO_2 correction.

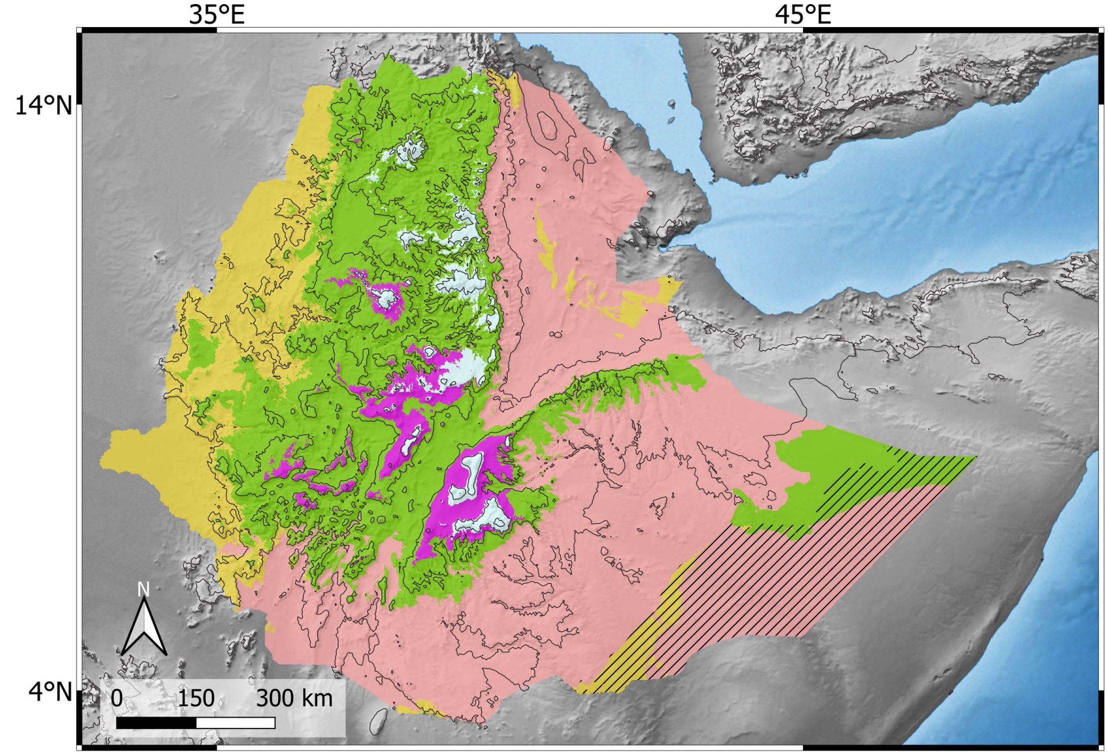

Figure S2.5a. Distribution of vegetation units in Ethiopia at 18 ka with CO_2 correction.

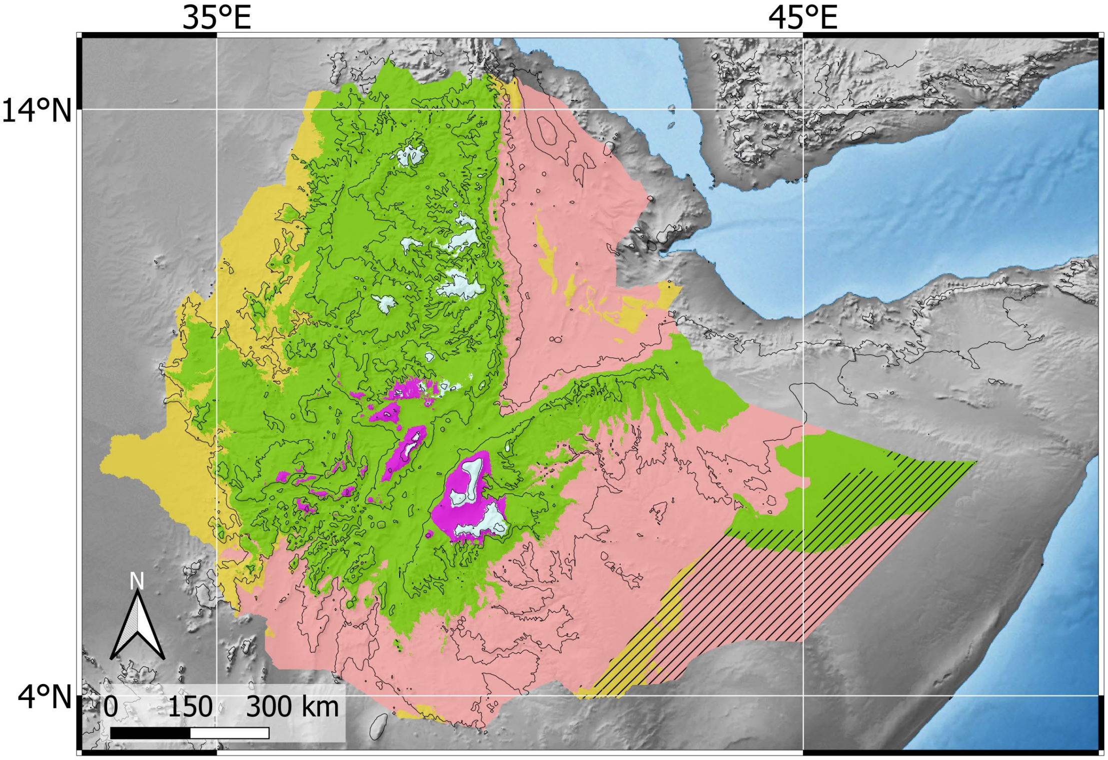

Figure S2.5b. Distribution of vegetation units in Ethiopia at 18 ka with no CO_2 correction.

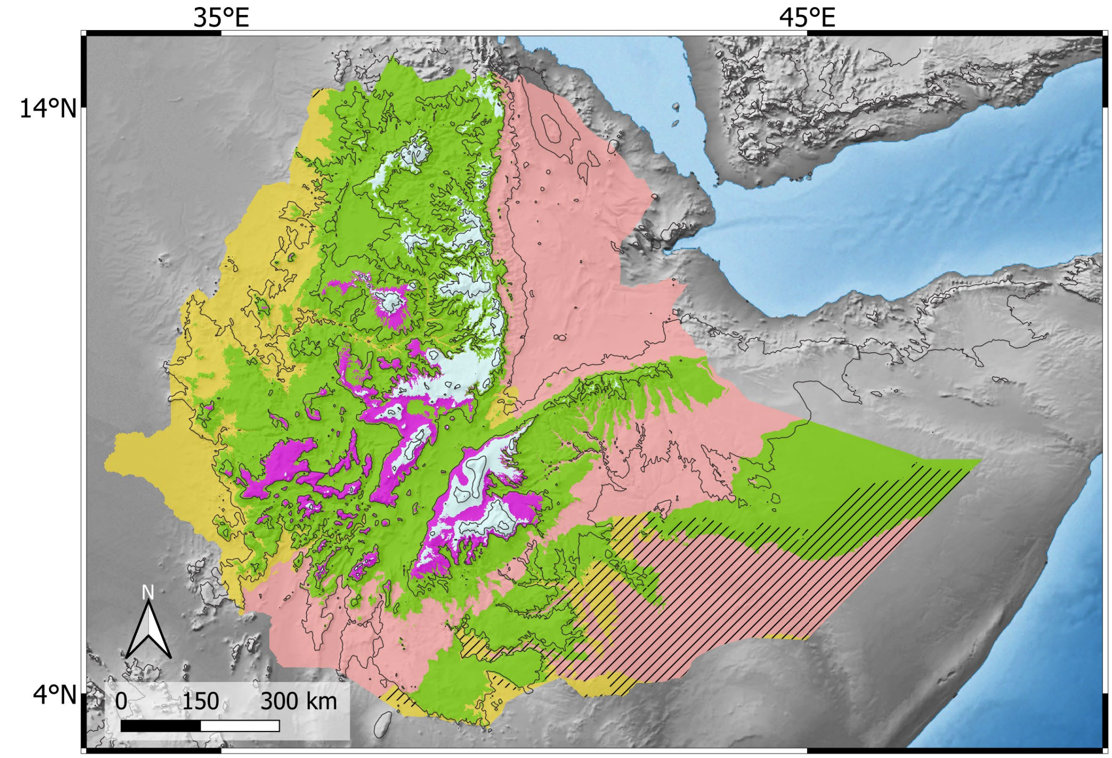

Figure S2.6a. Distribution of vegetation units in Ethiopia at 17 ka with CO_2 correction.

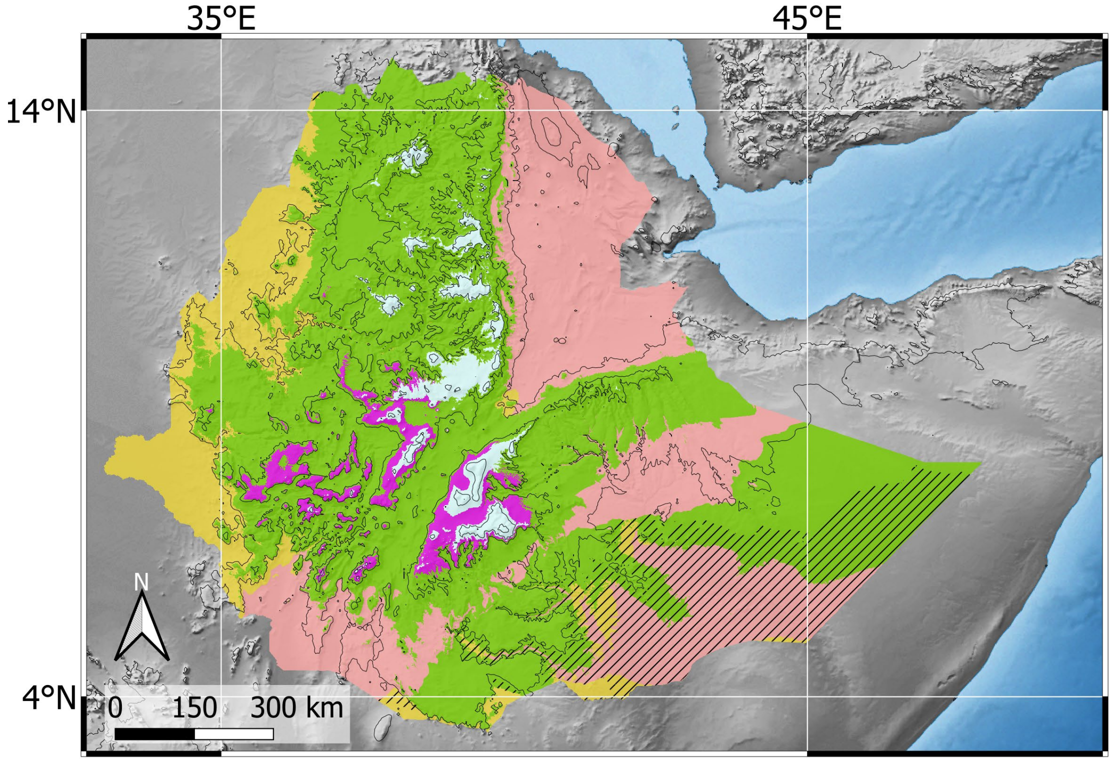

Figure S2.6b. Distribution of vegetation units in Ethiopia at 17 ka with no CO_2 correction.

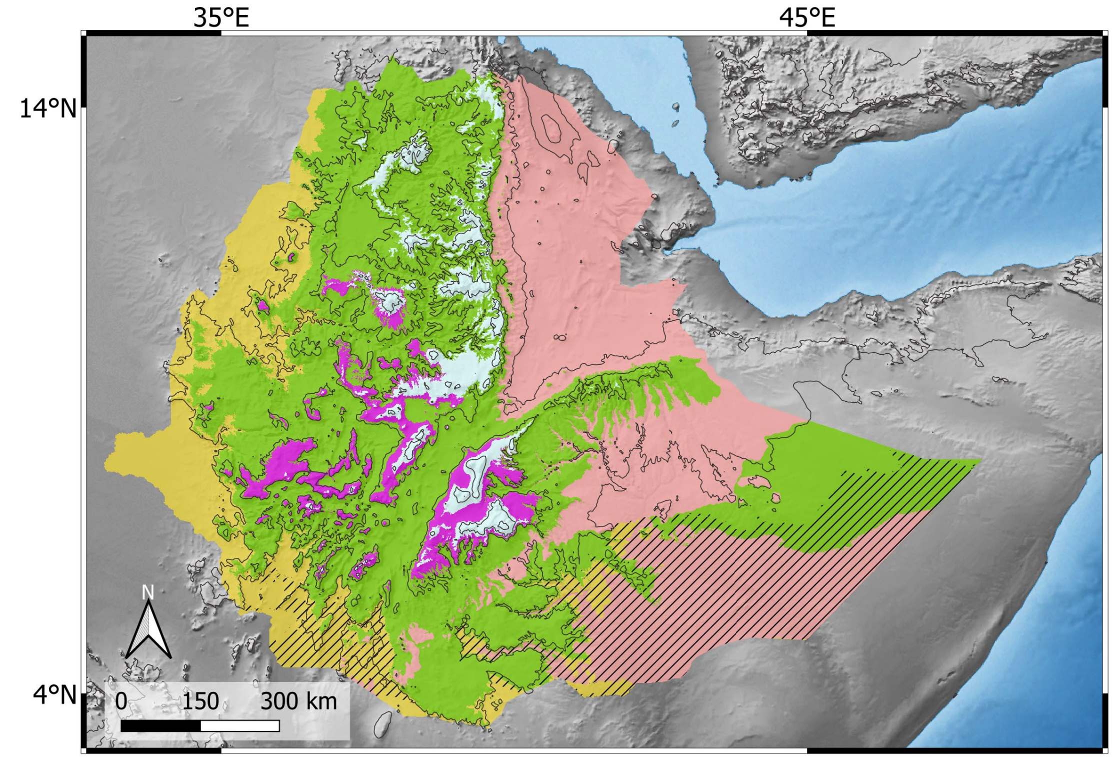

Figure S2.7a. Distribution of vegetation units in Ethiopia at 16 ka with CO_2 correction.

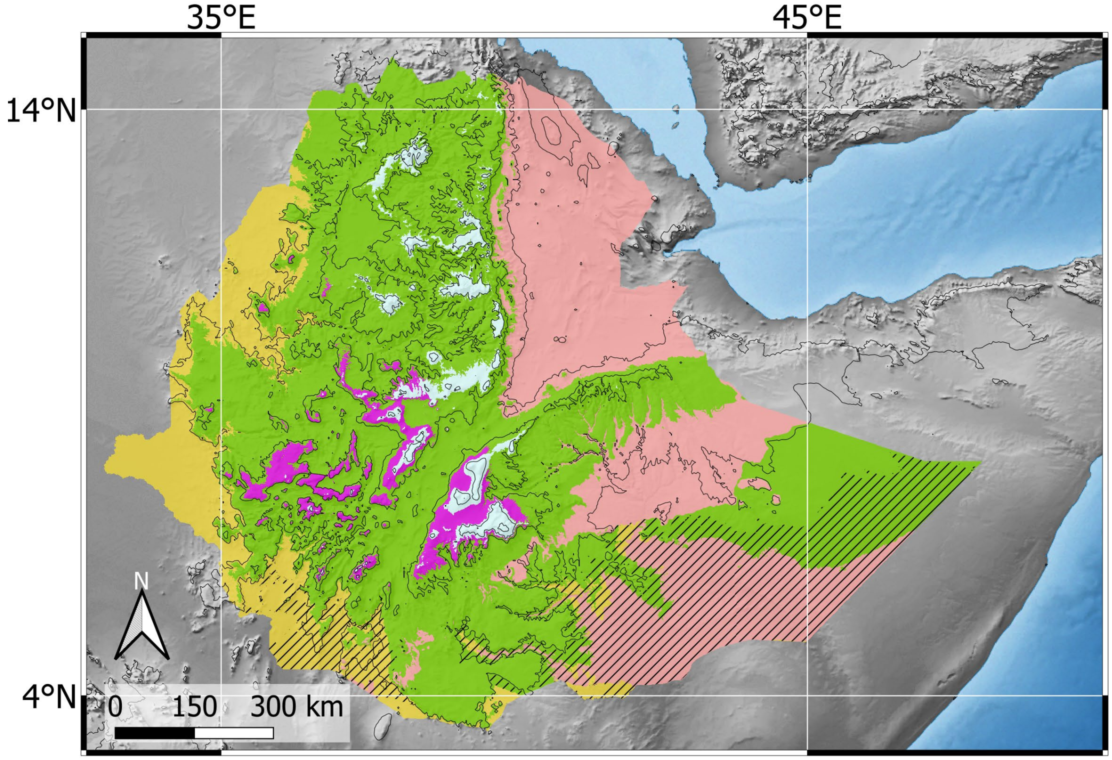

Figure S2.7b. Distribution of vegetation units in Ethiopia at 16 ka with no CO_2 correction.

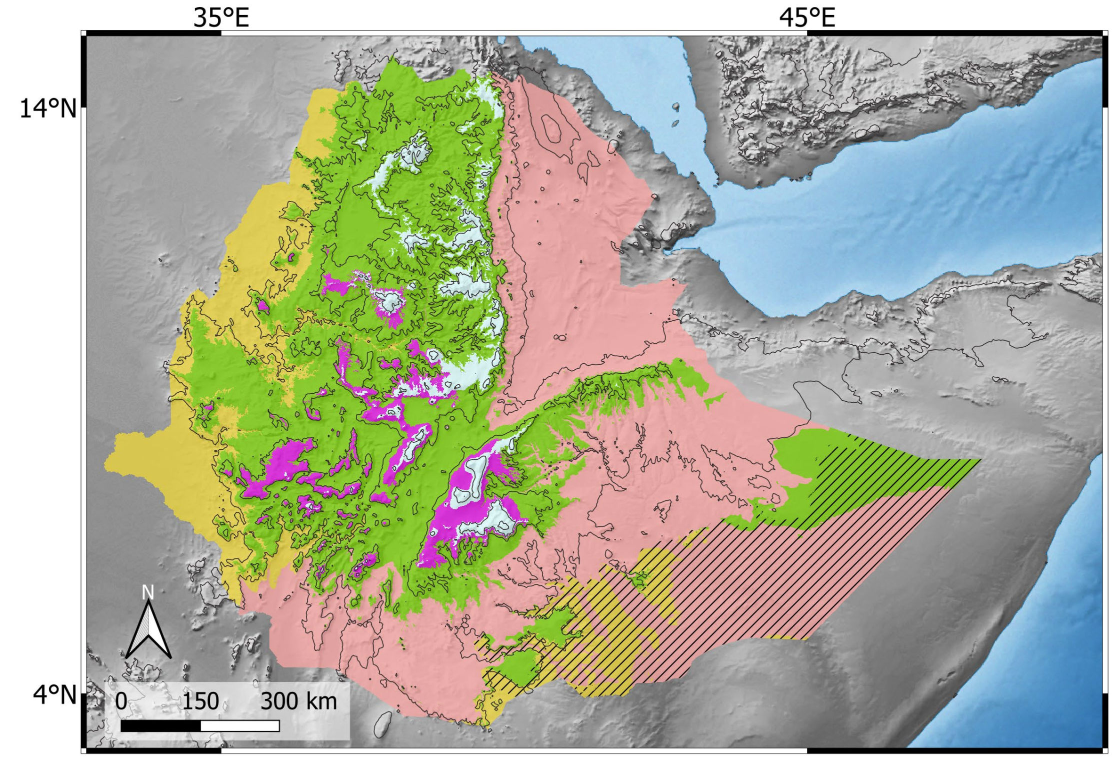

Figure S2.8a. Distribution of vegetation units in Ethiopia at 15 ka with CO_2 correction.

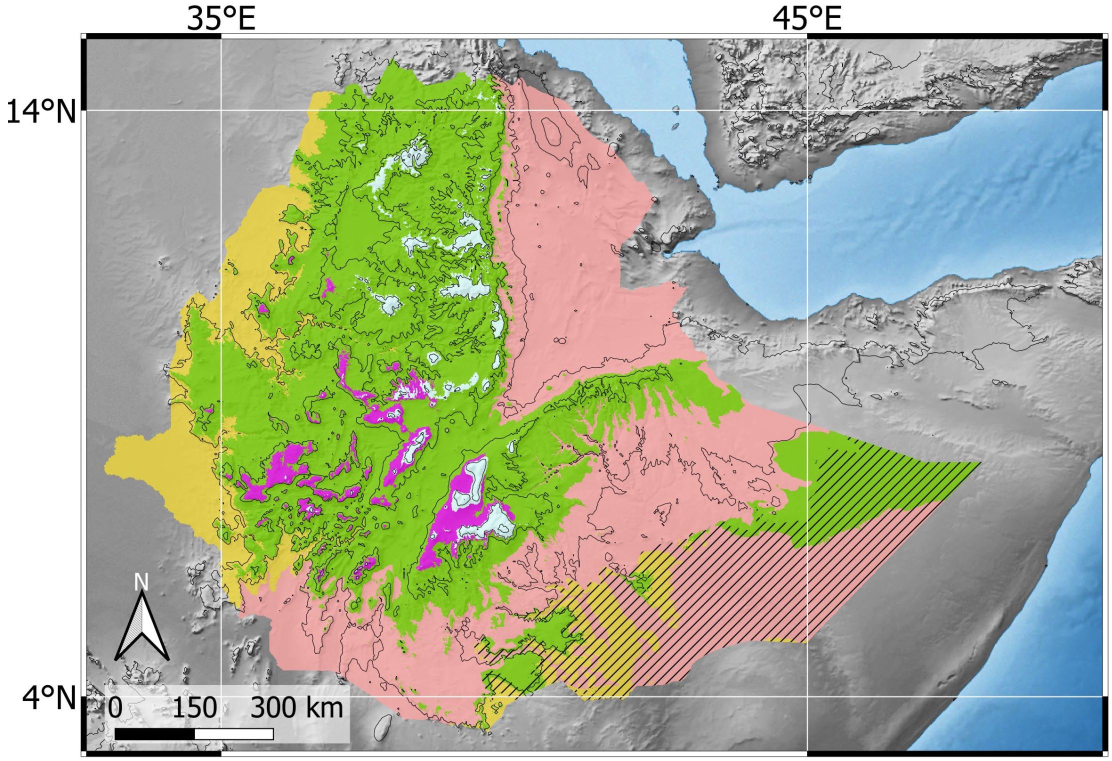

Figure S2.8b. Distribution of vegetation units in Ethiopia at 15 ka with no CO_2 correction.

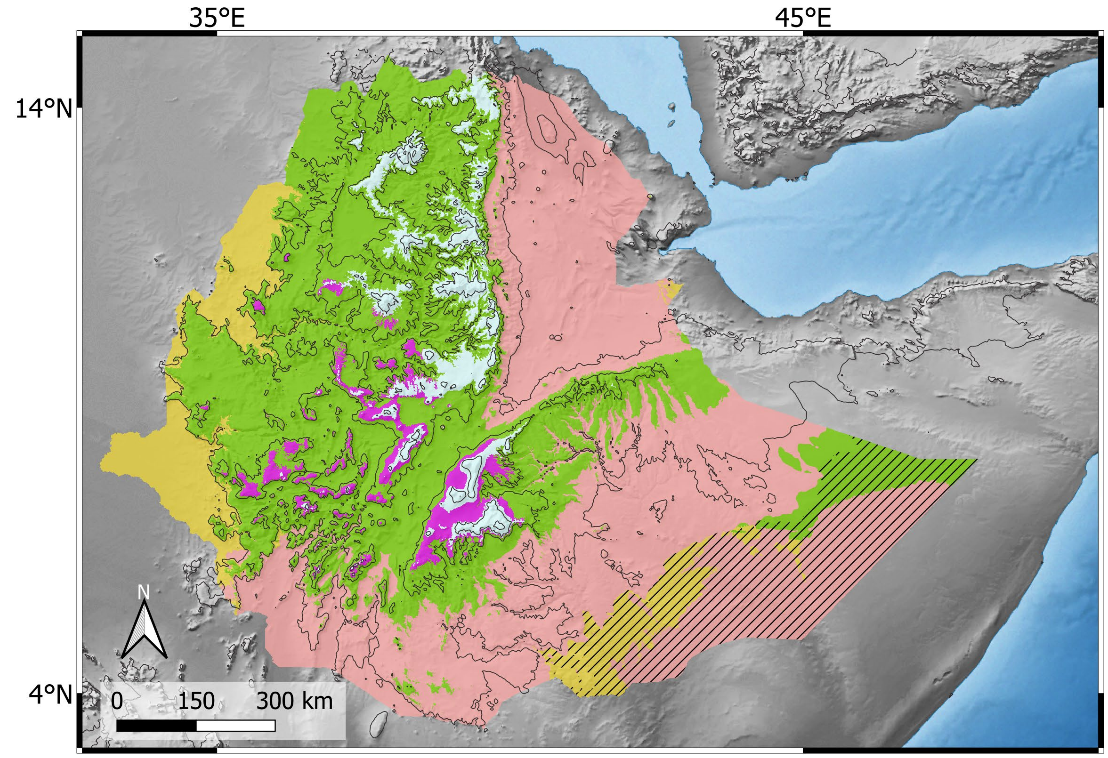

Figure S2.9a. Distribution of vegetation units in Ethiopia at 14 ka with CO_2 correction.

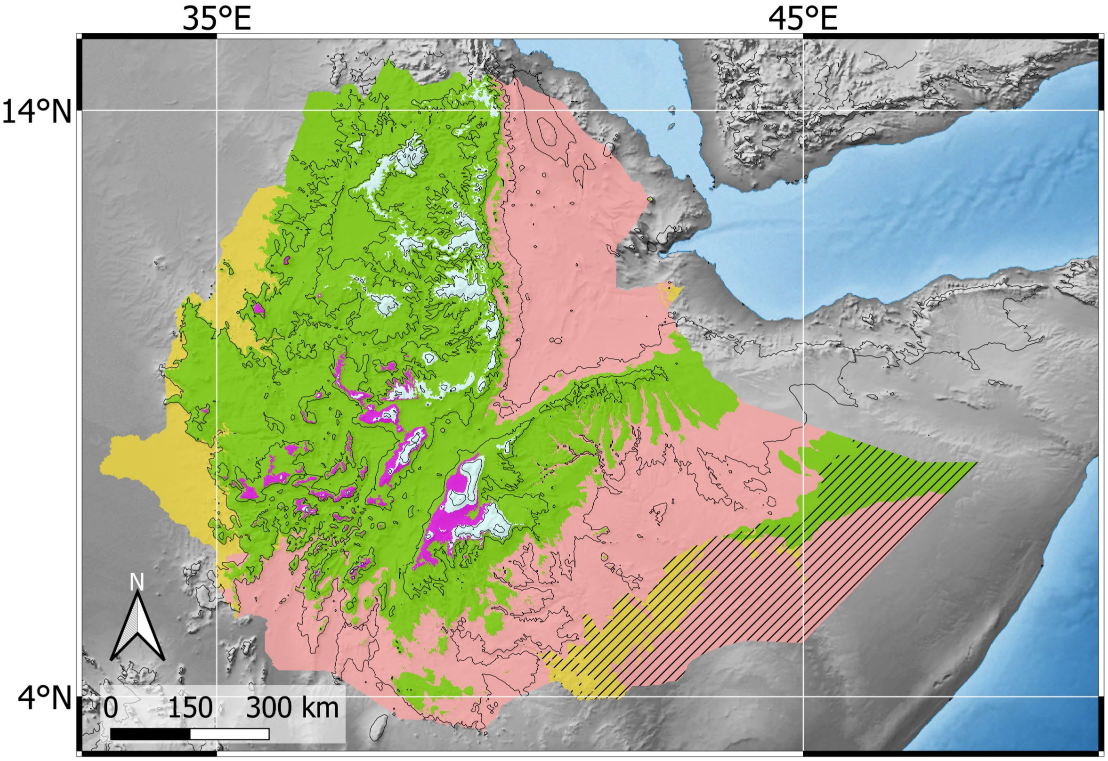

Figure S2.9b. Distribution of vegetation units in Ethiopia at 14 ka with no CO_2 correction.

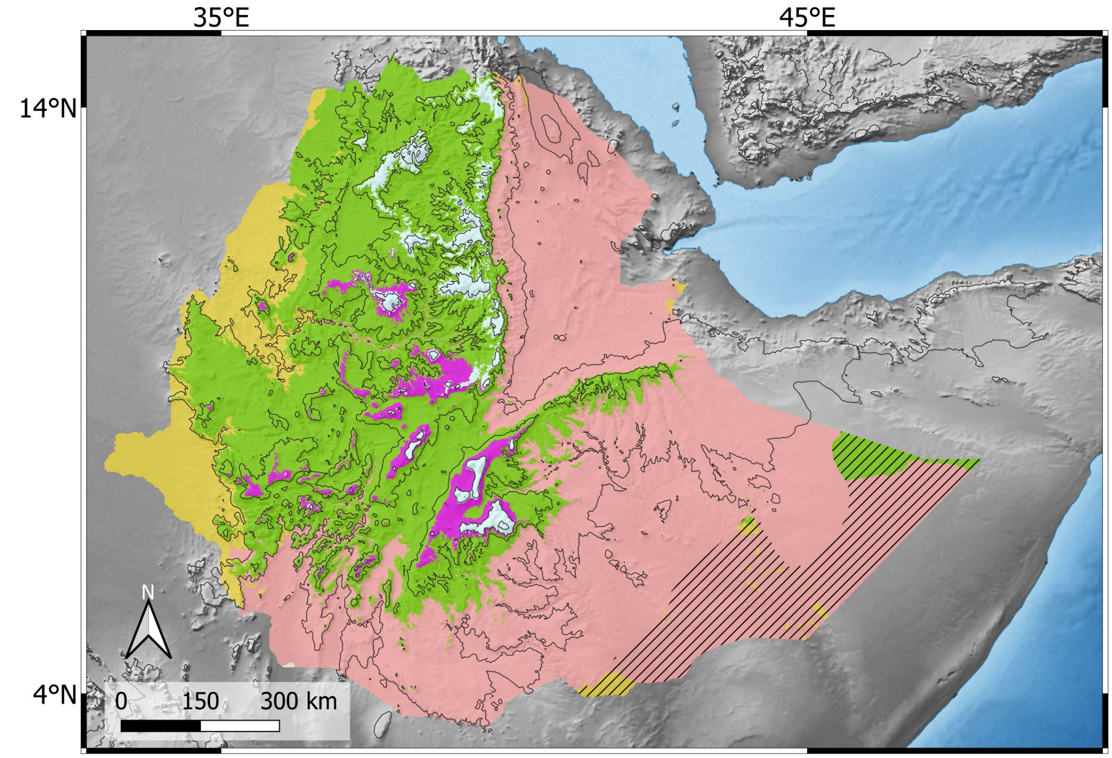

Figure S2.10a. Distribution of vegetation units in Ethiopia at 13 ka with CO_2 correction.

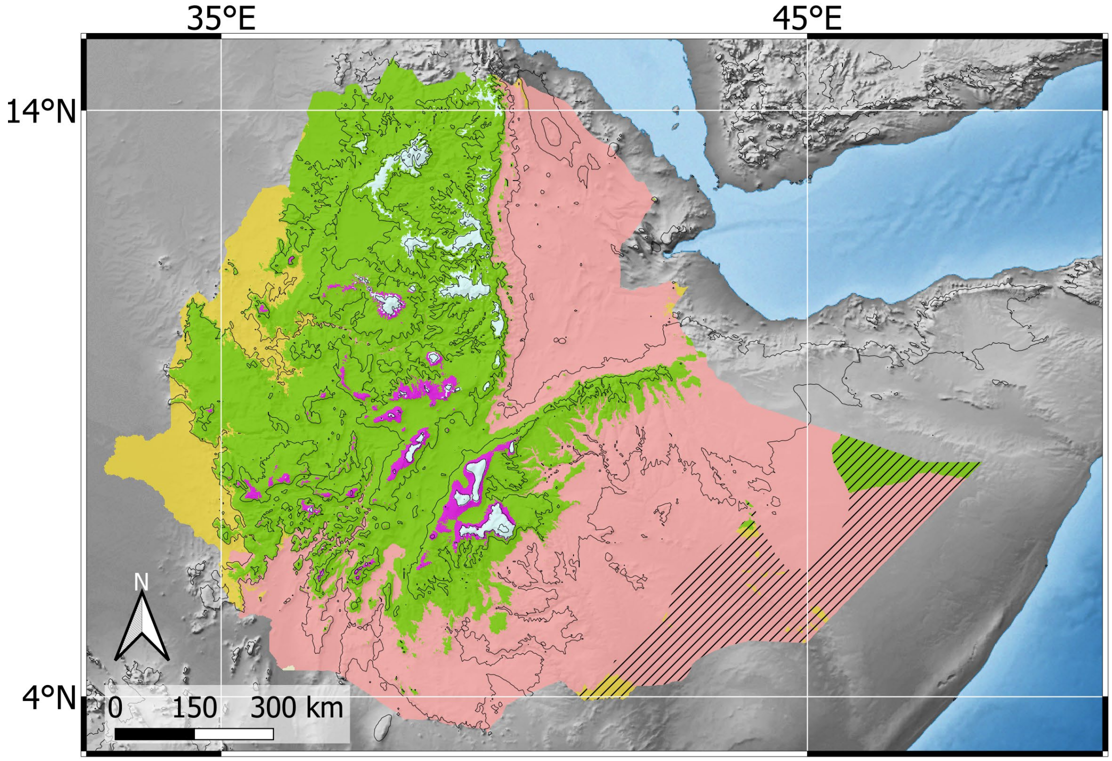

Figure S2.10b. Distribution of vegetation units in Ethiopia at 13 ka with no CO_2 correction.

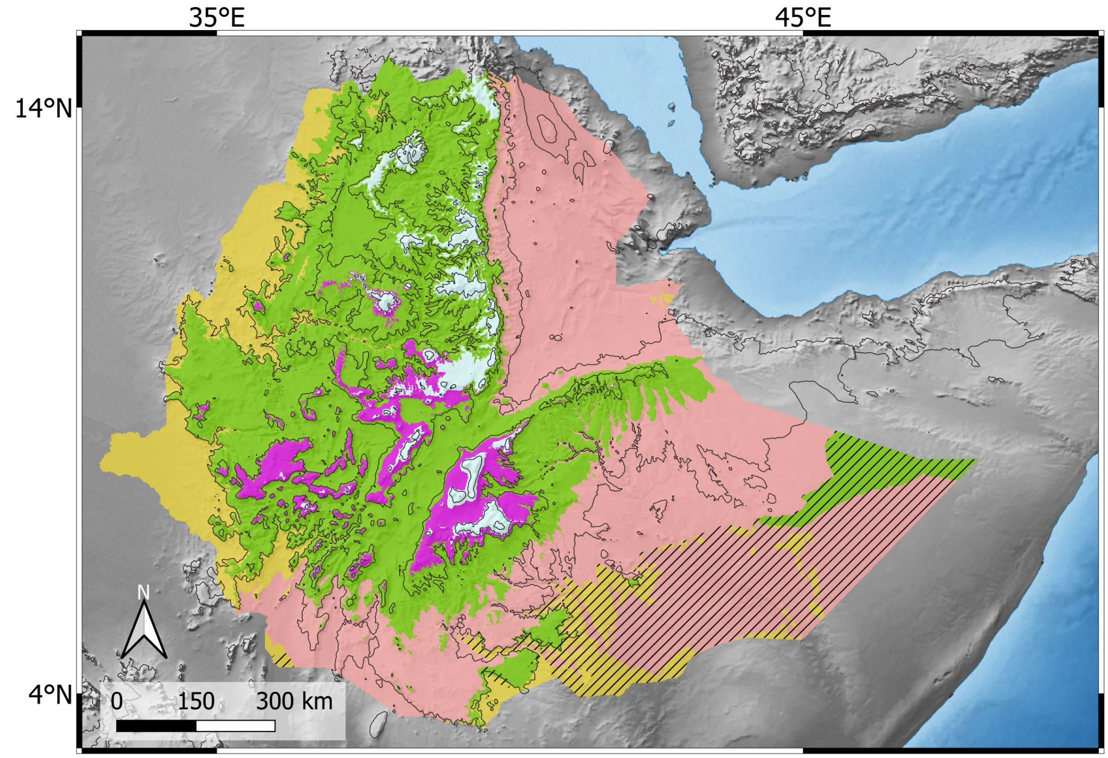

Figure S2.11a. Distribution of vegetation units in Ethiopia at 12 ka with CO_2 correction.

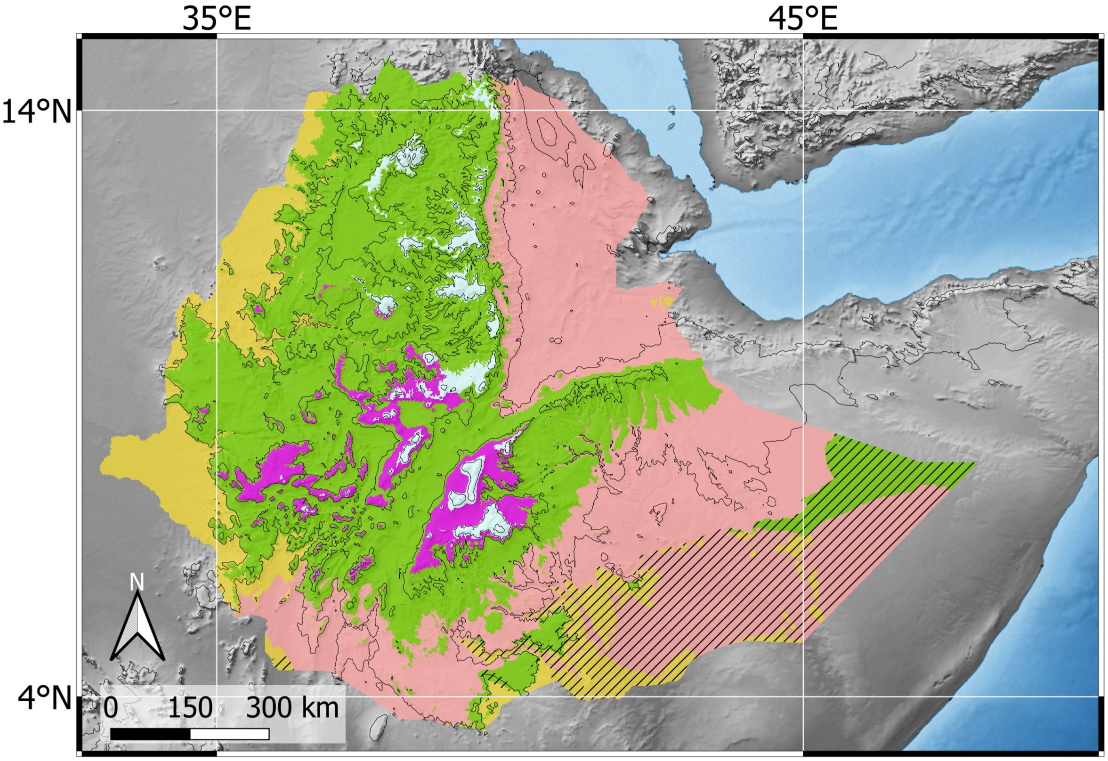

Figure S2.11b. Distribution of vegetation units in Ethiopia at 12 ka with no CO_2 correction.

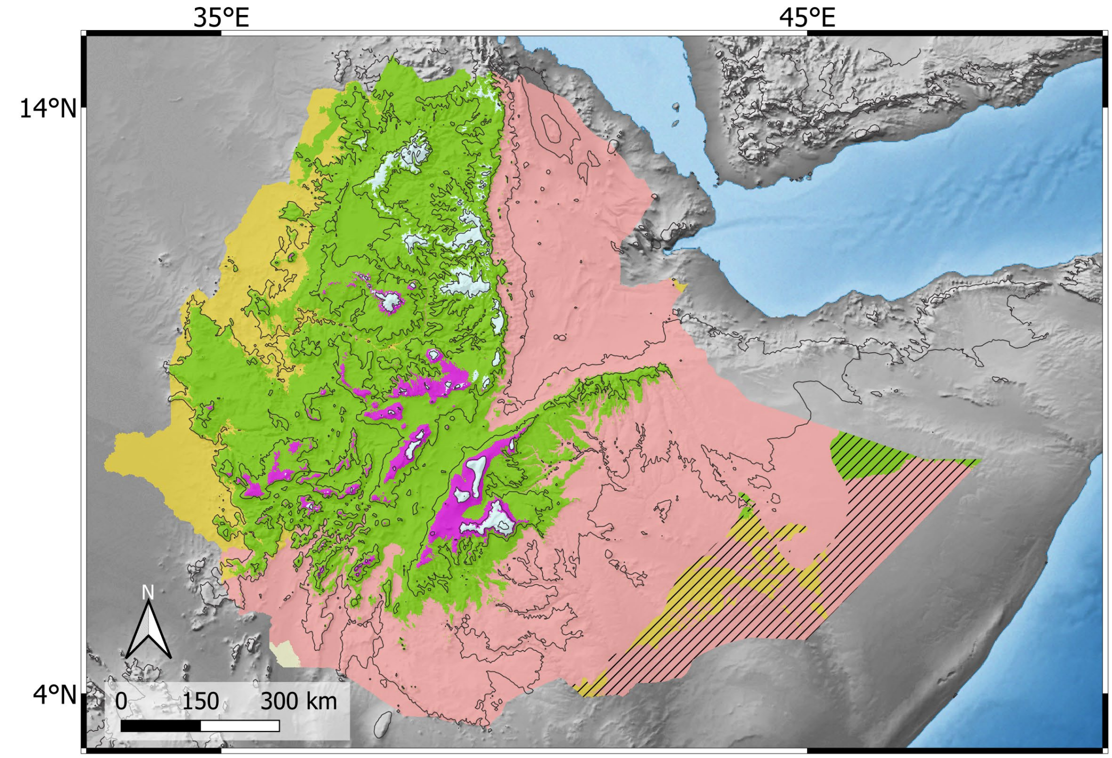

Figure S2.12a. Distribution of vegetation units in Ethiopia at 11 ka with CO_2 correction.

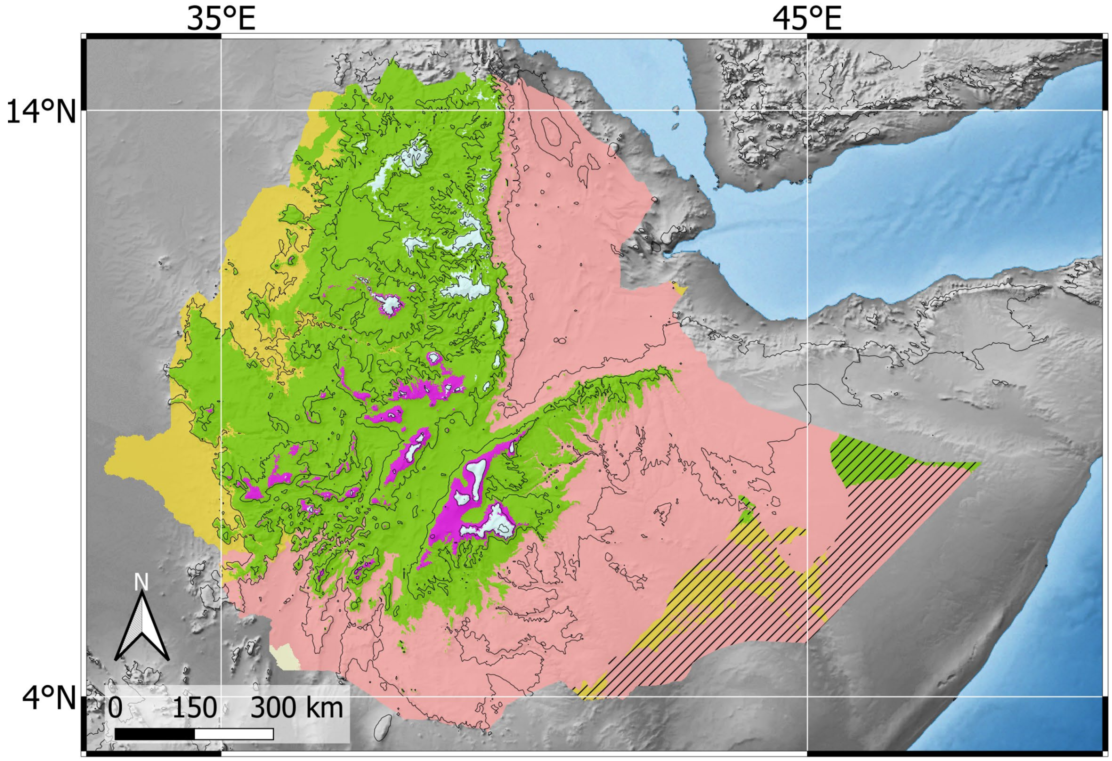

Figure S2.12b. Distribution of vegetation units in Ethiopia at 11 ka with no CO_2 correction.

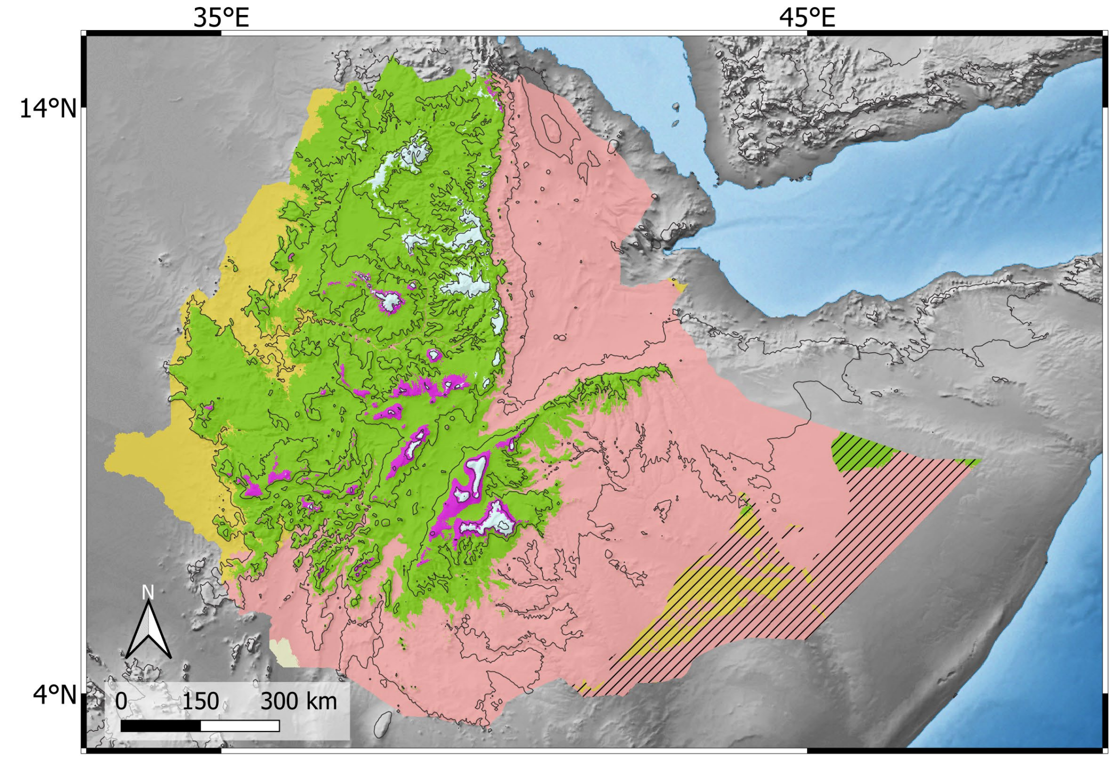

Figure S2.13a. Distribution of vegetation units in Ethiopia at 10 ka with CO_2 correction.

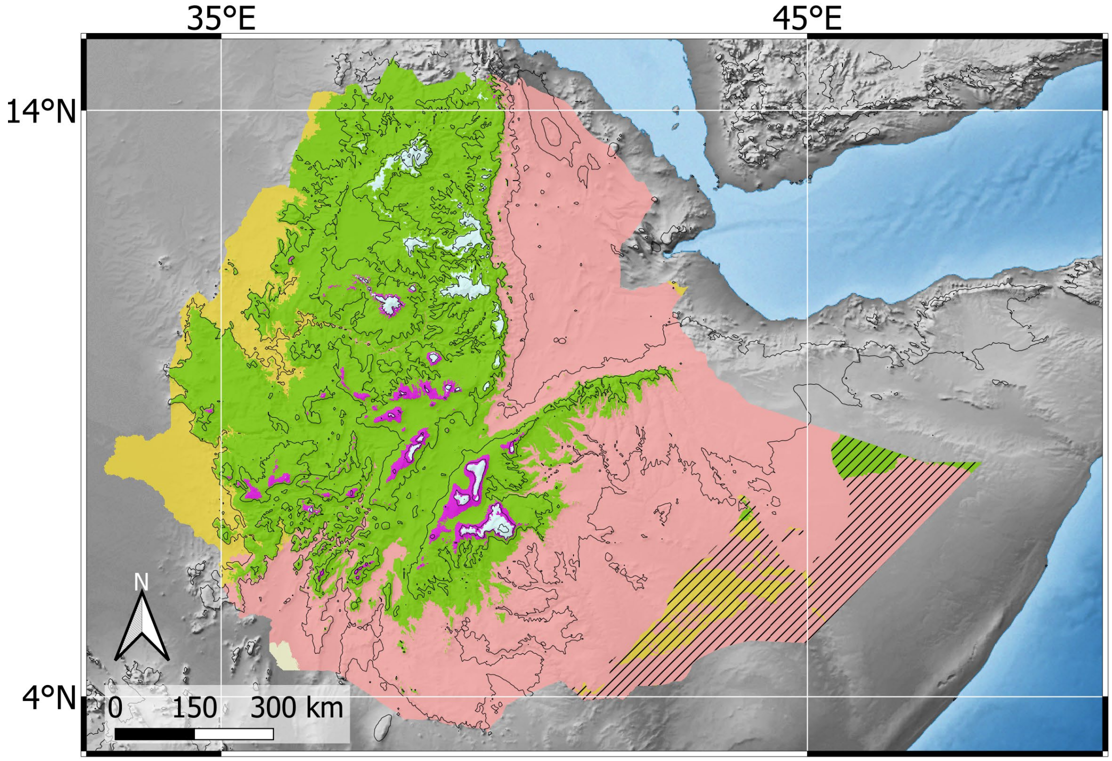

Figure S2.13b. Distribution of vegetation units in Ethiopia at 10 ka with no CO_2 correction.

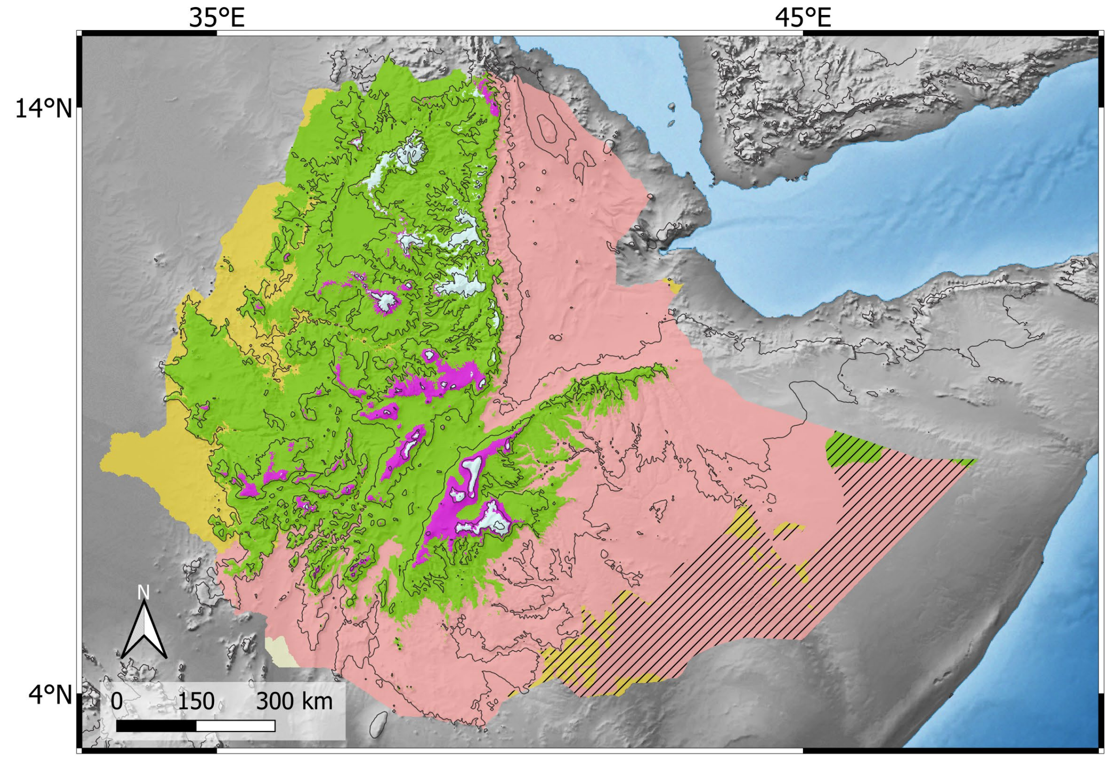

Figure S2.14a. Distribution of vegetation units in Ethiopia at 9 ka with CO_2 correction.

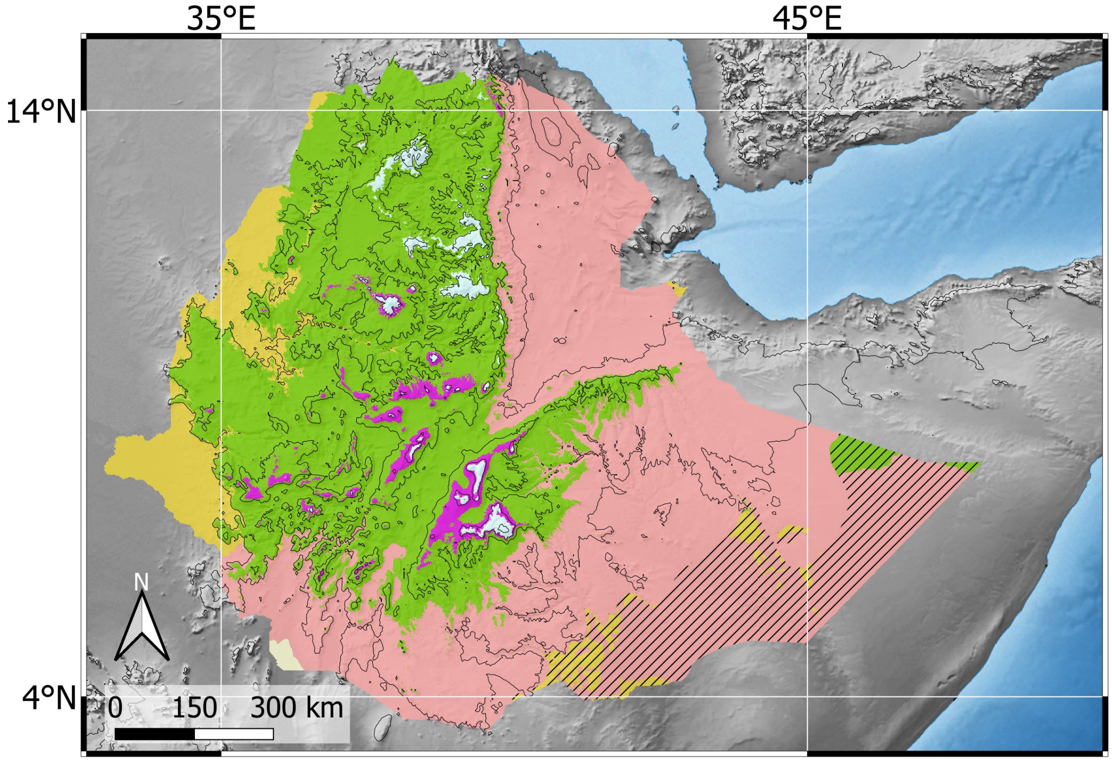

Figure S2.14b. Distribution of vegetation units in Ethiopia at 9 ka with no CO_2 correction.

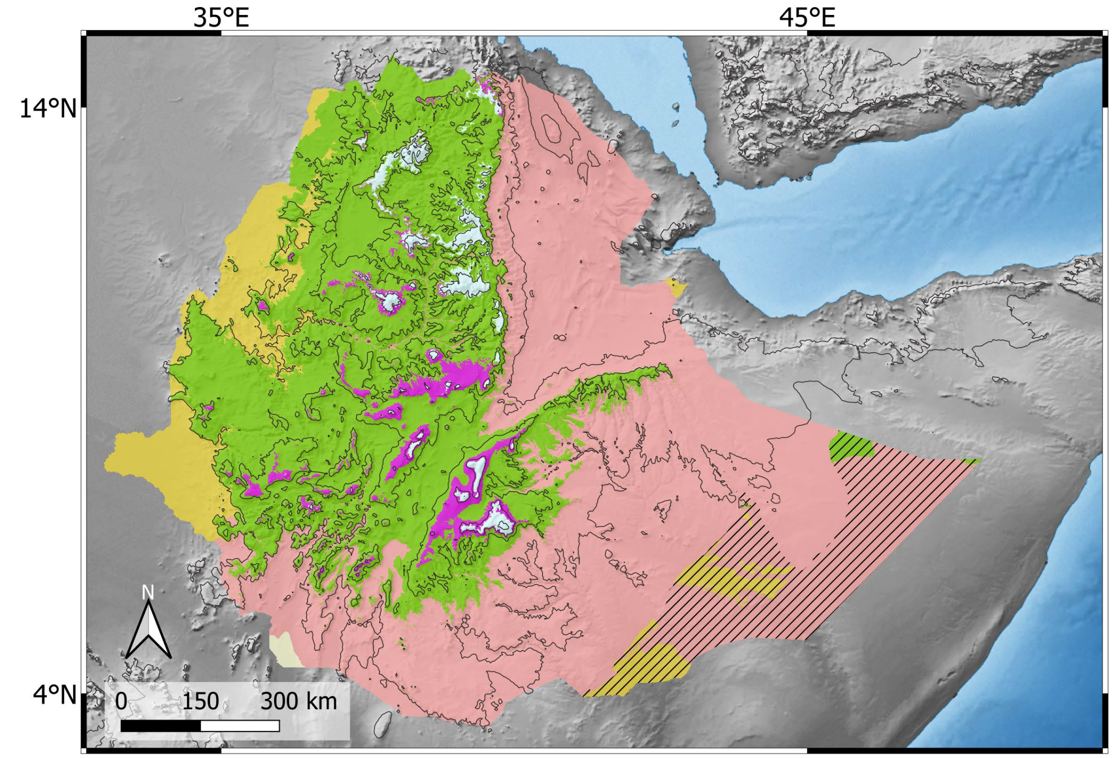

Figure S2.15a. Distribution of vegetation units in Ethiopia at 8 ka with CO_2 correction.

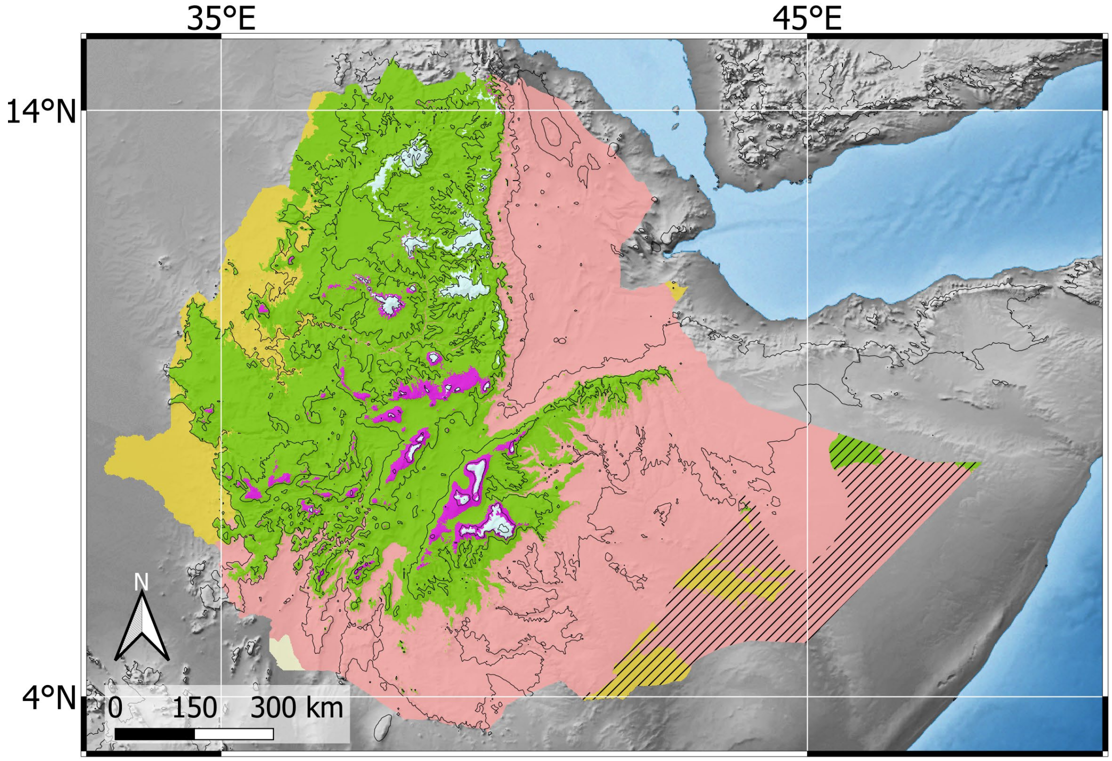

Figure S2.15b. Distribution of vegetation units in Ethiopia at 8 ka with no CO_2 correction.

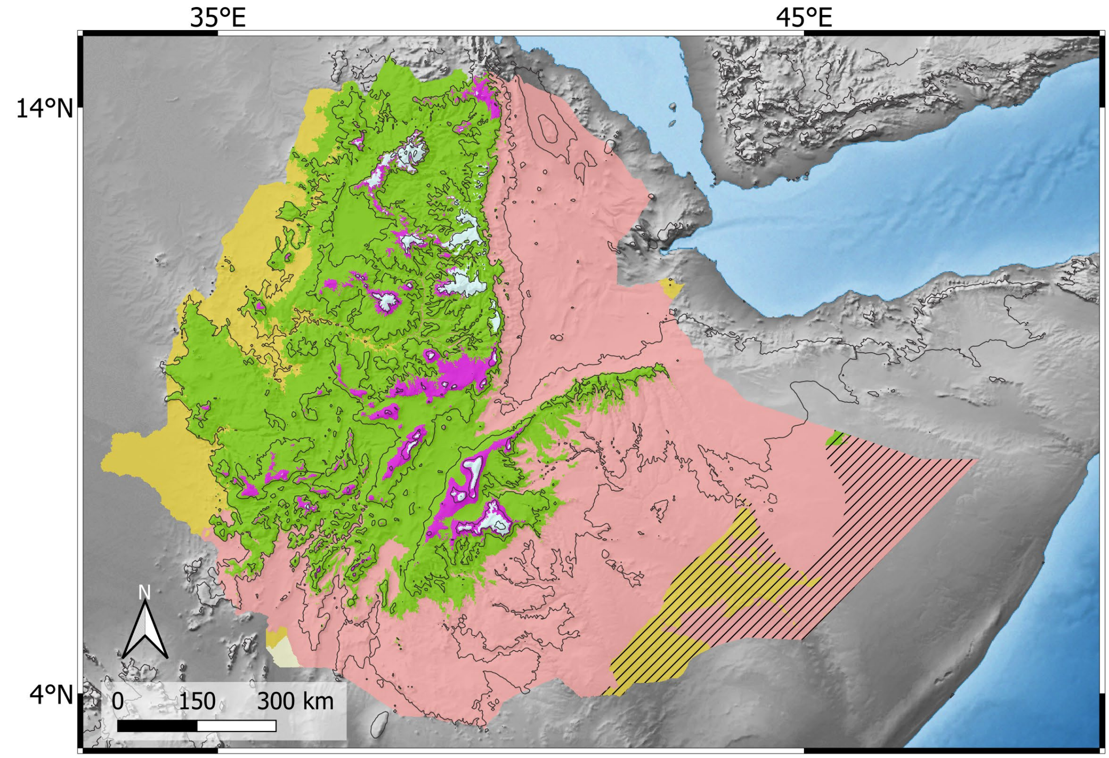

Figure S2.16a. Distribution of vegetation units in Ethiopia at 7 ka ka with CO_2 correction.

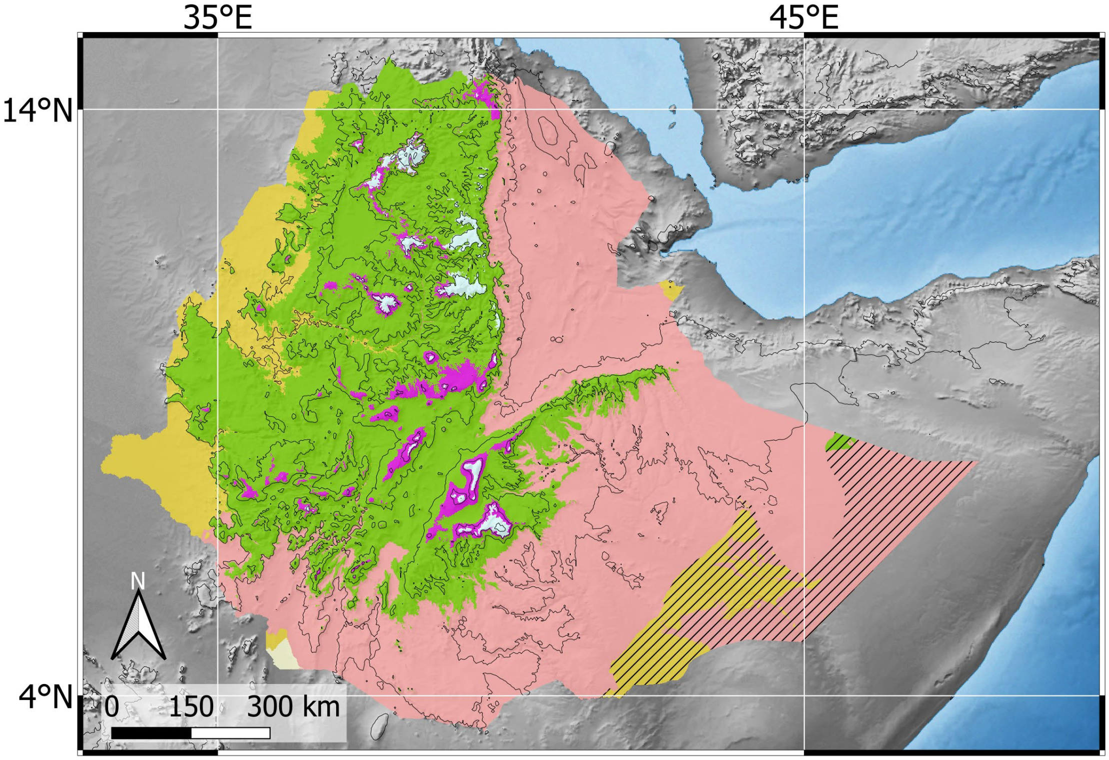

Figure S2.16b. Distribution of vegetation units in Ethiopia at 7 ka ka with no CO_2 correction.

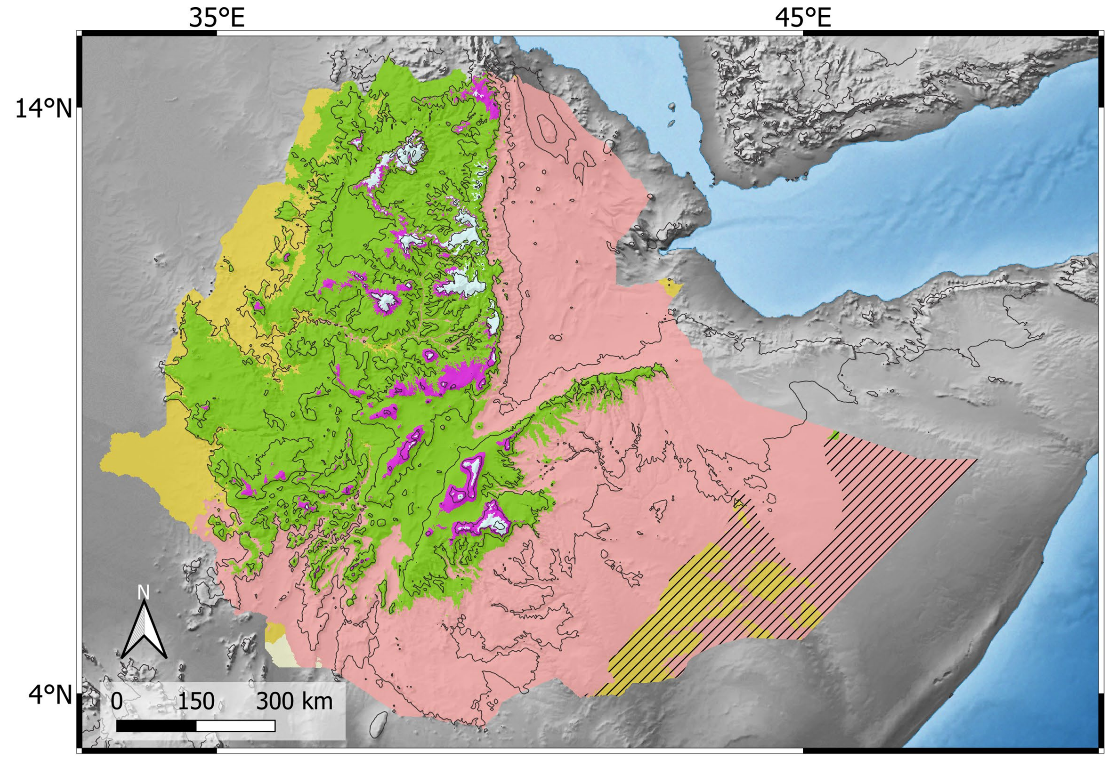

Figure S2.17a. Distribution of vegetation units in Ethiopia at 6 ka with CO_2 correction.

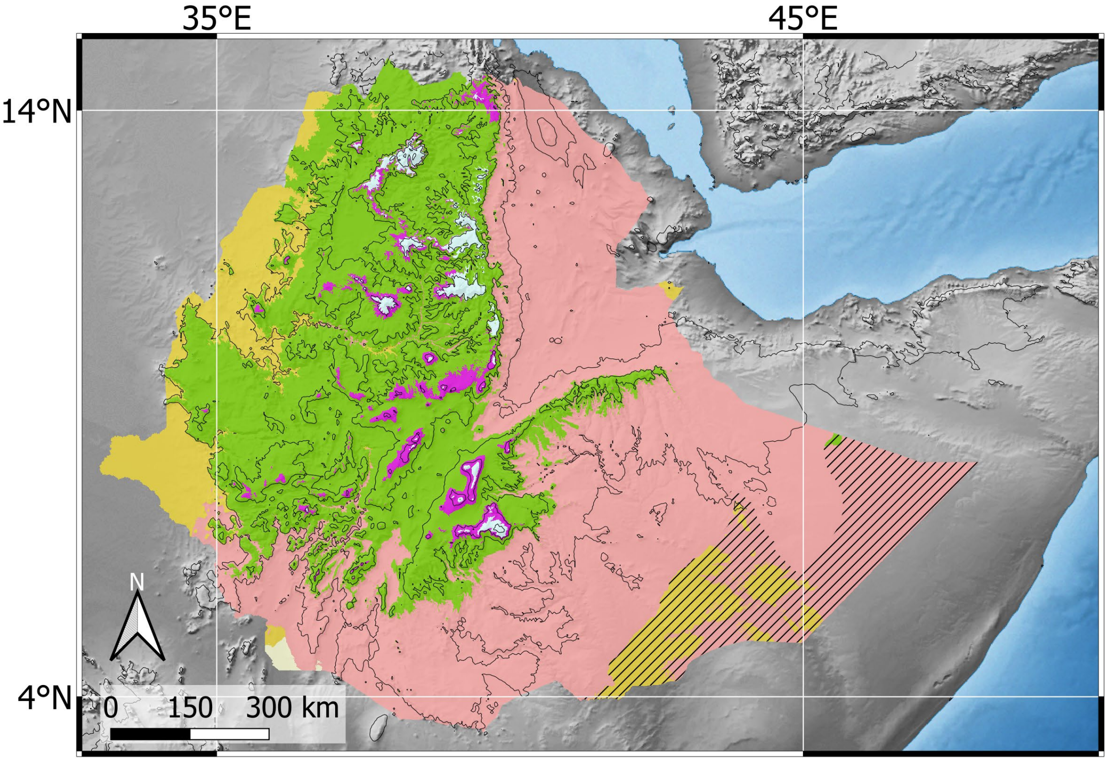

Figure S2.17b. Distribution of vegetation units in Ethiopia at 6 ka with no CO_2 correction.

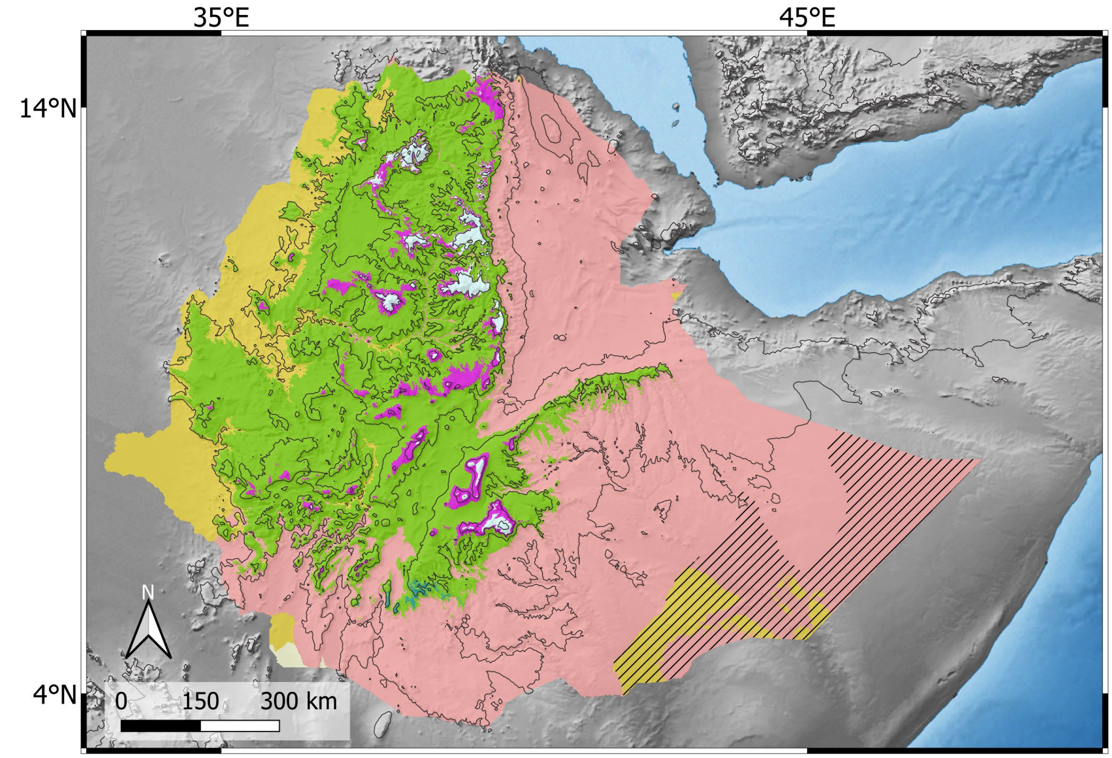

Figure S2.18a. Distribution of vegetation units in Ethiopia at 5 ka with CO_2 correction.

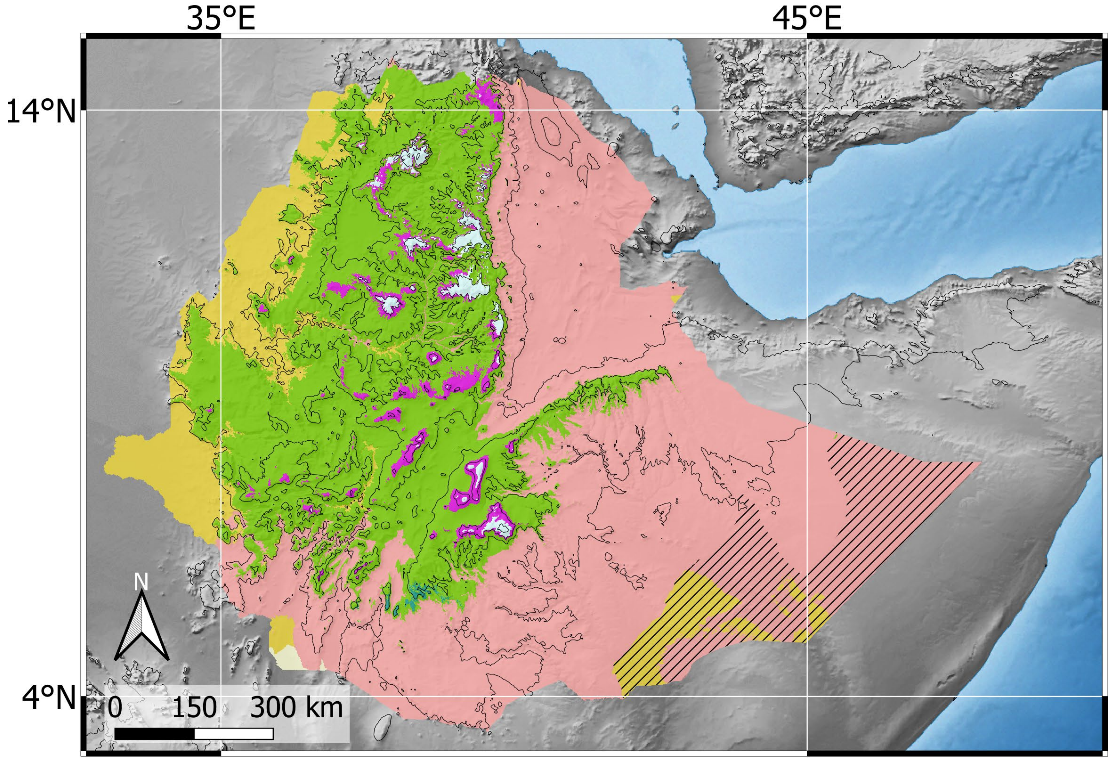

Figure S2.18b. Distribution of vegetation units in Ethiopia at 5 ka with no CO_2 correction.

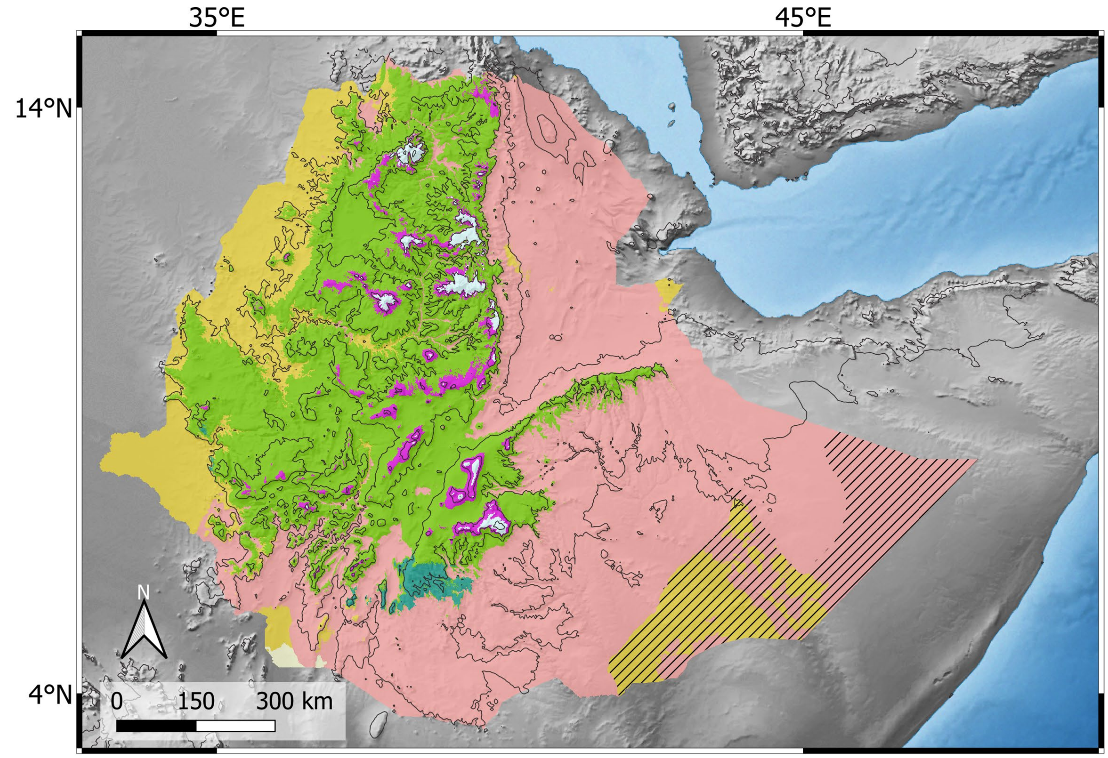

Figure S2.19a. Distribution of vegetation units in Ethiopia at 4 ka with CO_2 correction.

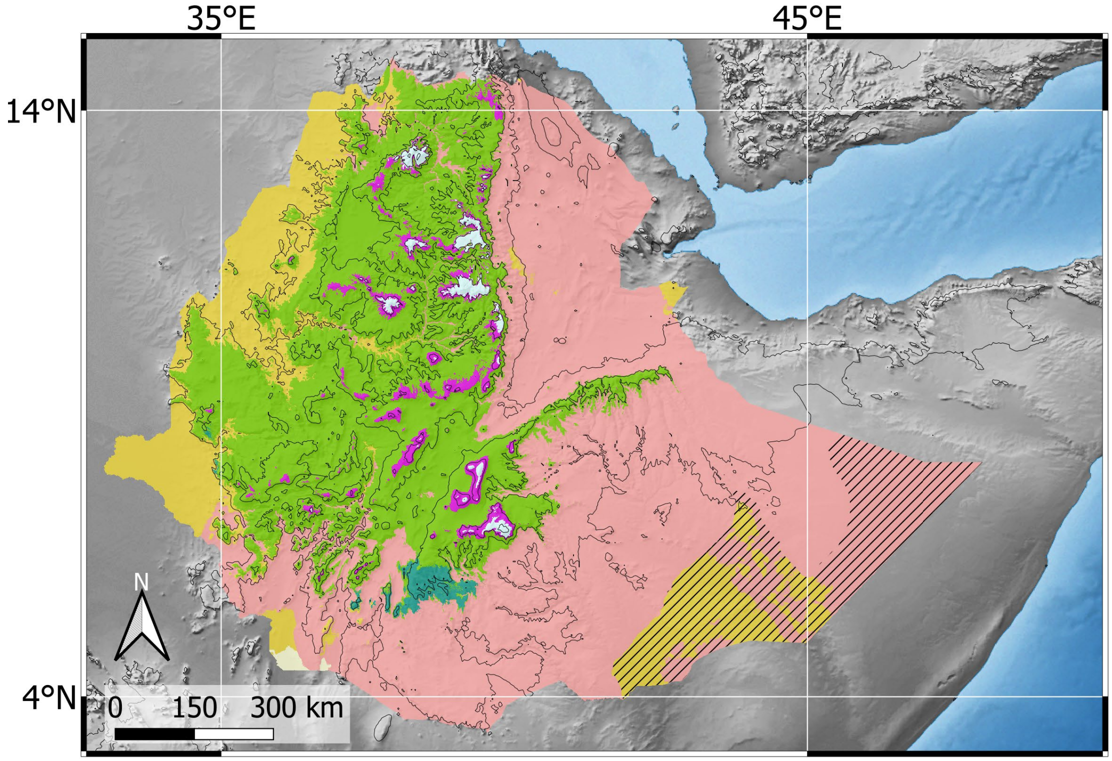

Figure S2.19b. Distribution of vegetation units in Ethiopia at 4 ka with no CO_2 correction.

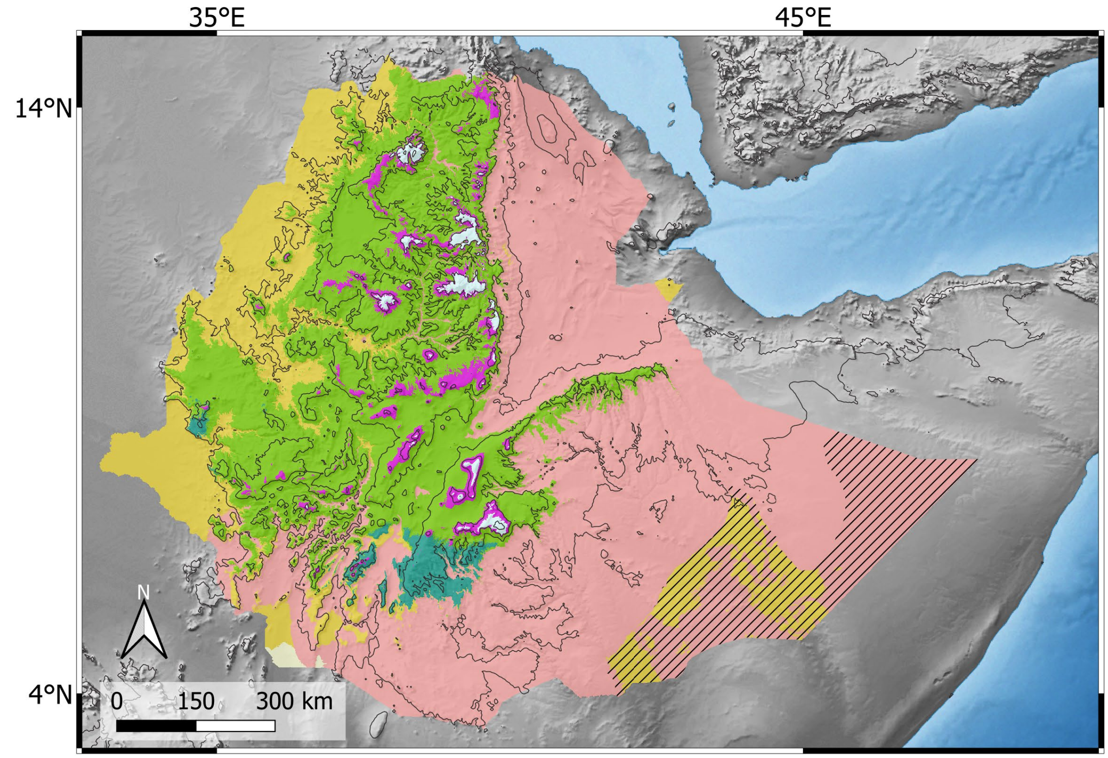

Figure S2.20a. Distribution of vegetation units in Ethiopia at 3 ka with CO_2 correction.

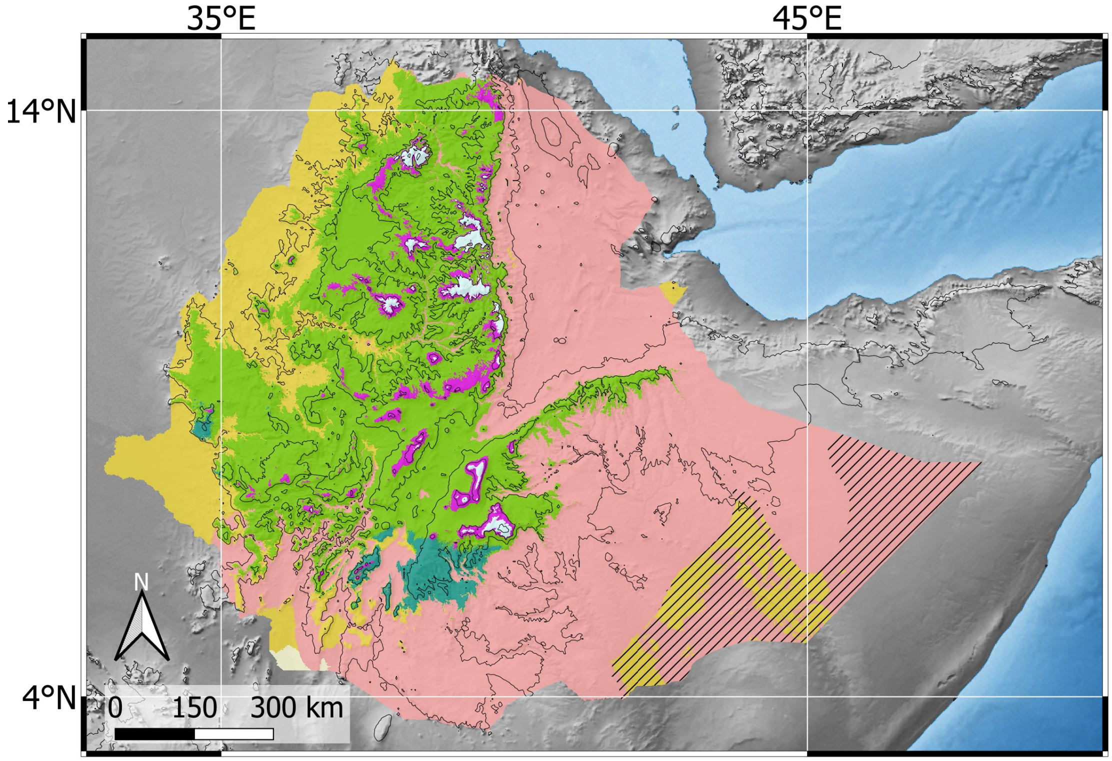

Figure S2.20b. Distribution of vegetation units in Ethiopia at 3 ka with no CO_2 correction.

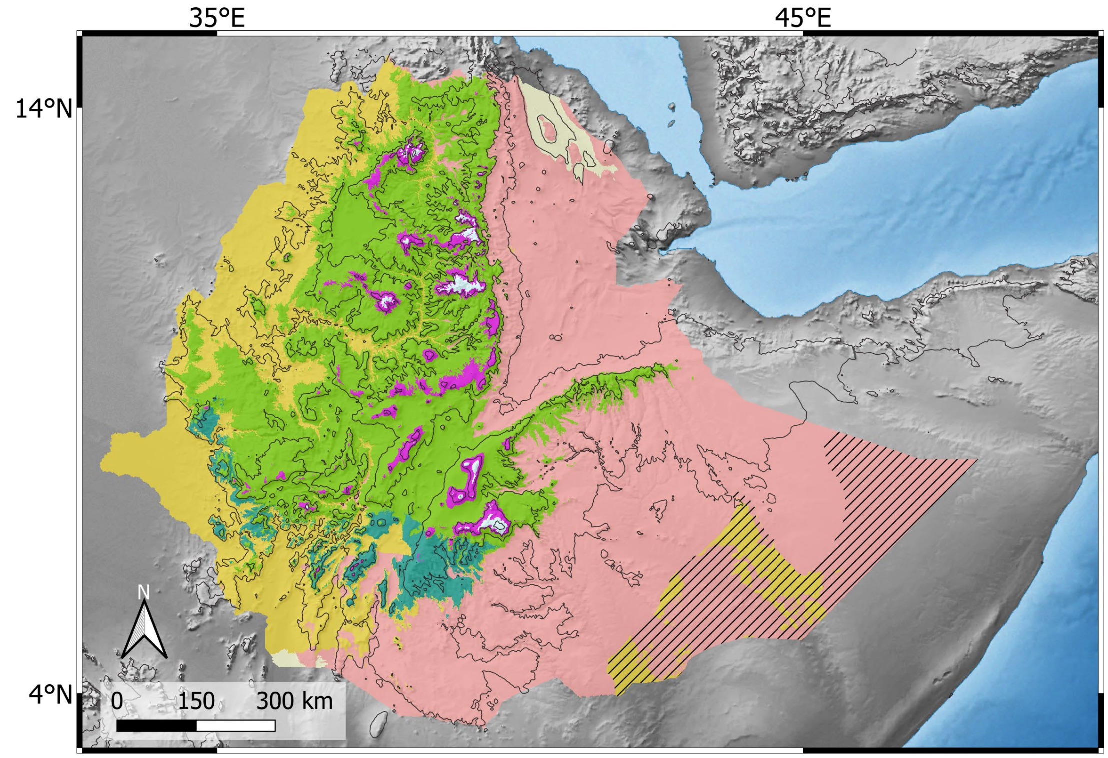

Figure S2.21a. Distribution of vegetation units in Ethiopia at 2 ka with CO_2 correction.

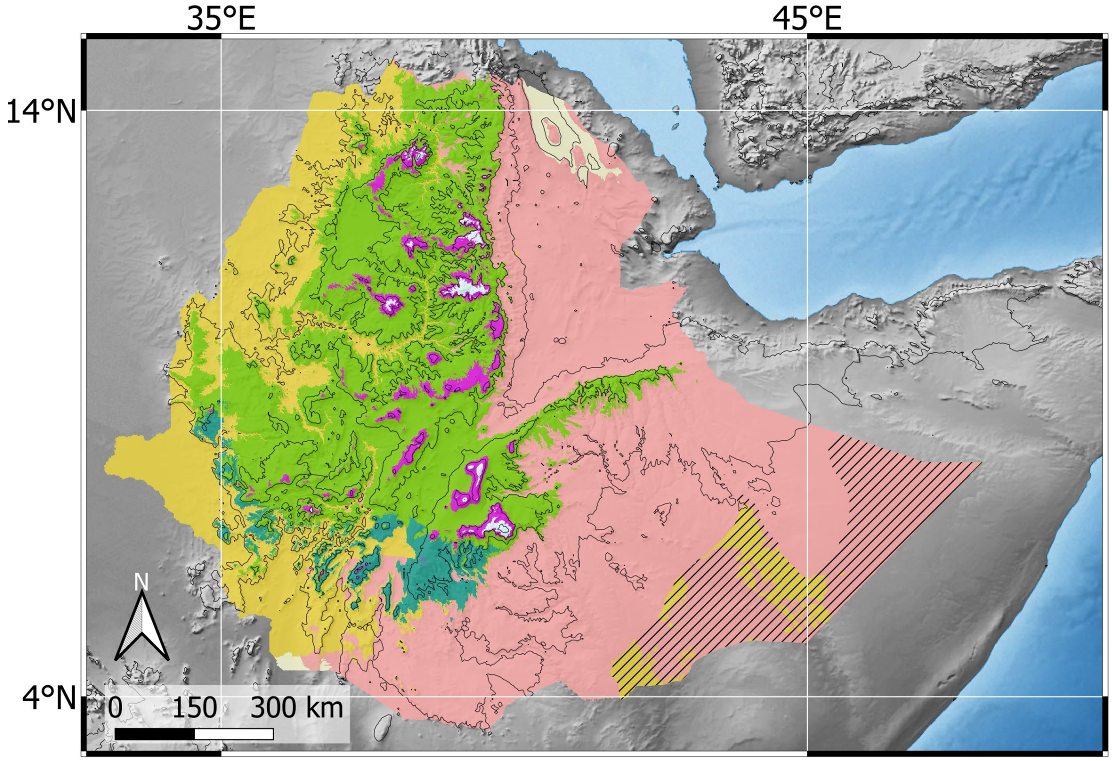

Figure S2.21b. Distribution of vegetation units in Ethiopia at 2 ka with no CO_2 correction.

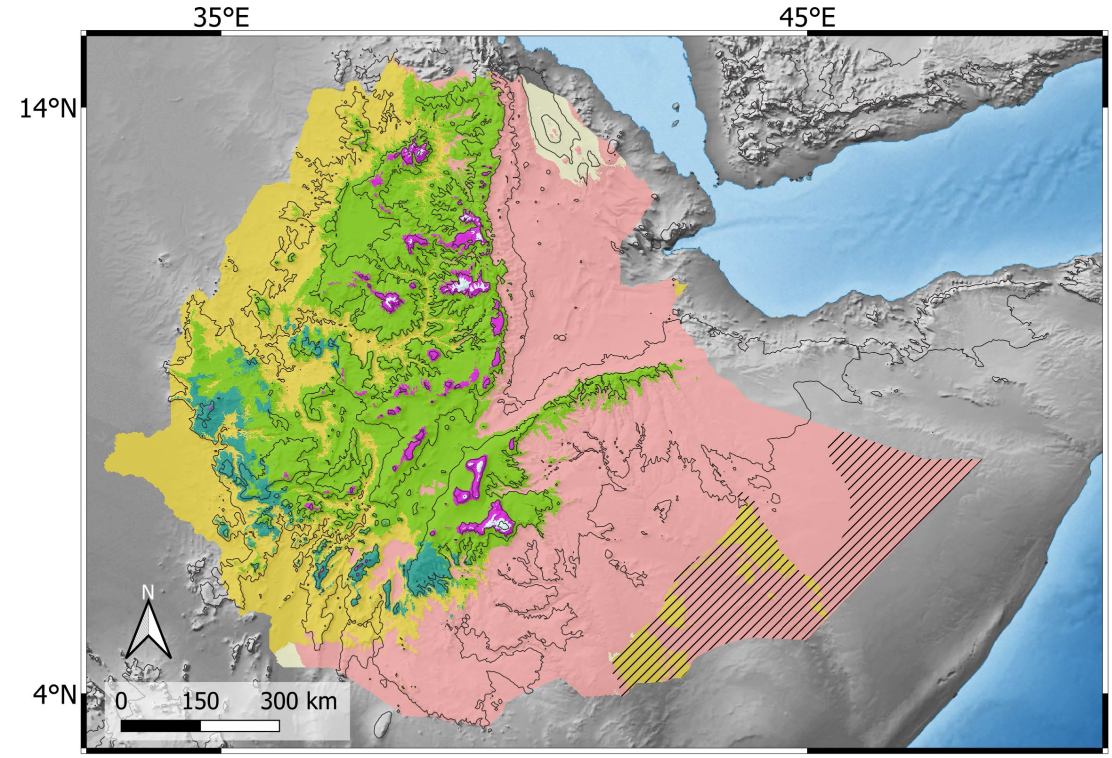

Figure S2.22a. Distribution of vegetation units in Ethiopia at 1 ka with CO_2 correction.

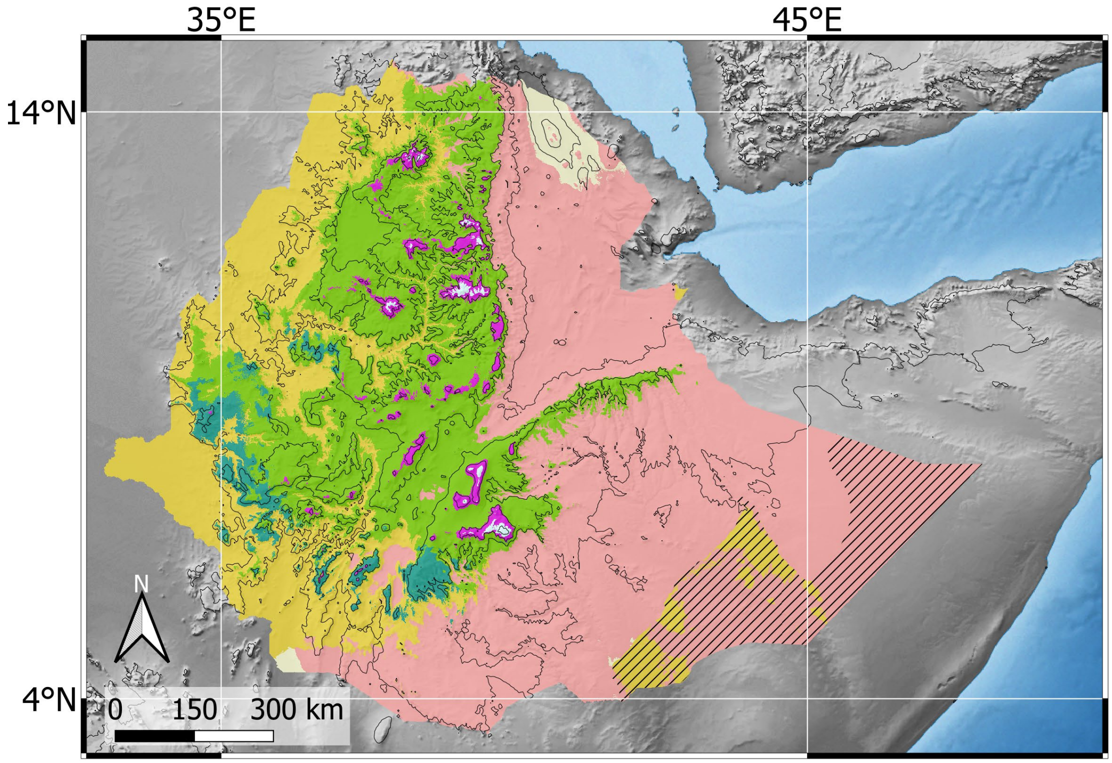

Figure S2.22b. Distribution of vegetation units in Ethiopia at 1 ka with no CO_2 correction.

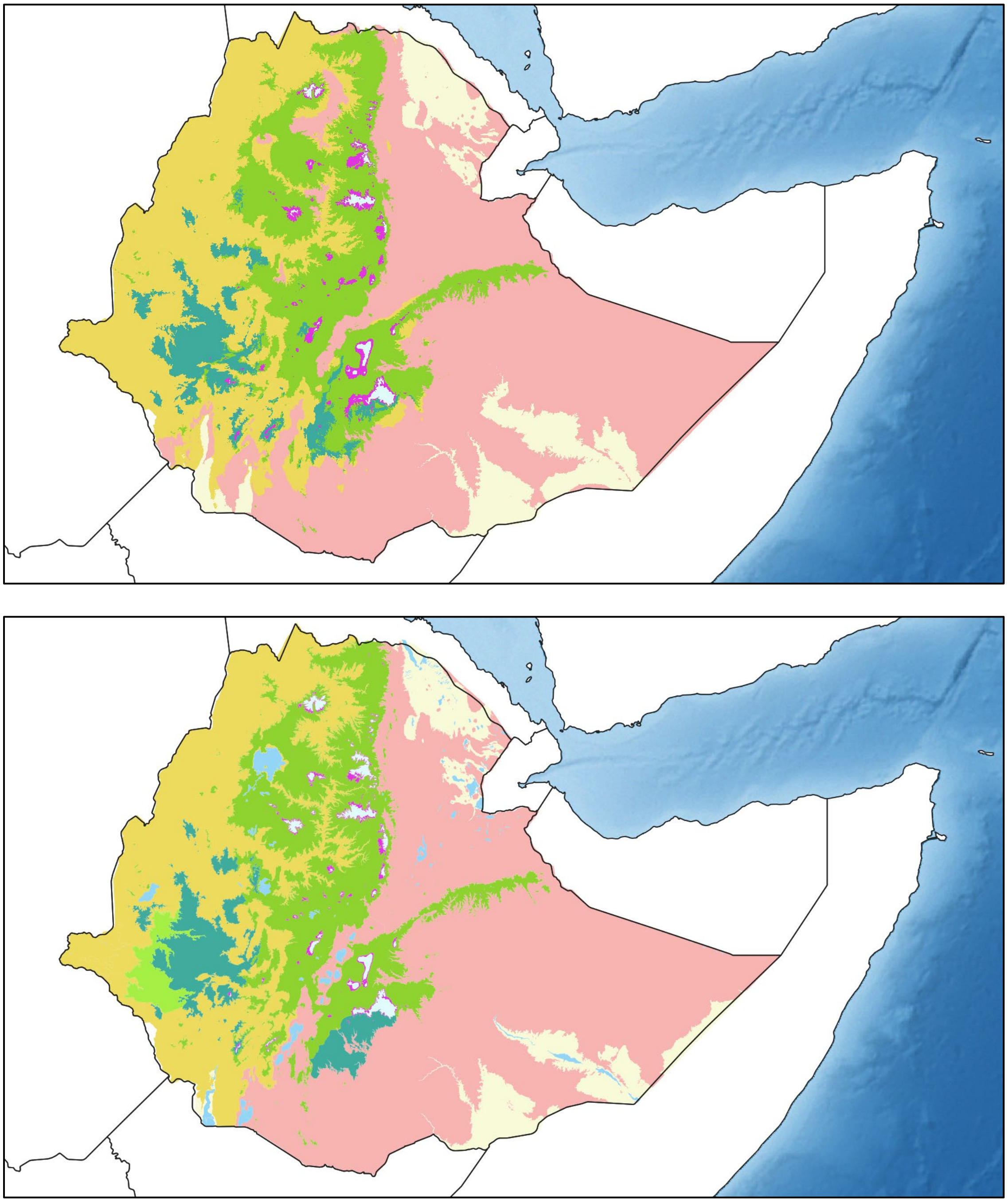

Figure S3. Map produced using the projection of the vegetation unit's models into a present-day (pre-industrial) climatic scenario and selecting the cell grids with higher climatic suitability values of each vegetation unit (*Above*). Map of current potential vegetation distribution in Ethiopia (adapted from Friis *et al*., 2010; *Below*). Vegetation units are color-coded as in figures 2 and 3 of the main text. Maps created by the authors using QGIS v3.16.16 (URL: http://qgis.org).

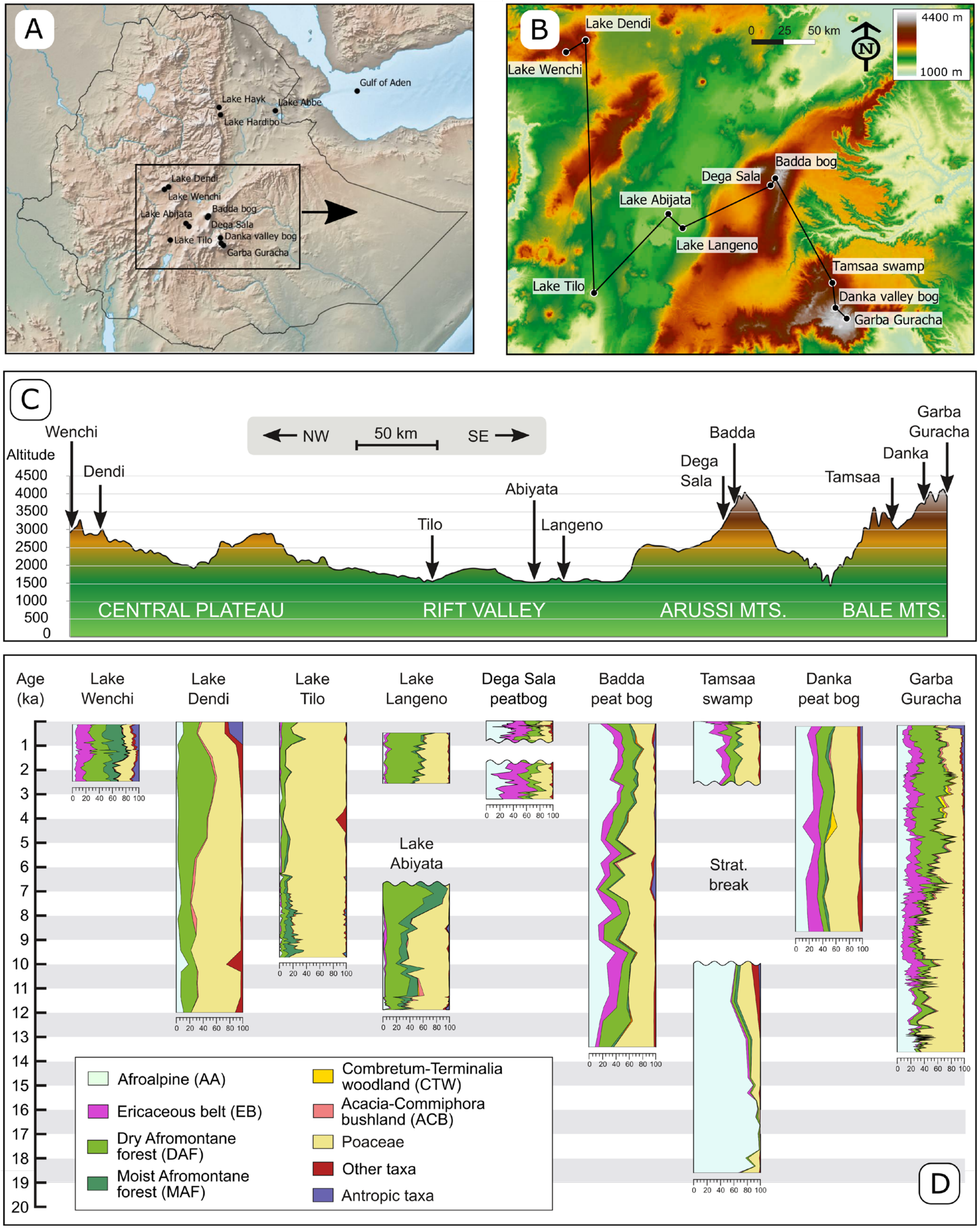

Figure S4. Location of the main Ethiopian fossil pollen records from natural deposits compiled for this study (A). NW to SE transect connecting the major pollen records in Ethiopia and their altitudes (B, C). Compared temporal variations in the abundance of pollen taxa in the main Ethiopian pollen records obtained after assigning the 361 fossil pollen taxa identified in Ethiopia to specific vegetation units (D) (see also Table S5). Maps created using QGIS v3.16.16 (URL: http://qgis.org).

Tables

Table S1. Area under the receiver operating characteristic curve (AUC) values of the models of the final trained models.

**Vegetation unit (VU)**	Model AUC value (training data)
Afroalpine (AA)	0.962
Ericaceous belt (EB)	0.958
Acacia-Commiphora bushland (ACB)	0.711
Combretum-Terminalia woodland (CTW)	0.737
Dry Afromontane forest (DAF)	0.731
Moist Afromontane forest (MAF)	0.934
Desert-Semidesert (DSS)	0.911

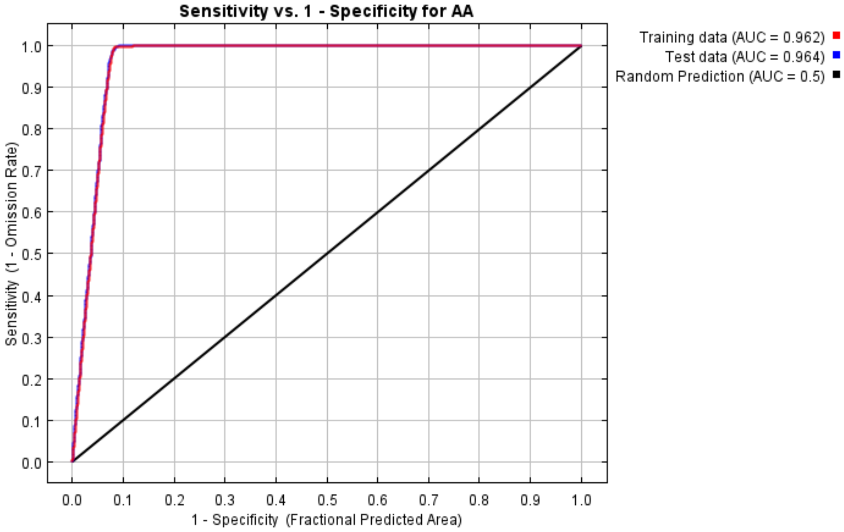

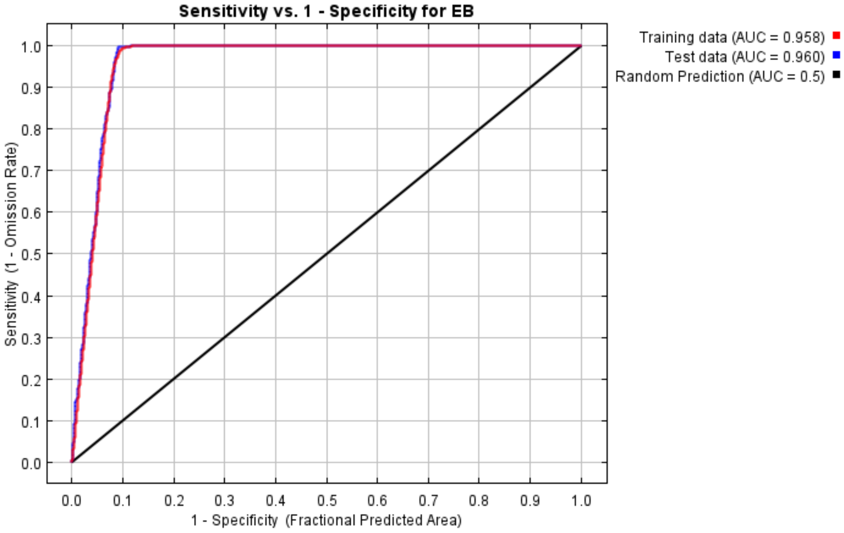

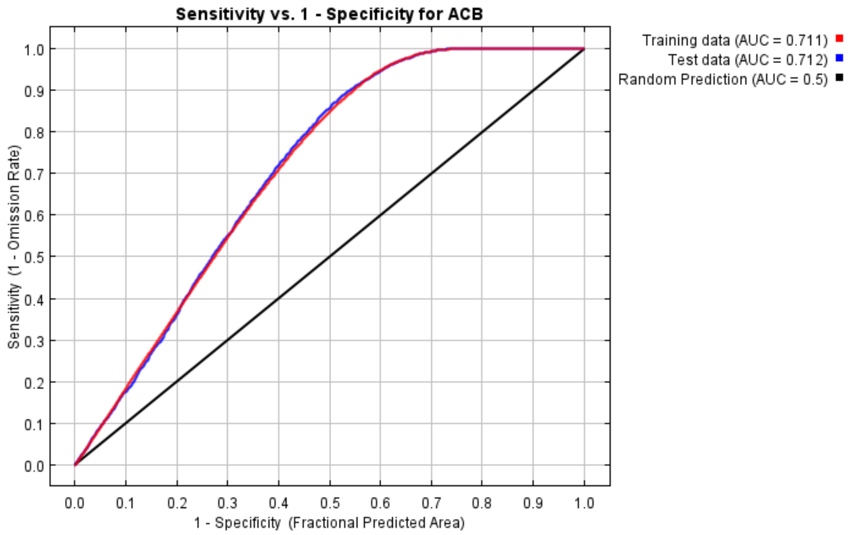
Sensitivity vs. 1 - Specificity for ACB
Training data (AUC = 0.711)
Test data (AUC = 0.712)
Random Prediction (AUC = 0.5)
Sensitivity (1 - Omission Rate)
1 - Specificity (Fractional Predicted Area)
0.0
0.1
0.2
0.3
0.4
0.5
0.6
0.7
0.8
0.9
1.0

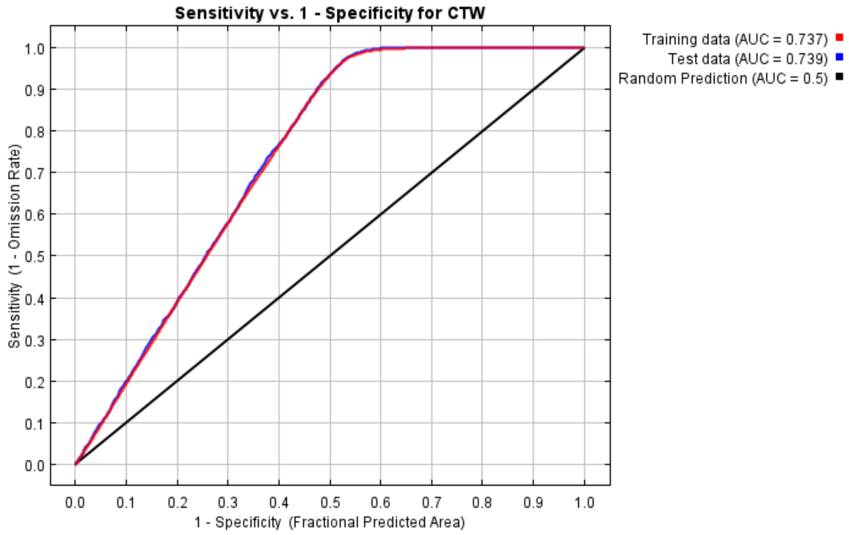
Sensitivity vs. 1 - Specificity for CTW
Training data (AUC = 0.737)
Test data (AUC = 0.739)
Random Prediction (AUC = 0.5)
Sensitivity (1 - Omission Rate)
1 - Specificity (Fractional Predicted Area)
0.0
0.1
0.2
0.3
0.4
0.5
0.6
0.7
0.8
0.9
1.0

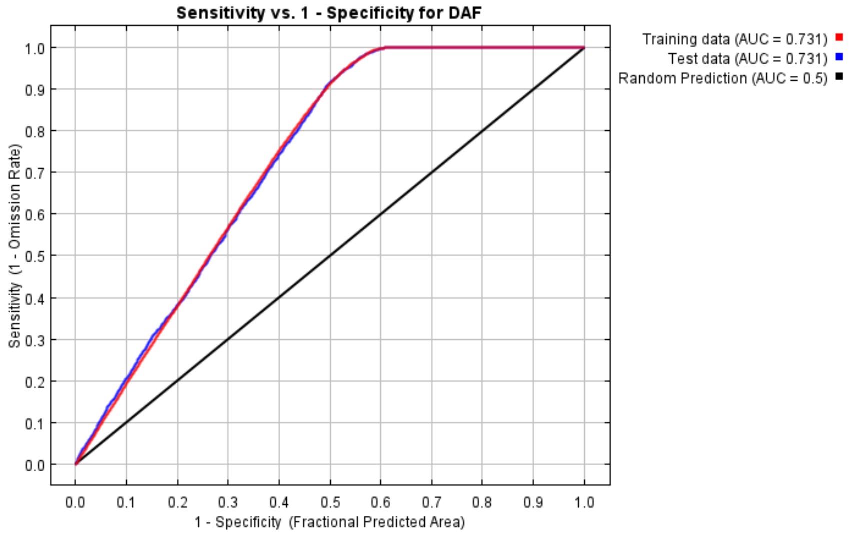
Sensitivity vs. 1 - Specificity for DAF
Training data (AUC = 0.731)
Test data (AUC = 0.731)
Random Prediction (AUC = 0.5)
Sensitivity (1 - Omission Rate)
1 - Specificity (Fractional Predicted Area)
0.0
0.1
0.2
0.3
0.4
0.5
0.6
0.7
0.8
0.9
1.0

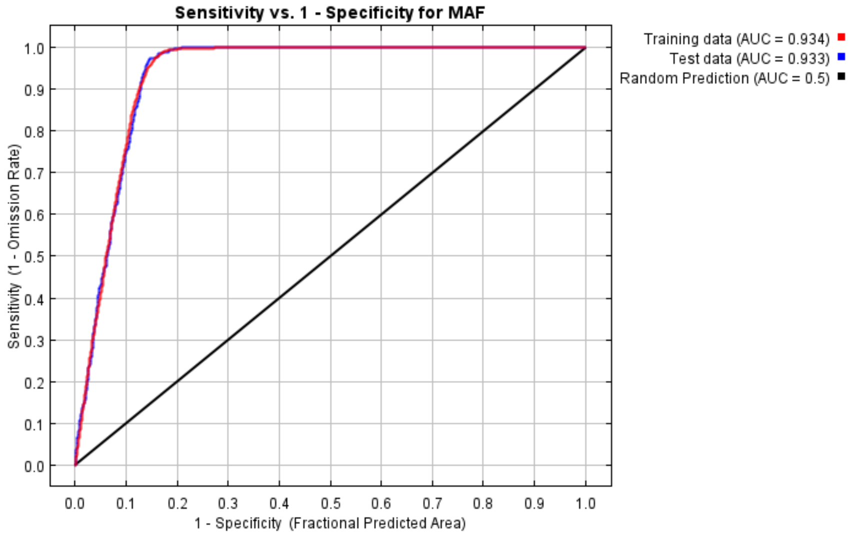
Sensitivity vs. 1 - Specificity for MAF
Training data (AUC = 0.934)
Test data (AUC = 0.933)
Random Prediction (AUC = 0.5)
Sensitivity (1 - Omission Rate)
1 - Specificity (Fractional Predicted Area)
0.0
0.1
0.2
0.3
0.4
0.5
0.6
0.7
0.8
0.9
1.0

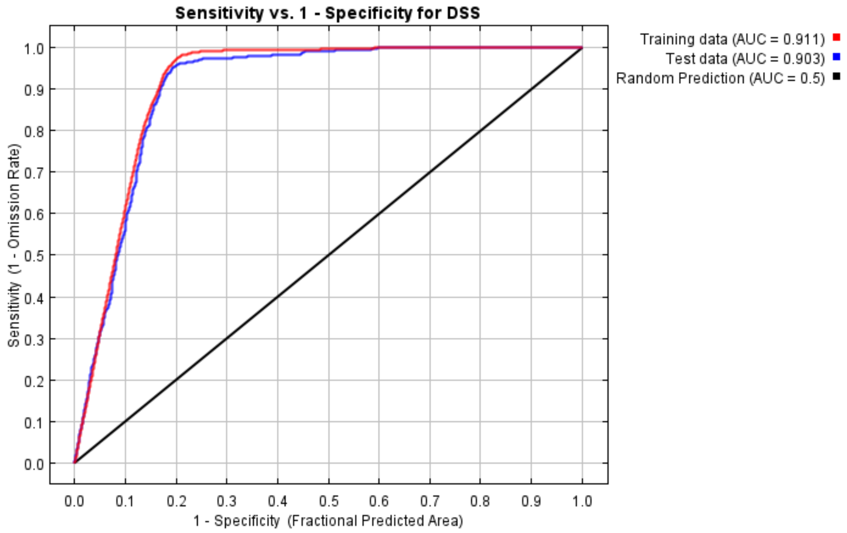
Sensitivity vs. 1 - Specificity for DSS
Training data (AUC = 0.911)
Test data (AUC = 0.903)
Random Prediction (AUC = 0.5)
Sensitivity (1 - Omission Rate)
1 - Specificity (Fractional Predicted Area)
0.0
0.1
0.2
0.3
0.4
0.5
0.6
0.7
0.8
0.9
1.0

Table S2. Relative contribution of the climatic variables to the Maxent models.

Afroalpine (AA) vegetation unit model:

Variable	Percent contribution
Min temperature of coldest month	71.6
Mean temperature of driest quarter	12.3
Precipitation of driest month	8.8
Max temperature of warmest month	4.1
Mean annual temperature	2.5
Mean temperature of wettest quarter	0.5
Precipitation of warmest quarter	0.2
Precipitation of driest month	0.1
Mean annual precipitation	0

Ericaceous belt (EB) vegetation unit model:

Variable	Percent contribution
Min temperature of coldest month	82.5
Mean annual temperature	13.5
Mean temperature of wettest quarter	2
Mean temperature of driest quarter	0.9
Max temperature of warmest month	0.7
Precipitation of driest month	0.2
Precipitation of warmest quarter	0.1
Precipitation of driest month	0.1
Mean annual precipitation	0

Dry Afromontane forest (DAF) vegetation unit model:

Variable	Percent contribution
Min temperature of coldest month	63.7
Mean annual temperature	22.7
Mean temperature of wettest quarter	7.8
Mean temperature of driest quarter	4.5
Precipitation of driest month	0.7
Max temperature of warmest month	0.3
Mean annual precipitation	0.3
Precipitation of driest month	0.1
Precipitation of warmest quarter	0.1

Moist Afromontane forest (MAF) vegetation unit model:

Variable	Percent contribution
Mean annual precipitation	50
Precipitation of driest month	19.7
Min temperature of coldest month	12.7
Mean annual temperature	8.7
Mean temperature of wettest quarter	3.5
Precipitation of warmest quarter	2.8
Mean temperature of driest quarter	1.1
Precipitation of driest month	1.1
Max temperature of warmest month	0.5

Acacia-Commiphora bushland (ACB) vegetation unit model:

Variable	Percent contribution
Min temperature of coldest month	45.1
Mean annual precipitation	32.3
Precipitation of driest month	17.9
Mean temperature of driest quarter	1.5
Mean temperature of wettest quarter	1.3
Precipitation of driest month	0.8
Max temperature of warmest month	0.7
Mean annual temperature	0.4
Precipitation of warmest quarter	0.1

Combretum-Terminalia woodland (CTW) vegetation unit model:

Variable	Percent contribution
Min temperature of coldest month	27.6
Mean annual precipitation	24.3
Precipitation of warmest quarter	11.3
Mean temperature of driest quarter	10.2
Precipitation of driest month	9.1
Max temperature of warmest month	8.6
Mean temperature of wettest quarter	4.4
Precipitation of driest month	3.9
Mean annual temperature	0.5

Desert-semidesert (DSS) vegetation unit model:

Variable	Percent contribution
Mean annual precipitation	35.7
Max temperature of warmest month	22.4
Min temperature of coldest month	10.2
Precipitation of driest month	6.9
Mean annual temperature	6.5
Mean temperature of wettest quarter	6.5
Precipitation of warmest quarter	5.9
Precipitation of driest month	5.3
Mean temperature of driest quarter	0.7

Table S3. Calculation of the correction factors introduced in order to consider the impact of CO_2 on the Ethiopian vegetation units analyzed. The correction factors were calculated by dividing the fraction covered by each biome in Woillez et al (2012) modeled using LGM CO_2 levels, by the fraction cover modeled using present-day CO_2 levels. The biomes considered to be equivalent to the Ethiopian vegetation units analyzed in this study are indicated below. DAF= Dry Afromontane Forest; MAF= Moist Afromontane Forest; CTW= *Combretum-Terminalia* Woodland; ACB= *Acacia-Commiphora* Bushland; DSS= Desert-Semidesert; EB= Ericaceous Belt; AA= Afroalpine.

Ethiopian vegetation unit	Biome in Woillez et al. (2012)	Correction factor for the LGM (22 ka time slice)	
DAF	TempBE (50%) + TRBE (50%)	55/69=	**0.8**
MAF	TRBE	40/58=	**0.69**
CTW, ACB & DSS	C4 (50%) + TRBR (50%)	34/22=	**1.54**
EB	TempBE (75%) + C3 grass (25%)	18.75/13.5=	**1.39**
AA	C3 grass	30/18=	**1.67**

Cover fraction of plant functional types during the LGM with present (pre-industrial) level and with LGM level, according to Woillez et al. (2011).

	Pre-industrial CO_2 level (280-310 ppm)			LGM CO_2 level (180-185 ppm)		
PFT	Tropical (%)	Temperate (%)	Boreal (%)	Tropical (%)	Temperate (%)	Boreal (%)
Tropical broadleaf evergreen (TRBE)	57	<1		39	<1	
Tropical broadleaf raingreen (TRBR)	18			11		
Temperate needleleaf evergreen (TempNE)	6	11	2		3	<1
Temperate broadleaf evergreen (TempBE)		12		6	9	
Temperate broadleaf summergreen (TempBS)	2	19	2	1	17	2
Boreal needleleaf evergreen (BoNE)		12	10		5	2
Boreal broadleaf summergreen (BoBS)		18	21		28	18
Boreal needleleaf summergreen (BoNS)			3			<1
C_3 grass	1	2	15	4	3	23
C_4 grass	4			22	<1	
Bare soil	12	10	18	17	19	25
Ice		15	29		15	29

Linear relation between atmospheric CO_2 concentration and intensity of the physiological effect on vegetation growth (correction factor) for each Ethiopian vegetation unit.

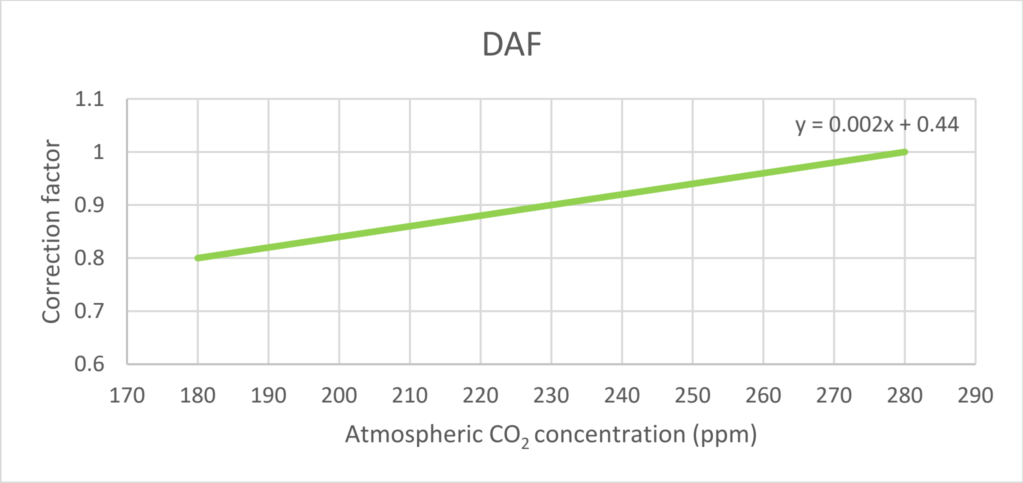

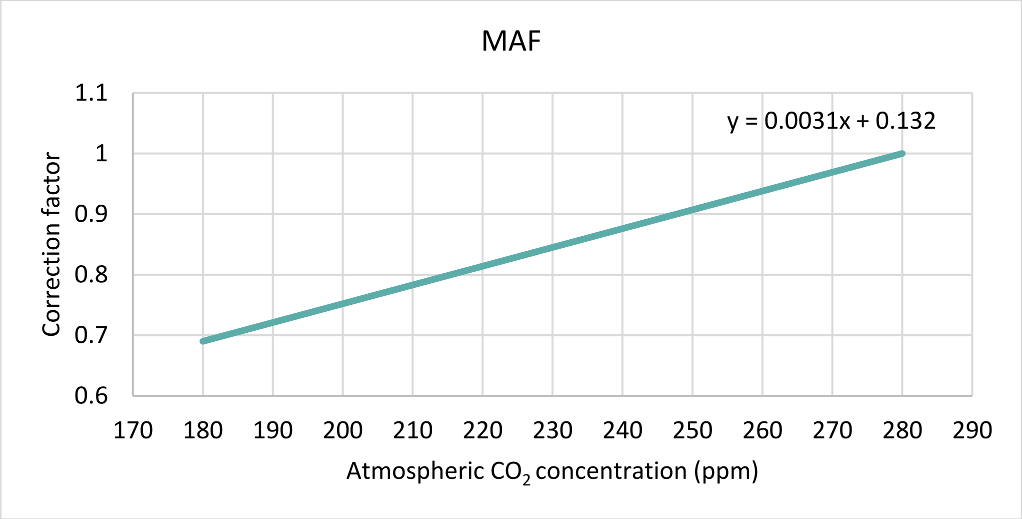

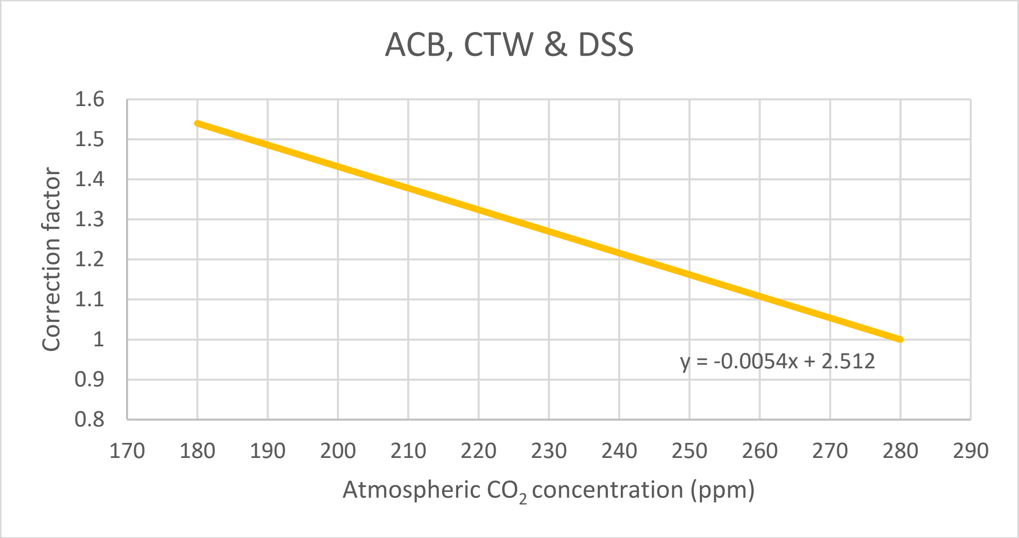

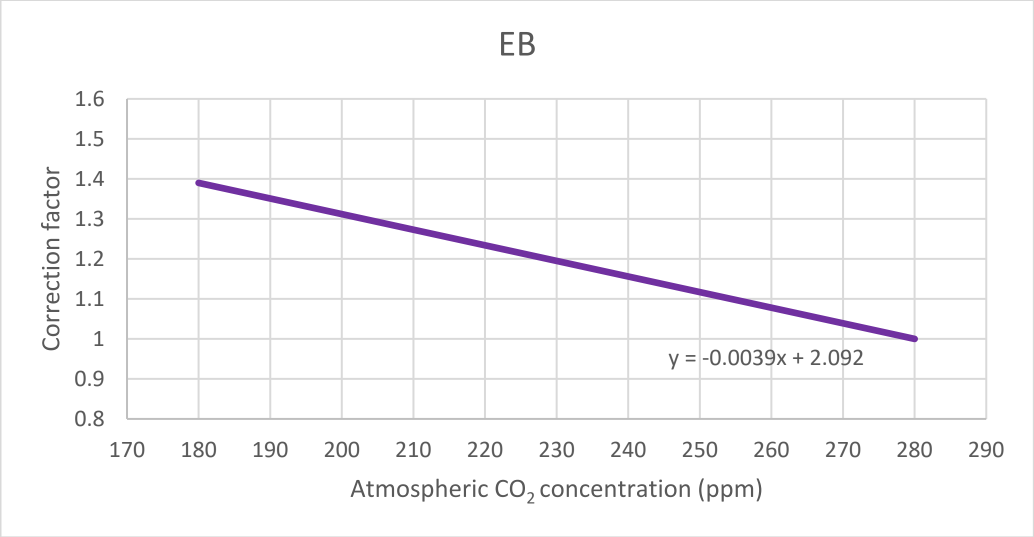

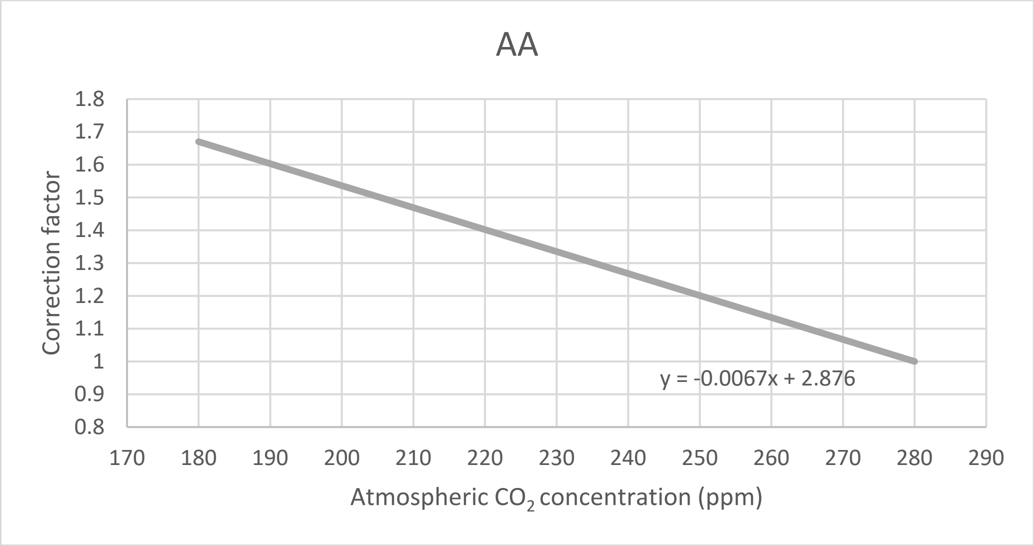

Table: Correction factors calculated for each time slice assuming a linear relation between CO_2 concentration and intensity of the physiological effect on vegetation growth. Atmospheric CO_2 concentration levels estimated are based on Yu et al. (2020).

**Time slice (ka)**	**CO_2 concentration (ppm)**	**DAF**	**MAF**	**ACB, CTW & DSS**	**EB**	**AA**
22	180	0.8	0.69	1.54	1.39	1.67
21	189	0.818	0.7179	1.4914	1.3549	1.6097
20	193	0.826	0.7303	1.4698	1.3393	1.5829
19	191	0.822	0.7241	1.4806	1.3471	1.5963
18	187	0.814	0.7117	1.5022	1.3627	1.6231
17	204	0.848	0.7644	1.4104	1.2964	1.5092
16	223	0.886	0.8233	1.3078	1.2223	1.3819
15	228	0.896	0.8388	1.2808	1.2028	1.3484

14	238	0.916	0.8698	1.2268	1.1638	1.2814
13	239	0.918	0.8729	1.2214	1.1599	1.2747
12	250	0.94	0.907	1.162	1.117	1.201
11	267	0.974	0.9597	1.0702	1.0507	1.0871
10	264	0.968	0.9504	1.0864	1.0624	1.1072
9	261	0.962	0.9411	1.1026	1.0741	1.1273
8	260	0.96	0.938	1.108	1.078	1.134
7	260	0.96	0.938	1.108	1.078	1.134
6	266	0.972	0.9566	1.0756	1.0546	1.0938
5	270	0.98	0.969	1.054	1.039	1.067
4	272	0.984	0.9752	1.0432	1.0312	1.0536
3	276	0.992	0.9876	1.0216	1.0156	1.0268
2	278	0.996	0.9938	1.0108	1.0078	1.0134
1	279	0.998	0.9969	1.0054	1.0039	1.0067
Present (preindustrial) 0	280	1	1	1	1	1

Table S4. Details of the compiled fossil pollen records in Ethiopia. The raw palynological data (original absolute counts of pollen grains) are available to download at the African Pollen Database (https://africanpollendatabase.ipsl.fr/#/pollen-fossile).

Pollen record	Region	Altitude (m)	Geographical coordinates		Depositional setting	no. samples
Badda bog	Arussi Mountains	4040	39.366667	7.866667	Peat bog	30
Garba Guracha	Bale Mountains	3950	39.866667	6.883333	Lake	259
Danka valley bog	Bale Mountains	3830	39.788000	6.958000	Peat bog	13
Dega Sala	Arussi Mountains	3600	39.333300	7.816600	Swamp	54
Tamsaa swamp	Bale Mountains	3000	39.766667	7.133333	Swamp	36
Lake Wenchi	Central Ethiopian Plateau	2900	37.900000	8.750000	Lake	38
Lake Dendi	Central Ethiopian Plateau	2836	38.037206	8.833330	Lake	19
Lake Hardibo	Northern Ethiopia	2150	39.766600	11.233300	Lake	15
Lake Hayk	Northern Ethiopia	1920	39.720000	11.480000	Lake	30
Lake Langeno	Rift Valley	1583	38.716660	7.516666	Lake	25
Lake Abijata	Rift Valley	1578	38.616660	7.616660	Lake	186
Lake Tilo	Rift Valley	1545	38.095833	7.062500	Lake	70
Lake Abbe	Djibouti	240	41.593938	11.370065	Lake	3
Gulf of Aden	Gulf of Aden	0	44.317200	12.025300	Marine	95

Table S5. Assignment of the taxa identified in Ethiopian fossil pollen studies to one of the vegetation units modelled in this study. Taxa are grouped according to their ecological preference Out of the 361 taxa identified in Ethiopia, 240 with clear ecological significance were assigned to one of the VUs analyzed. Taxa characteristic of more than one VU or with unclear ecological significance were excluded from the assignment (others).

Fossil pollen as identified originally	Extant taxa	VU assignment
Acacia	Vachellia	ACB
Acalypha	Acalypha	CTW
Acanthaceae	Acanthaceae	Others
Acanthus	Acanthus	DAF
Achyranthes-type aspera (Amaranthaceae)	Achyranthes	Others
Adenia type (Passifloraceae)	Adenia	ACB
Adenium-type obesum (Apocynaceae)	Adenium	ACB
Aerva-type javanica (Amaranthaceae)	Aerva	Others
Aerva-type lanata (Amaranthaceae)	Aerva	Others
Agavaceae	Agavaceae	Others
Aidia-type micrantha (Rubiaceae)	Aidia	Others
Aizoaceae	Aizoaceae	Others
Alchemilla (Rosaceae)	Alchemilla	AA
Alchornea	Alchornea	DAF
Allophylus	Allophylus	Others
Allophylus abyssinicus (Sapindaceae)	Allophylus abyssinicus	DAF/MAF
Alnus	Alnus	Others
Aloe	Aloe	Others
Ambrosieae	Ambrosieae	Others
Anacardiaceae	Anacardiaceae	Others
Anagallis-type	Anagallis	Others
Aningeria-type	Pouteria	MAF
Anthocerotaceae	Anthocerotaceae	Others
Anthospermum (Rubiaceae)	Anthospermum	EB
Anthyllis (Fabaceae)	Anthyllis	DAF/MAF
Apiaceae	Apiaceae	AA
Apodytes	Apodytes	DAF/MAF
Apodytes dimidiata	Apodytes dimidiata	DAF/MAF
Araliaceae	Araliaceae	Others
Artemisia	Artemisia	AA
Asparagus-type buchananii	Asparagus	Others
Asphodelus	Asphodelus	DAF
Aster	Aster	AA
Asteraceae	Asteraceae	AA
Asteroideae	Asteroideae	AA
Balanites /Zygophyllaceae	Balanites	ACB
Balanites aegyptiaca-type	Balanites aegyptiaca	ACB

Balanites rotundifolia-type	Balanites rotundifolia	ACB
Barbeya oleoides	Barbeya oleoides	ACB
Barbeyaceae	Barbeyaceae	Others
Basilicum-type polystachyon (Lamiaceae)	Basilicum	Others
Begonia	Begonia	MAF
Bersama (Francoaceae)	Bersama	DAF/MAF
Bersama abyssinica-type	Bersama abyssinica	DAF/MAF
Blepharis (Acanthaceae)	Blepharis	ACB
Borassus-type aethiopum	Borassus	CTW
Boscia-type (Capparaceae)	Boscia	ACB
Bosqueia (Moraceae)	Bosqueia (=Trilepisium)	MAF
Botryococcus	Botryococcus	Algae, others
Brassicaceae	Brassicaceae	ACB
Bridelia	Bridelia	CTW
Brucea (Simaroubaceae)	Brucea	DAF/MAF
Brucea antidysenterica-type	Brucea	DAF/MAF
Buddleja	Buddleja	EB
Buxus	Buxus	DAF
Buxus hildebrandtii-type	Buxus	DAF
Caesalpiniaceae	Caesalpiniaceae	Others
Callitriche	Callitriche	AA
Calpurnia-type aurea	Calpurnia	DAF
Campanula	Campanula	Others
Canthium	Canthium	DAF/MAF
Canthium gueinzii-type	Canthium	DAF/MAF
Canthium schimperianum-type	Canthium	DAF/MAF
Capitanya	Capitanya (=Plectranthus)	Others
Capparidaceae	Capparidaceae	ACB
Capparis	Capparis	ACB
Capparis fascicularis-type	Capparis fascicularis	ACB
Capparis tomentosa-type	Capparis	ACB
Carduus	Carduus	AA
Carissa	Carissa	Others
Carissa-type edulis	Carissa	Others
Caryophyllaceae	Caryophyllaceae	AA
Cassia	Cassia	CTW
Cassia-type italica	Cassia	CTW
Casuarina	Casuarina	Others
Caucanthus-type	Caucanthus	ACB
Celastraceae	Celastraceae	DAF
Celosia (Amaranthaceae)	Celosia	Others
Celosia-type argentea	Celosia	Others
Celosia-type trigyna	Celosia	Others

Celtis	Celtis	DAF/MAF
Celtis africana-type	Celtis africana	DAF/MAF
Centaurea-type	Centaurea	Others
Cerastium (Caryophyllaceae)	Cerastium	EB
Cerastium afromontanum-type	Cerastium	EB
Cerastium octandrum	Cerastium	EB
Cerealia	Cerealia	Anthropic
Chenopodiaceae	Chenopodiaceae	AA
Chenopodium	Chenopodium	AA
Cichorioideae	Cichorioideae	AA
Cissus	Cissus	ACB
Clematis	Clematis	DAF/MAF
Clutia	Clutia	DAF
Coelastrum	Coelastrum	Algae, others
Combretaceae/Melastomataceae	Combretaceae/Melastomat.	Others
Combretum	Combretum	CTW
Combretum-type aculeatum	Combretum	CTW
Combretum-type molle	Combretum molle	CTW
Commelina	Commelina	Others
Commelina-type benghalensis	Commelina	Others
Commelina-type forskalaei	Commelina	Others
Commiphora (Burseraceae)	Commiphora	ACB
Commiphora africana-type	Commiphora africana	ACB
Convolvulaceae	Convolvulaceae	Others
Corbichonia-type decumbens	Corbichonia	ACB
Corchorus (Malvaceae)	Corchorus	ACB
Corchorus-type fascicularis	Corchorus	ACB
Cordia	Cordia	ACB
Cordia abyssinica	Cordia abyssinica	ACB
Crassocephalum-type montuosum	Crassocephalum	DAF/MAF
Crateva-type adansonii	Crateva	Others
Crepis-type (Cichorioideae)	Cichorioideae	AA
Croton	Croton	ACB
Croton macrostachys	Croton macrostachys	ACB
Cucurbitaceae	Cucurbitaceae	Others
Cussonia	Cussonia	ACB
Cyathea-type manniana	Cyathea	MAF
Cyathula-type orthacantha	Cyathula	Others
Cyperaceae	Cyperaceae	Hygrophyte, excluded
Cyphostemma-type	Cyphostemma	Others
Dendrosenecio (Asteraceae)	Dendrosenecio	AA
Digera-type muricata (Amaranthaceae)	Digera	ACB

Dipsacus-type pinnatifidus	Dipsacus	AA
Dobera	Dobera	ACB
Dodonaea	Dodonaea	DAF
Dodonaea angustifolia	Dodonaea angustifolia	DAF
Dodonaea viscosa	Dodonaea viscosa	DAF
Dombeya	Dombeya	DAF
Dorstenia foetida-type	Dorstenia	DAF/MAF
Dyschoriste-type (Acanthaceae)	Dyschoriste	Others
Ebenaceae	Ebenaceae	DAF
Echinops	Echinops	EB
Ekebergia	Ekebergia	DAF/MAF
Ephedra	Ephedra	DAF
Erica	Erica	EB
Ericaceae	Ericaceae	EB
Erythrococca-type	Erythrococca	DAF/MAF
Eucalyptus	Eucalyptus	Anthropic
Euclea (Ebenaceae)	Euclea	DAF
Euphorbia	Euphorbia	ACB
Euphorbia acalyphoides	Euphorbia acalyphoides	ACB
Euphorbia hypericifolia	Euphorbia hypericifolia	ACB
Euphorbiacea	Euphorbiacea	ACB
Fabaceae	Fabaceae	Others
Ficus	Ficus	DAF/MAF
Fleurya-type aestuans (=Laportea, Urticaceae)	Fleurya	Anthropic
Flueggea	Flueggea	ACB
Flueggea-type virosa	Flueggea virosa	ACB
Galiniera coffeoides	Galiniera	DAF/MAF
Galiniera-type saxifraga (Rubiaceae)	Galiniera	DAF/MAF
Galium (Rubiaceae)	Galium	AA
Gardenia-type ternifolia	Gardenia	DAF/MAF
Gentianaceae	Gentianaceae	Others
Geraniaceae	Geraniaceae	Others
Gnidia-type chrysantha	Gnidia	DAF/MAF
Grewia-type tenax (Malvaceae, Grewioideae)	Grewia	ACB
Gunnera perpensa	Gunnera perpensa	Others
Hagenia (Rosaceae)	Hagenia	EB
Hagenia abyssinica	Hagenia abyssinica	EB
Hallea-type rubrostipulata	Hallea	MAF
Harungana (Hypericaceae)	Harungana	Others
Hebenstreitia dentata (Scrophulariaceae)	Hebenstreitia dentata	AA
Helichrysum	Helichrysum	EB
Heliotropium (Heliotropioideae)	Heliotropium	ACB

Hildebrandtia (Convolvulaceae)	Hildebrandtia	Others
Hildebrandtia obcordata	Hildebrandtia	Others
Hydrocotyle (Araliaceae)	Hydrocotyle	Hygrophyte, excluded
Hypericum	Hypericum	EB
Hypoestes (Acanthaceae)	Hypoestes	DAF
Ilex	Ilex	DAF/MAF
Ilex mitis	Ilex mitis	DAF/MAF
Impatiens	Impatiens	DAF/MAF
Indigofera (Fabaceae)	Indigofera	ACB
Ipomoea-type	Ipomoea	ACB
Iridaceae	Iridaceae	Others
Isoglossa (Acanthaceae)	Isoglossa	DAF
Jasminum	Jasminum	DAF
Jatropha	Jatropha	ACB
Juniperus	Juniperus	DAF
Juniperus procera	Juniperus procera	DAF
Justicia	Justicia	ACB
Justicia flava-type	Justicia	ACB
Justicia odora	Justicia	ACB
Kohautia (Rubiaceae)	Kohautia	ACB
Lactucoideae (Asteraceae)	Asteraceae	AA
Lamiaceae	Lamiaceae	ACB
Lannea	Lannea	ACB
Lantana	Lantana	Others
Laurembergia tetrandra (Haloragaceae)	Laurembergia tetrandra	Others
Leguminosae	Leguminosae	Others
Lemnaceae	Lemnaceae	Hygrophyte, excluded
Leonotis-type	Leonotis	EB
Lepisanthes-type senegalensis	Lepisanthes	Others
Leucas	Leucas	ACB
Liliaceae s.l.	Liliaceae	Others
Linaceae	Linaceae	Others
Lobelia	Lobelia	EB
Lythraceae	Lythraceae	Hygrophyte, excluded
Lythrum	Lythrum	Hygrophyte, excluded
Macaranga	Macaranga	DAF/MAF
Macaranga capensis	Macaranga capensis	DAF/MAF
Maerua type	Maerua	ACB
Maesa	Maesa	DAF/MAF
Maesa lanceolata-type	Maesa	DAF/MAF

Malvaceae	Malvaceae	Others
Manilkara-type butugi	Manilkara	MAF
Maytenus	Maytenus	DAF
Melastomataceae	Melastomataceae	Others
Mellera-type (Acanthaceae)	Mellera	MAF
Menispermaceae	Menispermaceae	Others
Menispermaceae cf. cissampelos	Menispermaceae	Others
Millettia-type ferruginea	Millettia	DAF/MAF
Mimulopsis (Acanthaceae)	Mimulopsis	DAF/MAF
Mimusops	Mimusops	DAF/MAF
Mimusops-type kummel	Mimusops kummel	DAF/MAF
Minuartia-type filifolia (=Arenaria)	Minuartia	AA
Monocotyledon	Monocotyledon	Others
Monolete spores (ferns)	Monolete spores	DAF/MAF
Myrica	Myrica	DAF
Myrica salicifolia	Myrica	DAF
Myriophyllum	Myriophyllum	Hygrophyte, excluded
Myrsine	Myrsine	EB
Myrsine africana	Myrsine africana	EB
Myrtaceae	Myrtaceae	Others
Mitracarpus (Rubiaceae)	Mitracarpus	Others
Nuxia congesta	Nuxia congesta	DAF/MAF
Nymphaea	Nymphaea	Hygrophyte, excluded
Oldenlandia-type (Rubiaceae)	Oldenlandia	DAF/MAF
Olea	Olea	DAF
Olea africana	Olea	DAF
Olea capensis	Olea capensis	DAF
Olea europaea ssp. africana	Olea europaea	DAF
Olea hochstetteri-type	Olea	DAF
Ormocarpum-type trichocarpum	Ormocarpum	ACB
Pappea capensis	Pappea capensis	Others
Paronychieae	Paronychieae	Others
Pavetta	Pavetta	DAF
Pavetta-type abyssinica	Pavetta	DAF
Pediastrum boryanum	Pediastrum boryanum	Algae, others
Pediastrum duplex	Pediastrum duplex	Algae, others
Pentanisia-type ouranogyne (Rubiaceae)	Pentanisia	ACB
Phoenix	Phoenix	DAF/MAF
Phoenix reclinata-type	Phoenix	DAF/MAF
Phyllanthus	Phyllanthus	Others
Phyllanthus-type amarus	Phyllanthus	Others
Phytolacca	Phytolacca	DAF/MAF

Pilea-type bambuseti (Urticaceae)	Pilea	Anthropic
Pinus	Pinus	DAF
Pittosporum	Pittosporum	DAF/MAF
Pittosporum abyssinicum-type	Pittosporum abyssinicum	DAF/MAF
Plantago	Plantago	Anthropic
Plantago africana-type	Plantago	Anthropic
Plantago coronopus-type	Plantago	Anthropic
Plantago lanceolata	Plantago lanceolata	Anthropic
Poaceae	Poaceae	Others
Podocarpus	Podocarpus	DAF
Podocarpus falcatus	Podocarpus falcatus	DAF
Polygala	Polygala	Others
Polygonum	Polygonum	AA
Polygonum plebeium-type	Polygonum	AA
Polygonum senegalense-type	Polygonum	AA
Polypodiaceae s.l.	Polypodiaceae	Others
Polyscias (Araliaceae)	Polyscias	CTW
Polyscias fulva	Polyscias fulva	CTW
Portulacaceae	Portulacaceae	Others
Potamogeton	Potamogeton	Hygrophyte, excluded
Prosopis (Fabaceae)	Prosopis	ACB
Protea-type	Protea	DAF
Proteaceae	Proteaceae	DAF
Prunus	Prunus	DAF/MAF
Prunus africana	Prunus africana	DAF/MAF
Psilotrichum-type elliottii (Amaranthaceae)	Psilotrichum	Other
Psydrax	Psydrax	Others
Psydrax-type schimperiana	Psydrax	Others
Pterocephalus-type frutescens	Pterocephalus	DAF
Pterolobium	Pterolobium	Others
Pterolobium stellatum	Pterolobium stellatum	Others
Pupalia-type lappacea (Amaranthaceae)	Pupalia	ACB
Ranunculus	Ranunculus	AA
Rananculus oreophytus-type	Rananculus	AA
Ranunculus stagnalis-type	Ranunculus	AA
Rapanea (=Myrsine)	Rapanea (=Myrsine)	EB
Rapanea melanophloeos	Rapanea melanophloeos	EB
Resedaceae	Resedaceae	Anthropic
Rhamnaceae	Rhamnaceae	Others
Rhus	Rhus	DAF
Ricinus	Ricinus	Others
Ricinus communis	Ricinus communis	Others

Rosa	Rosa	EB
Rosa abyssinica-type	Rosa	EB
Rosaceae	Rosaceae	Others
Rubia	Rubia	Others
Rubiaceae	Rubiaceae	Others
Rubus	Rubus	DAF
Rubus pinnatus-type	Rubus	DAF
Rumex	Rumex	Anthropic
Salix	Salix	Others
Salix subserrata-type	Salix	Others
Salvadora persica	Salvadora persica	ACB
Sapindaceae	Sapindaceae	Others
Sapotaceae	Sapotaceae	DAF/MAF
Sapium ellipticum	Sapium ellipticum	MAF
Satureja (Lamiaceae)	Satureja	AA
Schefflera	Schefflera	DAF/MAF
Schefflera abyssinica-type	Schefflera abyssinica	DAF/MAF
Schefflera myriantha-type	Schefflera myriantha	DAF/MAF
Schefflera volkensii	Schefflera volkensii	DAF/MAF
Scrophulariaceae	Scrophulariaceae	Others
Sebaea-type brachyphylla (Gentianaceae)	Sebaea	AA
Securinega	Securinega	DAF
Senecio	Senecio	AA
Senna	Senna	ACB
Sesbania	Sesbania	Others
Silene-type burchellii	Silene	AA
Simaroubaceae	Simaroubaceae	DAF
Solanum	Solanum	Others
Spiniluma-type oxyacantha (=Sideroxylon)	Spiniluma (=Sideroxylon)	DAF
Steganotaenia-type	Steganotaenia	Others
Stoebe (Asteraceae)	Stoebe	AA
Stoebe kilimandscharica-type	Stoebe	AA
Suaeda-type monoica	Suaeda	ACB
Swertia (Gentianaceae)	Swertia	AA
Swertia lastii-type	Swertia	AA
Swertia-type kilimandscharica	Swertia	AA
Syzygium	Syzygium	DAF/MAF
Tacazzea-type apiculata	Tacazzea	DAF/MAF
Tamarindus	Tamarindus	ACB
Tamarix	Tamarix	DSS
Tapinanthus (Loranthaceae)	Tapinanthus	DAF/MAF
Tarenna type (Rubiaceae)	Tarenna	Others
Tarenna-type graveolens	Tarenna	Others

Teclea	Teclea	DAF
Terminalia	Terminalia	CTW
Thalictrum (Ranunculaceae)	Thalictrum	EB
Thymelaeaceae	Thymelaeaceae	others
Tiliaceae	Tiliaceae	others
Trema-type orientalis	Trema	DAF
Tribulus	Tribulus	others
Trichocladus ellipticus-type	Trichocladus	DAF
Trichodesma zeylanicum-type (Boraginaceae)	Trichodesma	others
Trifolium-type acaule	Trifolium	others
Trilepisium (Moraceae)	Trilepisium	MAF
Trilepisium-type madagascariensis	Trilepisium	MAF
Trilete spores	Trilete spores	DAF/MAF
Typha	Typha	Hygrophyte, excluded
Typha latifolia	Typha	Hygrophyte, excluded
Typha type-angustifolia	Typha	Hygrophyte, excluded
Uebelinia-type (=Silene)	Uebelinia (=Silene)	AA
Urticaceae	Urticaceae	Anthropic
Vahlia somalensis-type (=Dombeya)	Vahlia (=Dombeya)	DAF
Vangueria-type acutiloba	Vangueria	DAF
Vepris-type daniellii	Vepris	DAF/MAF
Vernonia (Asteraceae)	Vernonia	DAF/MAF
Vernonieae	Vernonieae	DAF/MAF
Vitaceae	Vitaceae	others
Ximenia (Olacaceae)	Ximenia	ACB
Xyris (Xyridaceae)	Xyris	others
Zanthoxylum	Zanthoxylum	DAF
Zanthoxylum-type usambarense	Zanthoxylum	DAF
Zea mays	Zea mays	Anthropic
Ziziphus	Ziziphus	ACB
